# Epidemiological trends of subarachnoid hemorrhage at global, regional, and national level: a trend analysis study from 1990 to 2021

**DOI:** 10.1186/s40779-024-00551-6

**Published:** 2024-07-11

**Authors:** Bin Lv, Jin-Xin Lan, Yan-Fang Si, Yi-Fan Ren, Ming-Yu Li, Fang-Fang Guo, Ge Tang, Yang Bian, Xiao-Hui Wang, Rong-Ju Zhang, Zhi-Hua Du, Xin-Feng Liu, Sheng-Yuan Yu, Cheng-Lin Tian, Xiang-Yu Cao, Jun Wang

**Affiliations:** 1 Department of Neurology, the First Medical Center, Chinese PLA General Hospital, Beijing, 100853 China; 2 School of Medicine, Nankai University, Tianjin, 300071 China; 3 Department of Ophthalmology, the Eighth Medical Center, Affiliated to the Senion Department of Ophthalmology, the Third Medical Center, Chinese PLA General Hospital, Beijing, 100091 China; 4 Department of Internal Medicine, Gucheng County Hospital of Traditional Chinese Medicine, Hengshui, Hebei 253800 China; 5 Department of Outpatient, No.13 Cadre Santatorium of Beijing Garrison, Beijing, 100393 China; 6 Department of Neurology, Yongchuan Hospital Affiliated of Chongqing Medical University, Chongqing, 402160 China

**Keywords:** Subarachnoid hemorrhage (SAH), Global Burden of Disease Study (GBD) 2021, Incidence, Mortality, Disability-adjusted life-years (DALYs)

## Abstract

**Background:**

Subarachnoid hemorrhage (SAH) is a subtype of hemorrhagic stroke characterized by high mortality and low rates of full recovery. This study aimed to investigate the epidemiological characteristics of SAH between 1990 and 2021.

**Methods:**

Data on SAH incidence, mortality, and disability-adjusted life-years (DALYs) from 1990 to 2021 were obtained from the Global Burden of Disease Study (GBD) 2021. Estimated annual percentage changes (EAPCs) were calculated to evaluate changes in the age-standardized rate (ASR) of incidence and mortality, as well as trends in SAH burden. The relationship between disease burden and sociodemographic index (SDI) was also analyzed.

**Results:**

In 2021, the incidence of SAH was found to be 37.09% higher than that in 1990; however, the age-standardized incidence rates (ASIRs) showed a decreased [EAPC: -1.52; 95% uncertainty interval (UI) -1.66 to -1.37]. Furthermore, both the number and rates of deaths and DALYs decreased over time. It was observed that females had lower rates compared to males. Among all regions, the high-income Asia Pacific region exhibited the highest ASIR (14.09/100,000; 95% UI 12.30/100,000 − 16.39/100,000) in 2021, with an EPAC for ASIR < 0 indicating decreasing trend over time for SAH ASIR. Oceania recorded the highest age-standardized mortality rates (ASMRs) and age-standardized DALYs rates among all regions in 2021 at values of respectively 8.61 (95% UI 6.03 − 11.95) and 285.62 (95% UI 209.42 − 379.65). The burden associated with SAH primarily affected individuals aged between 50 − 69 years old. Metabolic risks particularly elevated systolic blood pressure were identified as the main risk factors contributing towards increased disease burden associated with SAH when compared against environmental or occupational behavioral risks evaluated within the GBD framework.

**Conclusions:**

The burden of SAH varies by gender, age group, and geographical region. Although the ASRs have shown a decline over time, the burden of SAH remains significant, especially in regions with middle and low-middle SDI levels. High systolic blood pressure stands out as a key risk factor for SAH. More specific supportive measures are necessary to alleviate the global burden of SAH.

**Supplementary Information:**

The online version contains supplementary material available at 10.1186/s40779-024-00551-6.

## Background

The occurrence of subarachnoid hemorrhage (SAH) is a devastating cerebrovascular event that accounts for 5% of all strokes, with a mortality rate of 25% and disability rate of 66% [1–3]. On average, SAH patients are younger by 20 years compared to those suffering from ischemic stroke, resulting in an extended period of chronicity and disability that requires greater social resources [2]. In the United States, the management cost per patient for SAH accounted for over 82,000 dollars from 2002 to 2014, contributing to an estimated annual nationwide financial burden ranging from $1.7 to $5.6 billion of dollars [4]. Therefore, comprehensive and up-to-date epidemiological data are needed for policymakers and scholars to develop responsive strategies. While several studies have reported on the regional epidemiological characteristics of aneurysmal SAH (a subtype of SAH) [5–7], there is currently a lack of updated global data on SAH.

The Global Burden of Disease Study (GBD) 2021 provides a unique framework for evaluating the disease burden using multiple factors [8, 9]. Previous studies conducted by GBD collaborators have demonstrated that more than 90% of the stroke burden can be attributed to environmental, physiological, and lifestyle risk factors [8, 9]. To gain a better understanding of the policy implications associated with dynamic epidemiological data on SAH trends from 1990 to 2021, this study presents updated data extracted from the GBD 2021.

## Methods

### Data source

The GBD 2021 examined over 370 diseases and injuries in 204 countries and regions, providing detailed data on incidence, mortality, disability-adjusted life-years (DALYs), and age-standardized rates (ASRs) [10]. All data were meticulously sourced from reputable public databases after a thorough screening to ensure quality assurance. The GBD authors commit to annual updates for accuracy [11]. Data collected by the GBD collaborator network underwent rigorous cleaning, transformation, and modeling processes conducted by research organizations worldwide to generate estimates (https://www.healthdata.org/data-tools-practices/data-collection). For modeling SAH, the GBD collaborator network identified outliers among International Classification of Disease (ICD)-8 datapoints that deviated significantly from the rest of the dataset, resulting in implausible time trends. Additionally, vital registration data from China Tibet, Ghana, and Cabo Verde exhibited unrealistically high values across all years and age groups; similarly low values were observed in Tajikistan and Palestine [12].

The number of deaths and mortality rate were mainly estimated using the cause-of-death ensemble model approach, while the number of diseases and prevalence were estimated using the Bayesian Meta-regression model. Disability weight is defined as the measure of health loss severity or nonfatal disability severity. Years lived with disability (YLD) were calculated by multiplying the number of patients by the duration until remission or death, along with the disability weight. The years of life lost (YLL) are calculated by multiplying the number of deaths by the corresponding standard life expectancy derived from a reference life table. The DALY is defined as the total number of healthy years lost from onset to death, which is the sum of the YLL and YLD [13]. The DALY serves as a crucial parameter for assessing disease burden.

The sociodemographic index (SDI) is a composite indicator that reflects a country’s level of development based on lag-distributed income per capita, the average educational attainment of individuals aged over 15 years, and the total fertility rate among those under the age of 25 years. It is strongly correlated with health outcomes. The SDI ranges from 0 to 1, where 0 represents the minimum level of development and 1 represents the maximum level [10]. Based on their SDI values, the 204 countries and regions were categorized into 5 groups: high-SDI, medium–high-SDI, medium-SDI, medium–low-SDI, and low-SDI regions. Patient age was classified into 17 subgroups: ages 0 − 14, ages 15 − 19, ages 20 − 24, ages 25 − 29, ages 30 − 34, ages 35 − 39, ages 40 − 44, ages 45 − 49, ages 50 − 54, ages 55 − 59, ages 60 − 64, ages 65 − 69, ages 70 − 74, ages 75 − 79, ages 80 − 84, ages 85 − 89, ages 90 − 94, and ages > 95 years.

### Estimation of risk factor

The attributable risk factors were assessed across 4 levels [14]. The analyzed risk factors included particulate matter pollution, high and low temperatures, lead exposure, smoking, secondhand smoke exposure, diet high in red meat consumption, high-sodium diet, low-fiber diet, diet low in fruits, diet low in vegetables, high body mass index, and high systolic blood pressure (Additional file 1: Table S1). The percentage of SAH-related deaths and DALYs can be found in the GBD results tool. Previously established definitions of these risk factors were utilized in this study [14–16] (Additional file 1: Table S2).

### Definition of SAH

SAH involves the leakage of blood into the subarachnoid space [17]. In the GBD 2021, SAH was defined according to the ICD, 10th revision (codes I60.0 − I60.9, I62.0, I67.0 − I67.1 and I69.0) and ICD-9 (430.0 − 430.9).

### Statistical analyses

The burden of SAH was quantified over time and by region, sex, and age using ASR, which includes age-standardized incidence rates (ASIRs), age-standardized mortality rates (ASMRs), and age-standardized disability-adjusted life-year rates (ASDRs; Additional file 1: Fig. S1**)**. These measures take into account variations in population age compositions. Age standardization aims to eliminate the impact of population age composition and ensure the comparability of research indicators. In the GBD database, these indicators are estimated using the world population age standard calculated with the following formula: ASR = $\frac{{\sum }_{{\varvec{i}}=1}^{{\varvec{A}}}{{\varvec{a}}}_{{\varvec{i}}}{{\varvec{w}}}_{{\varvec{i}}}}{{\sum }_{{\varvec{i}}=1}^{{\varvec{A}}}{{\varvec{w}}}_{{\varvec{i}}}}$×100,000, which is the sum of the age-specific rates (*a*_*i*_, where *i* represents the *i*^th^ age class) and the number of persons (or weight) (*w*_*i*_) in the same age subgroup *i* of the selected reference standard population, dividing the sum of the standard population.

Furthermore, the estimated annual percentage changes (EAPCs) were calculated to evaluate the annual average change in ASR using a generalized linear regression model. This model captures the evolving pattern of ASR over time by establishing a relationship between the natural logarithm (ln) of ASR and time through the equation: *Y* = *α* + *βX* + *ε*.

Here, *Y* refers to ln(ASR), *α* denotes the intercept, *X* signifies the calendar year, *ε* represents the error term, and *β* reflects a linear positive or negative trend in ASR. The EAPC and its 95% confidence interval (CI) were computed using this formula: EAPC = 100 × [exp (β) − 1].

A positive EAPC with a lower limit of 95% CI indicates an upward trend in ASR. In brief, the EAPC and its 95% uncertainty interval (UI) were determined utilizing the formula: EAPC = 100 × {exp [$\frac{\mathbf{ln}\left(\mathbf{A}\mathbf{S}\mathbf{R}\right)-\boldsymbol{\alpha }-{\varvec{\varepsilon}}}{{\varvec{y}}{\varvec{e}}{\varvec{a}}{\varvec{r}}}$] − 1}, where ln(ASR) refers to the natural logarithm (ln) of ASR. Conversely, a negative EAPC with an upper limit of 95% CI indicates a downward trend in ASR [18].

The estimates and 95% UIs for metrics used to assess the burden of SAH were derived from the GBD 2021 database (https://ghdx.healthdata.org/gbd-2021). All statistical analyses were performed using R software (version 4.0.3; Bell Laboratories, formerly AT&T, now Lucent Technologies). GraphPad Prism (version 9.5.0 for Windows, GraphPad Software, San Diego, California USA, https://www.graphpad.com/) was utilized for conducting *t*-tests and analysis of variance to compare the metrics across different sexes, ages, and regions. All hypothesis tests were two-sided with a significance level set at *P* < 0.05 [19].

## Results

### Global incidence, mortality, and DALYs

In 2021, there were 697.49 × 10^3^ incident cases of SAH, with a 95% UI ranging from 614.33 × 10^3^ to 795.79 × 10^3^, representing a significant increase of 37.09% compared to the year 1990. The ASIR of SAH in 2021 was recorded at a lower value of 8.32/100,000 (95% UI 7.34/100,000 − 9.48/100,000) as opposed to the rate in the year 1990 which stood at 11.69/100,000 (95% UI 10.22/100,000 − 13.50/100,000). Furthermore, the EAPC indicated that ASIR experienced a decline during the period between 1990 and 2021 [-1.52 (95% UI -1.66 to -1.37)], resulting in an overall decrease of 28.82% in ASIR from 1990 to 2021. Among female individuals, the incidence of SAH cases in 2021 was 356.64 × 10^3^ (95% UI 315.06 × 10^3^ − 408.98 × 10^3^), which showed an increase of 8.19% compared to male individuals [340.85 × 10^3^ (95% UI 298.16 × 10^3^ − 388.88 × 10^3^)]. However, the female population exhibited a lower proportion of increased incident cases (34.54% vs. 39.86%) and EAPC of ASIR (-1.53 vs. -1.52) than their male counterparts did. From 1990 to 2021, there was an upward trend in SAH incidence while the ASIR of SAH declined.

The mortality rate of SAH decreased by 3.98% from 1990 to 2021. In 2021, the number of SAH deaths and the ASMR were estimated at 352.81 × 10^3^ (95% UI 309.02 − 401.47) and 4.18 (95% UI 3.66 − 4.76), respectively. Over the study period, the ASMR for SAH declined from 9.54/100,000 (95% UI 6.80/100,000 − 11.91/100,000) in 1990 to 4.18/100,000 (95% UI 3.66/100,000 − 4.76/100,000) in 2021 with a decrease of 5.89% and an EAPC of -3.08 (95% CI -3.30 to -2.86). Although female individuals had slightly higher death rates than males did [179.06 × 10^3^ (95% UI 156.26 × 10^3^ − 208.10 × 10^3^) vs. 173.75 × 10^3^ (95% UI 140.71 × 10^3^ − 217.57 × 10^3^)], their ASMR was lower [3.91/100,000 (95% UI 3.41/100,000 − 4.55/100,000) vs. 4.48/100,000 (95% UI 3.64/100,000 − 5.56/100,000)]. Overall, the ASMR for SAH significantly decreased from 1990 to 2021, and females exhibited a lower ASMR compared with males.

Globally, the number of DALYs decreased from 1203.13 × 10^3^ (95% UI 940.98 × 10^3^ − 1450.79 × 10^3^) in 1990 to 1064.19 × 10^3^ (95% UI 939.90 × 10^3^ − 1212.13 × 10^3^) in 2021, representing a reduction of -11.55%. The ASDR of SAH also exhibited a decline, with an EAPC of -2.88 (95% CI -3.06 to -2.70). Notably, the decrease in DALYs among females showed a significantly greater EAPC [-3.00 (95% CI -3.20 to -2.80)] compared to males [-2.78 (95% CI -2.95 to -2.61); Table 1].

**Table 1:** Global incidence, deaths, and DALYs of subarachnoid hemorrhage (SAH) from 1990 to 2021

Year	Both	Male	Female
1990			
Incidence/1000 (95% UI)	508.79 (441.50 − 587.62)	243.71 (209.70 − 281.37)	265.08 (229.34 − 305.77)
Deaths/1000 (95% UI)	374.89 (270.97 − 465.03)	180.96 (96.59 − 243.467)	193.93 (136.30 − 238.46)
DALYs/1000 (95% UI)	1203.13 (940.98 − 1450.79)	610.34 (367.08 − 782.82)	592.78 (455.26 − 704.56)
ASIR/100,000 persons (95% UI)	11.69 (10.22 − 13.50)	11.72 (10.16 − 13.59)	11.74 (10.21 − 13.57)
ASMR/100,000 persons (95% UI)	9.54 (6.80 − 11.91)	9.99 (5.13 − 13.57)	9.13 (6.31 − 11.27)
ASDR/100,000 persons (95% UI)	275.85 (213.22 − 335.43)	288.69 (169.19 − 374.61)	263.23 (201.20 − 313.47)
2021			
Incidence /1000 (95% UI)	697.49 (614.33 − 795.79)	340.85 (298.16 − 388.88)	356.64 (315.06 − 408.98)
Death/1000 (95% UI)	352.81 (309.02 − 401.47)	173.75 (140.71 − 217.57)	179.06 (156.26 − 208.10)
DALYs/1000 (95% UI)	1064.19 (939.90 − 1212.13)	548.35 (449.85 − 690.08)	515.84 (461.74 − 589.00)
ASIR/100,000 persons (95% UI)	8.32 (7.34 − 9.48)	8.51 (7.48 − 9.65)	8.17 (7.21 − 9.35)
ASMR/100,000 persons (95% UI)	4.18 (3.66 − 4.76)	4.48 (3.64 − 5.56)	3.91 (3.41 − 4.55)
ASDR/100,000 persons (95% UI)	125.20 (110.54 − 142.61)	134.07 (109.87 − 167.87)	116.35 (104.22 − 133.10)
1990 − 2021			
Incidence (%)	37.09	39.86	34.54
Deaths (%)	-5.89	-3.98	-7.67
DALYs (%)	-11.55	-10.16	-12.98
EAPC of ASIR (95% CI)	-1.52 (-1.66 to -1.37)	-1.52 (-1.69 to -1.35)	-1.53 (-1.66 to -1.41)
EAPC of ASMR (95% CI)	-3.08 (-3.30 to -2.86)	-2.99 (-3.20 to -2.77)	-3.19 (-3.43 to -2.96)
EAPC of ASDR (95% CI)	-2.88 (-3.06 to -2.70)	-2.78 (-2.95 to -2.61)	-3.00 (-3.20 to -2.80)

*DALYs* disability-adjusted life-years, *ASIR* age-standardized incidence rate, *ASMR* age-standardized mortality rate, *ASDR* age-standardized disability-adjusted life-year rate, *EAPC* estimated annual percentage change, *UI* uncertainty interval, *CI* confidence interval

### Regional incidence, mortality, and DALYs

**Fig. 1:**
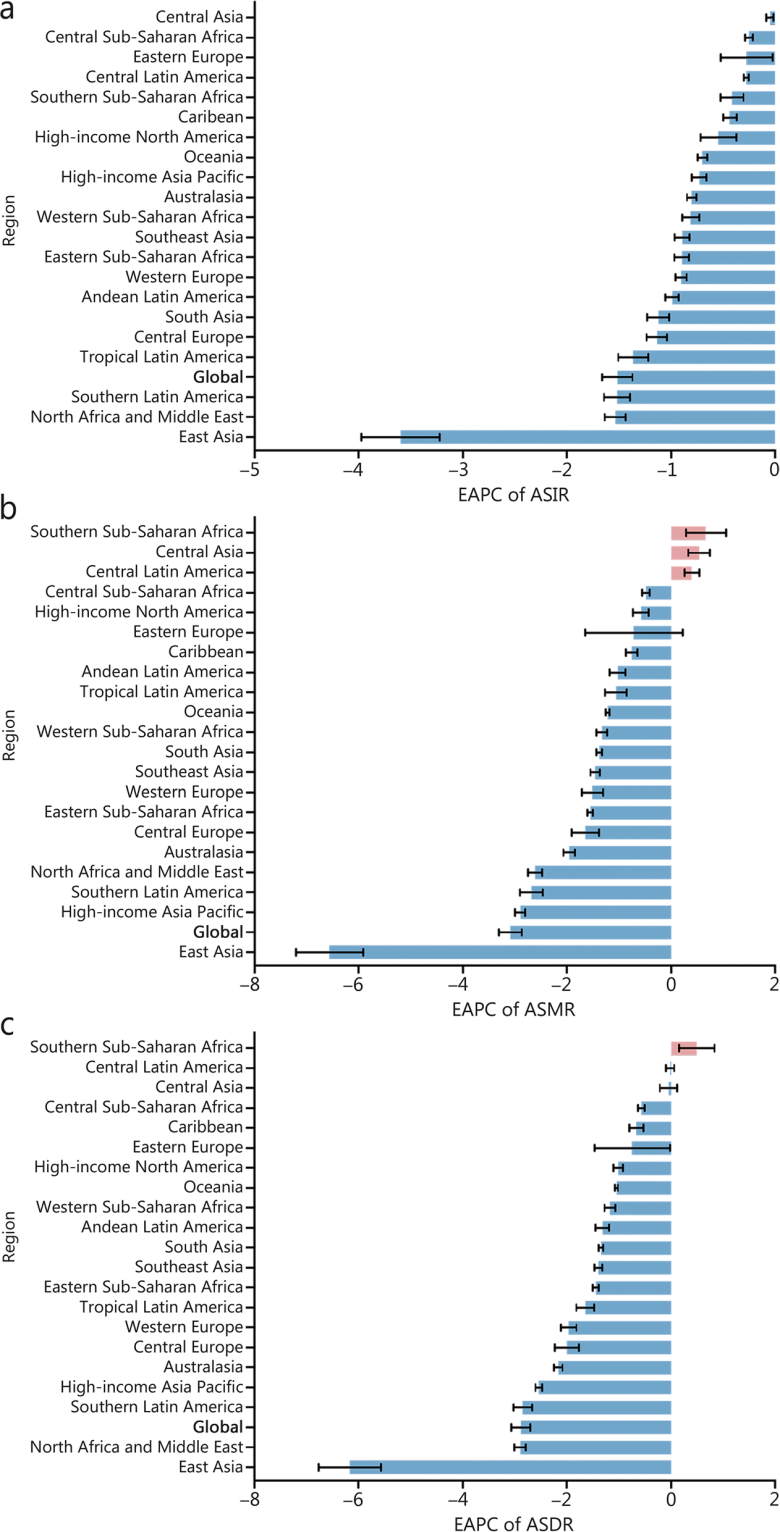
EAPCs of the ASRs for SAH. **a** EAPCs of the ASIR for SAH in 21 regions. **b** EAPCs of the ASMR for SAH in 21 regions. **c** EAPCs of the ASDR for SAH in 21 regions. ASR age-standardized rate, ASIR age-standardized incidence rate, ASMR age-standardized mortality rate, ASDR age-standardized disability-adjusted life-year rate, EAPC estimated annual percentage change, SAH subarachnoid hemorrhage

In 2021, the 5 regions with the highest incidence of SAH were East Asia, South Asia, Southeast Asia, high-income Asia Pacific, and Western Europe (Additional file 1: Table S3). Among these regions, the high-income Asia Pacific region exhibited the highest ASIR for SAH at 14.09 per 100,000 populations (95% UI 12.30/100,000 − 16.39/100,000; Additional file 1: Table S4). The ASIR for SAH in all regions showed a statistically significant decline (EPAC < 0) as depicted in Fig. 1a. Notably, East Asia experienced a substantial decrease in ASIR for SAH from 1990 to 2021 with an EAPC of -3.60 (95% CI -3.97 to -3.22). Furthermore, it was observed that the decline in ASIR for SAH was more pronounced among females compared to males across most regions (Additional file 1: Tables S4, S5, and Fig. S2a).

In the East Asia region, SAH resulted in the highest number of deaths [95.18 × 10^3^ (95% UI 70.28 × 10^3^ − 119.16 × 10^3^); Additional file 1: Table S3]. The highest ASMR was recorded in Oceania [8.61/100,000 (95% UI 6.03/100,000 − 11.95/100,000)], followed by Southeast Asia (6.02/100,000; 95% UI 5.02/100,000 − 8.42/100,000), and Andean Latin America [6.01/100,000 (95% UI 4.87/100,000 − 7.29/100,000)]. The largest decline in ASMR for SAH occurred in East Asia [-6.56 (95% CI -7.21 to -5.91)]. Except for Central Latin America, Central Asia, and Southern Sub-Saharan Africa, all regions had a decreasing trend in ASMR (Fig. 1b). However, in high-income North America, the ASMR slightly increased among males and decreased among females, and in other regions, the tendency of ASMR was consistent between both sexes (Additional file 1: Tables S4, S5 and Fig. S2b).

Similar to the incidence and mortality rates, East Asia exhibited the highest DALYs for SAH [2396.95 × 10^3^ (95% UI 1840.65 × 10^3^ – 2934.11 × 10^3^)]. The highest ASDR for SAH was observed in Oceania at a rate of 285.62/100,000 (95% UI 209.42/100,000 − 379.65/100,000), followed by Andean Latin America with a rate of 199.49/100,000 (95% UI 166.06/100,000 − 239.00/100,000), and the Caribbean with a rate of 191.37/100,000 (95% UI 146.14/100,000 − 234.04/100,000) (Additional file 1: Table S4). With the exception of Southern Sub-Saharan Africa which experienced no change in ASDR from 1990 to 2021, the remaining regions showed a decreasing trend; notably, East Asia demonstrated the largest decrease with an EAPC of -6.17 (95% CI -6.76 to -5.57) (Fig. 1c). The ASDR displayed negative trends among females but positive trends among males in Central Asia and Central Latin America (Additional file 1: Table S5, Fig. S2c).

The EAPCs for ASIR, ASMR, and ASDR were generally higher at regional levels compared to the global level. Although East Asia had the highest number of incident cases, mortality, and DALYs, the ASIR, ASDR, and ASMR in this region showed the most significant decline from 1990 to 2021 (Table 1).

### National incidence, mortality, and DALYs

In 2021, the top 3 countries with the highest incidence of SAH were China [145,138.48 (95% UI 125,425.42 − 169,016.38)], India [101,503.37 (95% UI 86,895.35 − 118,265.62)], and Japan [37,011.29 (95% UI 32,479.52 − 42,503.85)] (Additional file 1: Table S6). Solomon Island [24.22/100,000 (95% UI 21.64/100,000 − 27.07/100,000)], Kiribati [22.01/100,000 (95% UI 19.58/100,000 – 24.58/100,000)], and Marshall Island [20.18/100,000 (95% UI 18.03/100,000 − 22.51/100,000)] had the highest ASIRs for SAH (Additional file 1: Table S7). From 1990 to 2021, the Philippines (EAPC = 1.17; 95% CI 0.96 − 1.37), Turkmenistan (EAPC = 1.04; 95% CI 0.93 − 1.15), and Zimbabwe (EAPC = 0.98; 95% CI 0.84 − 1.11) experienced the strongest increase in the ASIR of SAH. Philippines, Turkmenistan, Zimbabwe, Georgia, Tajikistan, Lesotho, Dominican Republic, Solomon Islands, Mongolia, Kiribati, Uzbekistan, North Macedonia, Greenland, Oman, Bulgaria, France, Kazakhstan, Mozambique, Honduras and Vanuatu exhibited an increasing trend in ASIR with EAPC > 0 from 1990 to 2021. In all other countries, the ASIR decreased from 1990 to 2021. China, Iraq, Republic of Korea experienced the greatest decrease in ASIR (Additional file 1: Table S8).

China [91,802.18 (95% UI 66,671.88 − 116,215.44)] and India [48,284.62 (95% UI 33,671.85 − 66,531.02)] reported the highest number of deaths in 2021 (Additional file 1: Table S6). The 3 countries with the highest ASMRs for SAH were Nauru [12.87/100,000 (95% UI 9.14/100,000 − 17.22/100,000)], Haiti [12.78/100,000 (95% UI 6.59/100,000 − 20.85/100,000)], and Mongolia [12.41/100,000 (95% UI 9.07/100,000 − 16.05/100,000)] (Additional file 1: Table S7). Georgia (EAPC = 2.74; 95% CI 1.79 − 3.69), Zimbabwe (EAPC = 2.18; 95% CI 1.57 − 2.78), and Uzbekistan (EAPC = 1.96; 95% CI 1.03 − 2.90) experienced the largest increase in ASMR of SAH (Additional file 1: Table S8). In contrast, China (EAPC = -6.66; 95% CI -7.32 to -6.01), Lebanon (EAPC = -4.88; 95% CI -5.17 to -4.58), and the Republic of Korea (EAPC = -4.78; 95% CI -4.97 to -4.58) witnessed the greatest decrease in AMSR of SAH (Additional file 1: Table S8).

The highest DALYs were recorded in China [2,296,534.29 (95% UI 1,727,441.71 − 2,847,370.05)], India [1,657,353.60 (95% UI 1,212,105.37 − 2,198,269.51)], and Indonesia [542,472.01 (95% UI 421,992.91 − 753,770.57)] (Additional file 1: Table S6). Nauru [476.38/100,000 (95% UI 352.58/100,000 − 635.27/100,000)], Haiti [434.98/100,000 (95% UI 237.15/100,000 − 658.46/100,000)], and the Marshall Islands [431.34/100,000 (95% UI 286.73/100,000 − 619.46/100,000)] had the highest ASDRs for SAH (Additional file 1: Table S7). The greatest increase in ASDR occurred in Zimbabwe (EPAC = 2.38; 95% CI 1.75 − 3.01), Georgia (EPAC = 1.82; 95% CI 1.09 − 2.55), and Lesotho (EPAC = 1.76; 95% CI 1.37 − 2.16). However, China had the greatest decrease in ASDR with an EPAC of -6.29 (95% CI -6.89 to -5.69). Furthermore, China and India had the largest number of incident cases, deaths, and DALYs. Nauru and Haiti had the highest ASMR and ASDR for SAH (Additional file 1: Table S8).

### Burden of SAH based on SDI

**Fig. 2:**
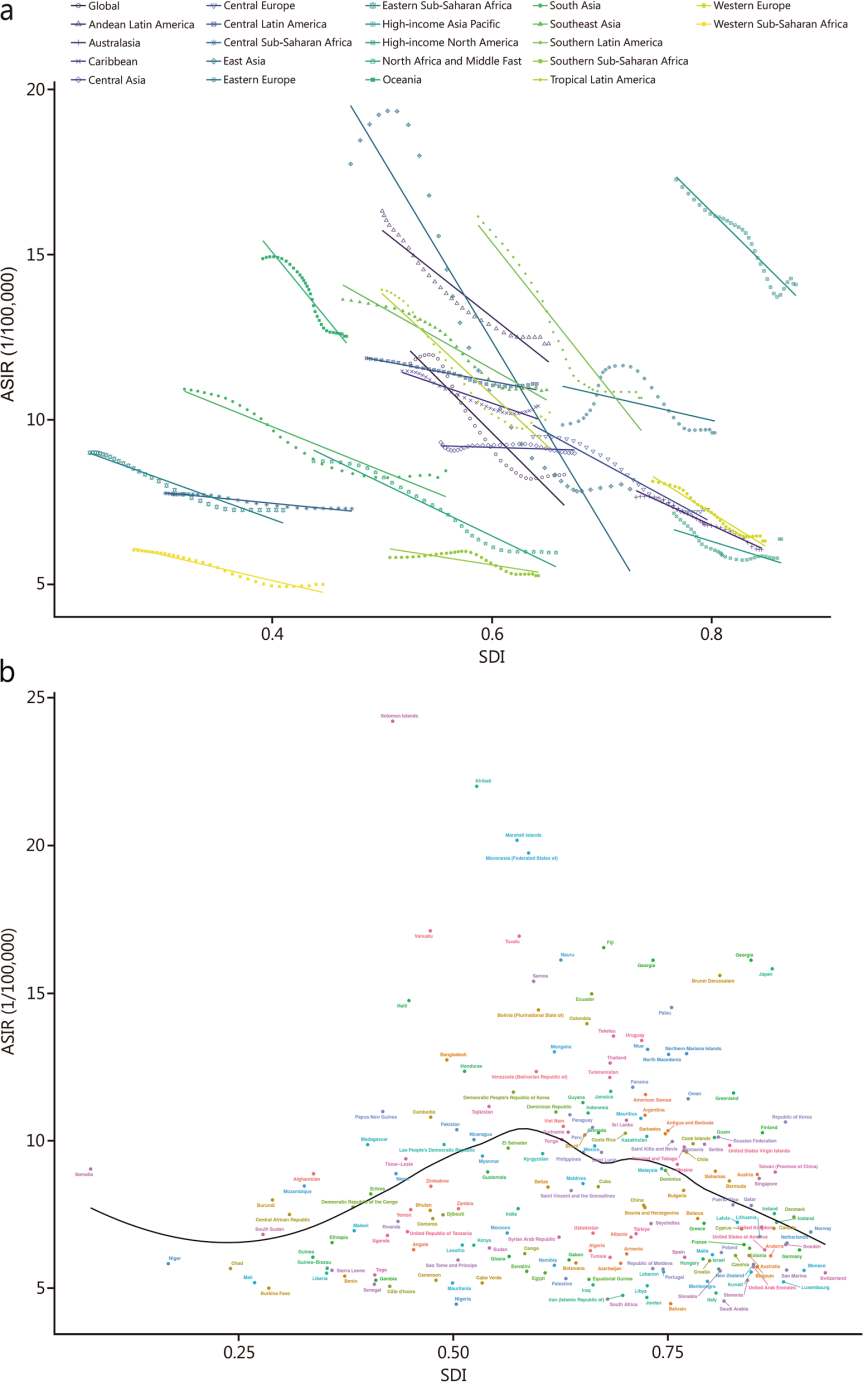
ASIRs for SAH of 21 regions and 204 countries and territories by SDI. **a** ASIRs for SAH of 21 regions from 1990 − 2021 according to the SDI. **b** ASIRs for SAH of 204 countries and territories in 2021 according to the SDI. ASIR age-standardized incidence rate, SAH subarachnoid hemorrhage, SDI sociodemographic index

The majority of incident cases, deaths, and DALYs were predominantly observed in regions with middle and low-middle SDI levels (Table 2). The ASIR and ASDR exhibited a negative correlation with the SDI across all regions (Fig. 2a, Additional file 1: Fig. S3a). Similarly, the ASMR showed a negative correlation with the SDI in most regions, except for Eastern Europe (Additional file 1: Fig. S3b). At the national level, there was a positive correlation between ASIR, ASDR ASMR, and SDI at low and middle SDI levels in 2021; however, this correlation became negative at high SDI levels across 204 countries and territories (Fig. 2b; Additional file 1: Figs. S3, S4).

**Table 2:** EAPC of ASIR, ASDR, and ASMR for SAH in countries with five SDI levels from 1990 to 2021

Region	ASIR/100,000 persons (95% UI) (1990/2021)	EAPC of ASIR (95% CI)	ASMR/100,000 persons (95% UI)(1990/2021)	EAPC of ASMR (95% CI)	ASDR/100,000 persons (95% UI) (1990/2021)	EAPC of ASDR (95% CI)
Low SDI	9.03 (7.77 − 10.65)/7.41 (6.46 − 8.56)	-0.86 (-0.93 to -0.79)	5.22 (2.56 − 9.37)/3.79 (2.06 − 7.35)	-1.11 (-1.17 to -1.05)	166.67 (92.18 − 281.66)/120.87 (71.27 − 221.66)	-1.13 (-1.18 to -1.07)
Low-middle SDI	10.99 (9.56 − 12.8)/8.86 (7.79 − 10.17)	-0.92 (-1.00 to -0.84)	7.18 (4.63 − 10.96)/4.90 (3.77 − 6.29)	-1.25 (-1.29 to -1.21)	230.87 (161.91 − 324.13)/154.18 (122.57 − 193.50)	-1.30 (-1.34 to -1.27)
Middle SDI	14.85 (12.89 − 17.36)/9.16 (8.08 − 10.43)	-2.22 (-2.47 to -1.97)	17.91 (9.50 − 23.15)/5.23 (4.29 − 6.08)	-4.69 (-5.15 to -4.23)	446.97 (266.42 − 560.45)/143.43 (122.51 − 161.79)	-4.22 (-4.60 to -3.84)
High-middle SDI	11.58 (10.18 − 13.33)/7.42 (6.59 − 8.35)	-1.96 (-2.14 to -1.78)	9.27 (7.04 − 11.34)/3.52 (3.09 − 4.15)	-3.65 (-3.88 to -3.41)	263.48 (206.45 − 313.81)/99.55 (89.94 − 115.08)	-3.62 (-3.82 to -3.41)
High SDI	10.12 (8.83 − 11.76)/7.85 (6.91 − 8.97)	-0.99 (-1.06 to -0.92)	5.25 (4.94 − 5.51)/2.89 (2.64 − 3.04)	-2.04 (-2.17 to -1.92)	173.04 (164.56 − 180.79)/91.84 (86.01 − 97.38)	-2.16 (-2.25 to -2.07)

*ASIR* age-standardized incidence rate, *ASMR* age-standardized mortality rate, *ASDR* age-standardized disability-adjusted life-year rate, *EAPC* estimated annual percentage change, *SAH* subarachnoid hemorrhage, *SDI* sociodemographic index, *UI* uncertainty interval, *CI* confidence interval

### Burden of SAH based on age and sex

In 2021, the incidence of SAH was predominantly observed in the age subgroups of 45 − 49 and 50 – 54 years. Among patients aged below 50 years, there was a higher number of incident cases in males compared to females. Conversely, among patients aged above 50 years, the number of incident cases was higher in females than males. The ASIRs for SAH showed slight differences between sexes among patients aged below 70 years; however, among those aged above 70 years, ASIR for SAH was significantly higher in males than females (Fig. S5a). Both sexes had the highest number of deaths within the age subgroup of 65 − 69 years. Mortality rate increased with age in females but only until the age subgroup of 90 − 94 years in males. Among patients younger than 65 years, there were more deaths in males compared to females; whereas among those older than 65 years, there were more deaths in females than males (Fig. S5b). The DALYs attributed to SAH were greatest within the age group of 55 − 59 years for both sexes. The rate of DALYs increased with age in females and peaked within the age subgroup of 90 − 94 years for males (Fig. S5c). Overall, the incidence, mortality, and DALYs exhibited the highest rates in the age subgroups of 50 − 54, 65 − 69, and 55 – 59 years, respectively. Therefore, the disease burden of SAH was predominantly concentrated within the age subgroup of 50 − 69 years.

### Attributable risk factors

**Fig. 3:**
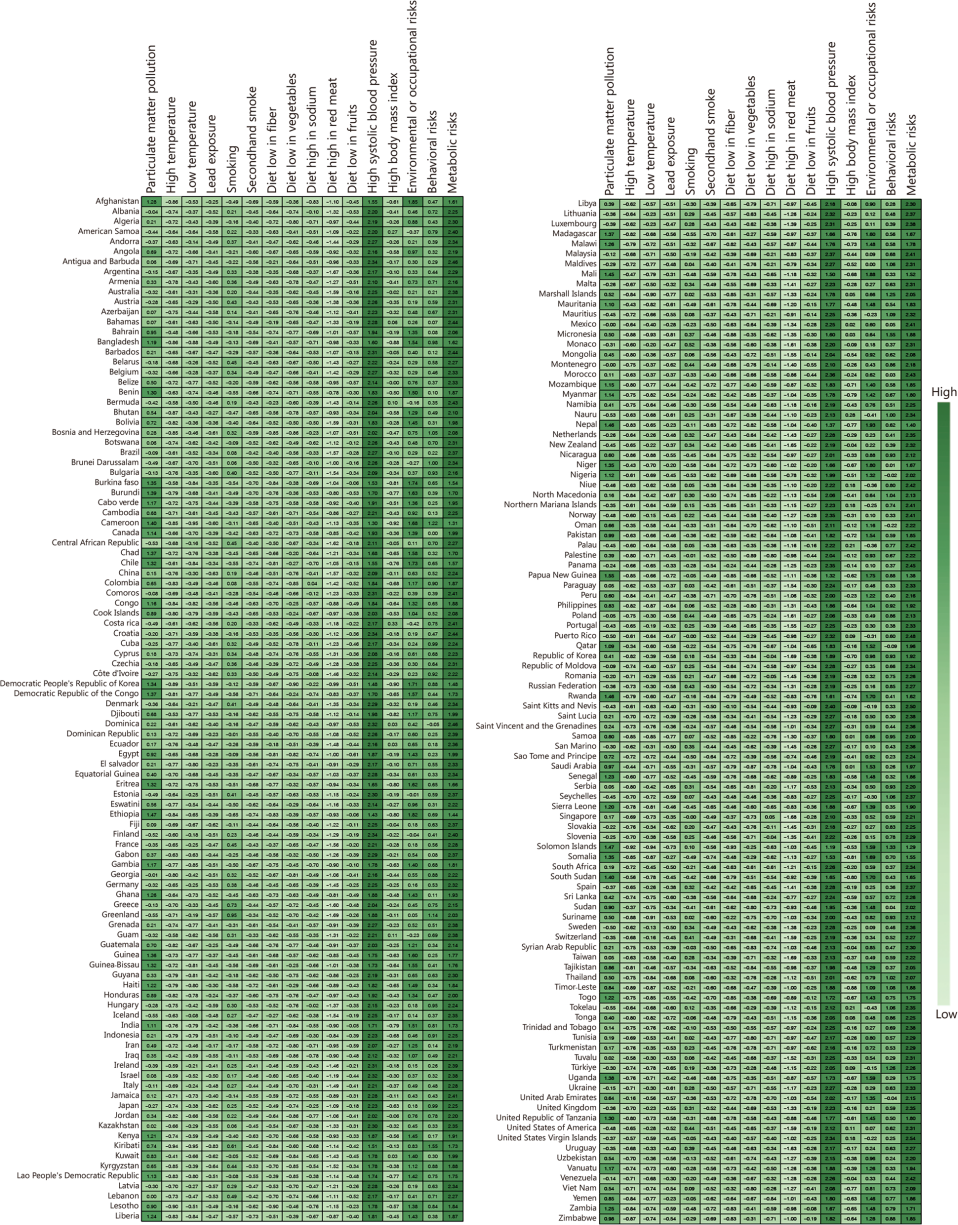
Attributable age-standardized DALY rates by SAH risk factors in 204 regions or countries in 2021. The darker the green color, the higher the value. SAH subarachnoid hemorrhage, DALY disability-adjusted life-year

The attributable DALYs caused by all risk factors of SAH in 2021 were 772.02 × 10^4^ (95% UI 651.89 × 10^4^ − 911.45 × 10^4^) (Additional file 1: Table S9). For male individuals, the leading risk factors were ambient particulate matter pollution, smoking, and high systolic blood pressure; whereas for female individuals, they were ambient particulate matter pollution, high systolic blood pressure, and a diet low in fruits (Additional file 1: Tables S9, S10). Moreover, both sexes of SAH patients with a diet high in red meat were found to have lower death and DALYs. Compared to environmental or occupational risks and behavioral risks, metabolic risks emerged as the primary cluster of risk factors for SAH (Fig. 3; Additional file 1: Tables S9, S10). High systolic blood pressure was identified as a major risk factor contributing to SAH-related disability and deaths (Fig. 3; Additional file 1: Tables S9, S10, Fig. S6).

## Discussion

The present study demonstrated significant changes in the burden and risk factors associated with SAH from 1990 to 2021, both globally and regionally. SAH is a prominent global disease that leads to death and disability [20]. While several studies have previously reported on the burden of stroke and the incidence and impact of SAH in specific regions [7, 8, 21–23], this study represents the first comprehensive analysis of SAH at both global and regional levels.

The incidence of SAH significantly increased from 1990 to 2021, accounting for a substantial proportion of strokes, which ranked as the second leading cause of death [8]. However, the ASIR for SAH decreased globally during this period. The observed change in the global incidence of SAH likely reflects population growth, aging demographics, and lifestyle changes [24]. In contrast to the incidence trend, the number of deaths and DALYs attributed to SAH declined over time. Additionally, both ASDR and ASMR showed a decrease. These changes may be due to advancements in medical care, particularly interventional therapies for cerebrovascular disease that have improved since 1990.

Endovascular treatment and surgery are effective strategies for preventing the rupture of intracranial aneurysms, which account for more than 70% of SAH and result in death or disability in up to 66% of all patients [20, 25]. Early detection and intervention of intracranial aneurysms can effectively reduce the burden of DALYs and mortality associated with SAH. To decrease death and disability rates, various methods have been developed to identify individuals at high risk of SAH, including advanced imaging examinations [26–29], questionnaires [30], genetic predictions [25], cerebrospinal fluid examination [31], application of artificial intelligence [32], and monitoring technologies [33, 34].

The ASIR, ASMR, and ASDR of SAH exhibited regional and national variations. In this study, the ASIR decreased in 21 regions, while the ASMR decreased in 18 regions. Additionally, DALYs decreased in 20 regions. Consistent with a previous study, all 21 regions demonstrated a downward trend in the ASIR for SAH [21]. Among the top 10 countries with the highest ASIR, 9 were Pacific Island nations including Solomon Islands, Kiribati, Marshall Islands, Federated States of Micronesia, Vanuatu, Tuvalu, Fiji, Nauru, and Japan. Several factors may have contributed to this phenomenon; however, it is speculative to determine their extent of influence. Firstly, these island countries might possess variant gene frequencies compared to continental nations or those with a large immigrant population. Secondly, the unique lifestyles and climates prevalent on these islands could also play a role. Furthermore, demographic changes within these populations may account for the high ASIR observed.

Among 21 regions, only Southern Sub-Saharan Africa, Central Asia, and Central Latin America experienced an increase in ASMR for SAH from 1990 to 2021. In 2021, island countries exhibited high ASMR and ASDR, with 8 out of the top 10 countries having the highest ASMR and all 10 having the highest ASDR being island nations. Japan had a high incidence rate of SAH, ranking 10th among 204 countries and regions; however, its mortality rate ranked 71 and DALYs ranked 58 among the same entities. The residents of these islands tend to consume food with higher salt content but fewer vegetables due to the limited availability of green leafy vegetables in island nations. Additionally, inadequate access to medical care contributes to lower awareness and control of hypertension in island countries. Regions and countries with high SDI exhibit lower incidences, mortalities, and DALYs associated with SAH due to improved socioeconomic conditions as well as better healthcare access and quality [35]. The increase in SAH incidence observed in middle SDI regions may be attributed to enhanced detection methods [33], as well as increased exposure to risk factors such as higher meat intake combined with lower vegetable consumption amidst remarkable living standards within this SDI range. The decrease in mortality and DALYs from 1990 − 2021 may be attributed to advancements in primary and secondary prevention, treatment of SAH and other neurologic and medical complications, as well as neurorehabilitation [36, 37]. In regions with low SDI, limited resources may result in a higher rate of rebleeding and unmanaged intracranial hypertension during the acute phase of the disease, leading to patients not surviving [38]. Additionally, the higher burden in low SDI regions could be due to inadequate quality, coverage, and content of national SAH guidelines [39]. Therefore, it is crucial for regions and countries with varying levels of social development to implement evidence- and national condition-based intervention strategies tailored specifically towards reducing the incidence, deaths, and DALYs associated with SAH.

Similar to the findings of a previous study [21], this study showed an association between increasing age and higher incidence rates of SAH. This could be attributed to the vulnerability of older patients due to their compromised physical condition and the presence of multiple chronic comorbidities. Furthermore, afflicted with a disease, older individuals experience a greater burden as their recovery capacity diminishes [40]. Moreover, advanced age renders individuals more susceptible to trauma, thereby heightening their inherent sensitivity to hemorrhage [41]. The overall global ASIR is observed to be higher in females compared to males; however, the ASMR and ASDR are lower in females than in males. This discrepancy may be linked to the correlation between frailty and SAH occurrence, with female individuals exhibiting a significantly higher prevalence of frailty than male individuals. Additionally, aneurysmal SAH is more prevalent among females. Nevertheless, regional comparisons fail to yield consistent results regarding ASIR, ASMR, and ASDR disparities between male and female patients due to various sex-specific risk factors for SAH such as lifestyle habits (e.g., smoking and alcohol consumption), physiologic differences, and disease awareness [42].

In this study, metabolic factors were found to be the primary attributable risk factors for SAH. High systolic blood pressure was identified as the most significant metabolic factor associated with SAH and was correlated with a poor prognosis. Moreover, males exhibited a higher susceptibility compared to females, which corroborated previous findings [43]. Elevated blood pressure may augment the risk of rebleeding in patients with SAH and induce hypoperfusion, leading to neurological deterioration caused by ischemia in hypoperfused brain regions [44]. With rapid economic development, there has been an increase in sodium and fat consumption. Coupled with unhealthy lifestyle behaviors, this has resulted in a rising incidence of hypertension, particularly among younger individuals [45, 46]. Therefore, reinforcing interventions targeting hypertension is crucial for preventing SAH. For young patients, emphasis should be placed on preventing hypertension and raising awareness about its implications. Dietary modifications and promoting healthy lifestyle choices should be implemented to prevent hypertension [47, 48]. In middle-aged and older patients who have already developed hypertension, effective anti-hypertensive strategies should be employed to enhance treatment outcomes and control blood pressure levels while reducing the risk of stroke.

Smoking is a significant risk factor for SAH in male patients, and the burden of SAH caused by smoking is larger in males than in females, potentially due to a higher prevalence of smoking among males. Outdoor particulate pollutants also play a crucial role in the development of SAH in male and female patients across all age groups, likely attributed to the escalating issue of air pollution resulting from increased industrialization. However, the impact of particulate matter pollution on disease burden is more pronounced in males as they are more susceptible to environmental risk factors compared to females. Therefore, enhancing air quality regulations and implementing measures to mitigate environmental pollution may effectively contribute to preventing SAH [49, 50]. Understanding these high-risk factors aids in identifying strategies for the prevention of SAH. It should be noted that China has made remarkable achievements in stroke prevention and acute treatment [50]. In addition to controlling risk factors for preventing SAH, early detection through prompt identification at an early stage followed by appropriate transportation and timely medical intervention is essential for reducing disabilities and mortality associated with SAH. Regular health check-ups are recommended for populations at high risk of developing SAH as it facilitates the detection and treatment of unruptured aneurysms before rupture occurs.

The severe complications resulting from SAH also contribute to increased mortality and disability, including delayed cerebral ischemia (DCI), delayed cerebral vasospasm, neurogenic pulmonary edema, sudden cardiac death, subdural effusion, and late hydrocephalus [51–53]. Delayed cerebral vasospasm is particularly recognized as a severe complication that significantly impacts the mortality rate and clinical outcomes of SAH [54]. DCI and vasospasm typically occur between 3 to 14 d after SAH onset and are the primary causes of morbidity in SAH patients, with approximately 16% developing vasospasm [55, 56]. It is crucial to emphasize the diagnosis and implementation of rescue therapies for DCI and vasospasm in order to improve the prognosis of SAH.

This study represents the first comprehensive analysis of the latest global and regional burdens associated with SAH. While previous studies have addressed stroke burden to some extent, they only partially covered the impact of SAH [8, 9, 57, 58]. Based on up-to-date epidemiological data spanning from 1990 to 2021 across 204 countries and territories worldwide, this study reveals disease burden trends according to geographic location, SDI, age groups, and sex. Notably for the first time ever in research literature on this topic at both national/regional levels as well as globally. Furthermore, this study highlights that metabolic risk factors such as high systolic blood pressure have a greater influence compared to environmental or occupational risk factors like ambient particulate matter pollution or behavioral risk factors such as diets low in fruits or red meat. Therefore, major emphasis should be placed on addressing metabolic risk factors which constitute the main cluster contributing to the development of SAH.

However, this study has certain limitations. Firstly, the estimates rely on the available data sources, and the accuracy of the GBD data depends on the quality of the existing data in each country. The reporting and predictability of SAH data for 204 countries may be inadequate, leading to potential inaccuracies. Secondly, it should be noted that the GBD data sources do not encompass all populations or regions; therefore, the findings only represent the general overview of specific regions. Thirdly, there is a possibility that DALYs might have been underestimated due to assuming independence between YLL and YLD in this current study. Lastly, it is important to acknowledge that the risk factors for SAH in GBD 2021 are limited and do not include the full spectrum of disease etiologies.

## Conclusions

The present study presents crucial data on SAH that have implications for global public health. Despite a decline in the burden of SAH over recent decades, it remains a significant challenge to global public health. To further alleviate its impact on society, proactive intervention strategies at both administrative and academic levels should be implemented based on the geographical distribution and epidemiological characteristics of SAH.

### Supplementary Information

**Additional file 1: Table S1** Global Burden of Disease Study risk hierarchy with levels. **Table S2** Definition of all risk factors. **Table S3** Regional incidence, mortality, and DALYs of SAH in 2021. **Table S4** ASIR, ASMR, and ASDR for subarachnoid hemorrhage (SAH) in 21 regions in 2021. **Table S5** EAPC of ASIR, ASMR, and ASDR for subarachnoid hemorrhage (SAH) in 21 regions from 1990 to 2021. **Table S6** Incidence, mortality, and DALYs for subarachnoid hemorrhage (SAH) in 204 countries in 2021. **Table S7** ASIR, ASMR, and ASDR for subarachnoid hemorrhage (SAH) in 204 countries in 2021. **Table S8** EAPC of ASIR, ASDR for subarachnoid hemorrhage (SAH) in 204 countries and territories from 1990 to 2021. **Table S9** Attributable DALYs and age-standardized DALY rate by SAH risk factors in 2021. **Table S10** Attributable deaths and age-standardized deaths rate by SAH risk factors in 2021. **Fig. S1** Flowcharting of the analysis process. **Fig. S2** The age-standardized rates of SAH during 1990 − 2021 by sex. **Fig. S3** ASMR and ASDR of SAH for 21 regions and 204 countries and territories by SDI. **Fig. S4** ASMR and ASDR of SAH for 204 countries and territories by SDI in 2021. **Fig. S5** The global incidence, number of deaths, and DALYs due to SAH by age and sex. **Fig. S6** Attributable age-standardized death rate by SAH risk factors in 204 regions or countries in 2021.

## Data Availability

The datasets generated and/or analyzed during the current study are available in the GBD 2021. Publicly available datasets were analyzed in the current study. The data can be found here: http://ghdx.healthdata.org/gbd-results-tool. The analyzed data will be shared upon reasonable request to the corresponding author.

## Abbreviations

ASDR: Age-standardized disability-adjusted life-year rate
ASIR: Age-standardized incidence rate
ASMR: Age-standardized mortality rate
ASR: Age-standardized rate
CI: Confidence interval
DALYs: Disability-adjusted life-years
DCI: Delayed cerebral ischemia
EAPC: Estimated annual percentage change
GBD: Global Burden of Disease Study
ICD: International Classification of Disease
SAH: Subarachnoid hemorrhage
SDI: Sociodemographic index
UI: Uncertainty interval
YLD: Years lived with disability
YLL: Years of life lost
