# Supplementary material for: Epidemiological trends of subarachnoid hemorrhage at global, regional, and national level: a trend analysis study from 1990 to 2021
Source: Mil Med Res. 2024 Jul 11;11:46. doi: 10.1186/s40779-024-00551-6 (PMC11241879; doi:10.1186/s40779-024-00551-6)
Supplement: Supplementary file 1 — Additional file 1: Table S1 Global Burden of Disease Study risk hierarchy with levels. Table S2 Definition of all risk factors. Table S3 Regional incidence, mortality, and DALYs of SAH in 2021. Table S4 ASIR, ASMR, and ASDR for subarachnoid hemorrhage (SAH) in 21 regions in 2021. Table S5 EAPC of ASIR, ASMR, and ASDR for subarachnoid hemorrhage (SAH) in 21 regions from 1990 to 2021. Table S6 Incidence, mortality, and DALYs for subarachnoid hemorrhage (SAH) in 204 countries in 2021. Table S7 ASIR, ASMR, and ASDR for subarachnoid hemorrhage (SAH) in 204 countries in 2021. Table S8 EAPC of ASIR, ASDR for subarachnoid hemorrhage (SAH) in 204 countries and territories from 1990 to 2021. Table S9 Attributable DALYs and age-standardized DALY rate by SAH risk factors in 2021. Table S10 Attributable deaths and age-standardized deaths rate by SAH risk factors in 2021. Fig. S1 Flowcharting of the analysis process. Fig. S2 The age-standardized rates of SAH during 1990 − 2021 by sex. Fig. S3 ASMR and ASDR of SAH for 21 regions and 204 countries and territories by SDI. Fig. S4 ASMR and ASDR of SAH for 204 countries and territories by SDI in 2021. Fig. S5 The global incidence, number of deaths, and DALYs due to SAH by age and sex. Fig. S6 Attributable age-standardized death rate by SAH risk factors in 204 regions or countries in 2021. [file 40779_2024_551_MOESM1_ESM.pdf]

Table S1 Global Burden of Disease Study risk hierarchy with levels

**Risk**	**Level**
All risk factors	0
Environmental or occupational risks	1
Particulate matter pollution	3
High temperature	3
Low temperature	3
Lead exposure	3
Behavioral risks	1
Tobacco	2
Smoking	3
Secondhand smoke exposure	3
Dietary risks	2
Diet low in fruits	3
Diet low in vegetables	3
Diet high in red meat	3
Diet low in fiber	3
Diet high in sodium	3
Metabolic risks	1
High systolic blood pressure	2
High body mass index	2

Table S2 Definition of all risk factors

All risk factor	Definition
Environmental or occupational risks	
Particulate matter pollution	Including ambient particulate matter pollution and household air pollution from solid fuels. Exposure to ambient particulate matter pollution is defined as the population-weighted annual average mass concentration of particles with an aerodynamic diameter of less than 2.5 micrometers (PM2.5) in a cubic meter of air. Exposure to household air pollution from solid fuels is estimated from both the proportion of individuals using solid cooking fuels and the level of PM2.5 air pollution exposure for these individuals. Solid fuels in our analysis include coal, wood, charcoal, dung, and agricultural residues
High temperature	Theoretical minimum risk exposure level (TMREL) for temperature is associated with the lowest overall mortality attributable to the risk, in a given location and year. Given varying exposure-response curves for different mean annual temperature zones, as well as spatially and temporally varying cause compositions, the Global Burden of Disease Study (GBD) estimates TMRELs by year and location and does not use a globally uniform TMREL. High temperature (heat) exposure is defined as exposure to temperatures warmer than this TMREL
Low temperature	Low temperature (cold) is defined as temperatures colder than the TMREL
Lead exposure	Exposure to lead is defined in two different ways according to the currently known pathways of attributable health loss. Acute lead exposure, measured as micrograms of lead per deciliter of blood (μg/dl), is associated with IQ loss in children. Chronic lead exposure, measured as micrograms of lead per gram of bone (μg/g), is associated with increased systolic blood pressure and cardiovascular diseases
Behavioral risks	
Tobacco	
Smoking	Including current smoking of any tobacco product and former smoking of any tobacco product

All risk factor	Definition
Secondhand smoke exposure	Secondhand smoke exposure is defined as current exposure to secondhand tobacco smoke at home, at work, or in other public places. Only non-smokers are considered to be exposed to secondhand smoke. Non-smokers are defined as all persons who are not daily smokers
Dietary risk	
Diet low in fiber	Average daily consumption (g/d) of less than 21 − 22 g of fiber from all sources including fruits, vegetables, grains, legumes, and pulses
Diet low in vegetables	Average daily consumption (g/d) of less than 280 − 320 g of vegetables, including fresh, frozen, cooked, canned, or dried vegetables and excluding legumes and salted or pickled vegetables, juices, nuts and seeds, and starchy vegetables such as potatoes or corn
Diet high in sodium	Average 24-hour urinary sodium excretion (g/d) greater than 1 − 5 g
Diet high in red meat	Any intake (g/d) of red meat including beef, pork, lamb, and goat but excluding poultry, fish, eggs, and all processed meats
Diet low in fruits	Diet low in fruits of fruits including fresh, frozen, cooked, canned, or dried fruit, excluding fruit juices and salted or pickled fruits
Metabolic risks	
High systolic blood pressure	Brachial systolic blood pressure in mmHg. A TMREL of ≥ 110 to 115 mmHg, which is the level of exposure that minimizes risk at the population level
High body mass index (BMI)	High BMI for adults (age > 20 years) is defined as BMI greater than 20 to 25 kg/m^2<br>High BMI for children (ages 1 – 19 years) is defined as being overweight or obese based on International Obesity Task Force standards

Table S3 Regional incidence, deaths, and DALYs of subarachnoid hemorrhage (SAH) in 2021

Location	Incidence/1000 (95% UI)			Deaths/1000 (95% UI)			DALYs/1000 (95% UI)		
	**Both**	**Male**	**Female**	**Both**	**Male**	**Female**	**Both**	**Male**	**Female**
Andean Latin America	7.84<br>(7.00 − 8.78)	3.45<br>(3.07 − 3.90)	4.39<br>(3.90 − 4.93)	3.64<br>(2.95 − 4.43)	1.51<br>(1.19 − 1.88)	2.13<br>(1.71 − 2.65)	126.53<br>(105.43 − 151.58)	56.34<br>(45.28 − 68.83)	70.19<br>(57.77 − 85.59)
Australasia	2.63<br>(2.33 − 2.99)	1.06<br>(0.93 − 1.20)	1.57<br>(1.38 − 1.80)	1.32<br>(1.16 − 1.43)	0.56<br>(0.51 − 0.62)	0.76<br>(0.64 − 0.84)	33.26<br>(30.72 − 35.75)	14.40<br>(13.35 − 15.51)	18.86<br>(17.10 − 20.63)
Caribbean	5.33<br>(4.78 − 6.04)	2.17<br>(1.93 − 2.47)	3.15<br>(2.82 − 3.58)	2.63<br>(2.04 − 3.28)	1.08<br>(0.79 − 1.47)	1.54<br>(1.12 − 1.96)	96.28<br>(73.56 − 118.61)	39.34<br>(28.85 − 51.25)	56.93<br>(38.73 − 72.70)
Central Asia	7.81<br>(6.96 − 8.73)	4.16<br>(3.67 − 4.65)	3.66<br>(3.25 − 4.09)	4.01<br>(3.65 − 4.45)	2.15<br>(1.92 − 2.42)	1.86<br>(1.66 − 2.07)	125.92<br>(112.90 − 139.77)	72.13<br>(64.53 − 81.10)	53.79<br>(47.98 − 60.02)
Central Europe	12.71<br>(11.52 − 13.98)	5.76<br>(5.18 − 6.42)	6.96<br>(6.35 − 7.63)	7.01<br>(6.43 − 7.56)	3.07<br>(2.82 − 3.34)	3.94<br>(3.56 − 4.30)	189.36<br>(174.64 − 203.80)	89.78<br>(82.17 − 97.81)	99.57<br>(91.12 − 108.47)
Central Latin America	28.47<br>(25.35 − 32.27)	11.56<br>(10.24 − 13.13)	16.91<br>(15.12 − 19.24)	11.95<br>(10.60 − 13.37)	5.11<br>(4.47 − 5.80)	6.83<br>(5.97 − 7.78)	392.19<br>(350.31 − 440.36)	174.96<br>(154.32 − 198.84)	217.23<br>(190.86 − 247.26)
Central Sub-Saharan Africa	6.07<br>(5.22 − 7.07)	3.43<br>(2.91 − 4.02)	2.64<br>(2.25 − 3.09)	1.85<br>(0.77 − 5.03)	1.06<br>(0.26 − 4.17)	0.78<br>(0.44 − 1.87)	74.84<br>(37.07 − 182.35)	44.13<br>(14.95 − 145.97)	30.71<br>(19.42 − 64.51)
East Asia	151.82<br>(131.56 − 176.50)	82.14<br>(70.43 − 96.92)	69.68<br>(60.57 − 80.45)	95.18<br>(70.28 − 119.16)	50.64<br>(30.87 − 70.57)	44.54<br>(31.32 − 59.61)	2396.95<br>(1840.65 − 2934.11)	1307.40<br>(847.93 − 1754.93)	1089.56<br>(810.77 − 1419.54)
Eastern Europe	27.74<br>(24.53 − 31.83)	13.40<br>(11.73 − 15.51)	14.34<br>(12.69 − 16.37)	18.20<br>(16.68 − 19.69)	8.18<br>(7.35 − 8.97)	10.02<br>(8.90 − 11.14)	494.66<br>(457.05 − 532.54)	256.52<br>(231.13 − 281.82)	238.13<br>(212.91 − 263.19)

Eastern Sub-Saharan Africa	18.58 (15.96 − 21.45)	10.93 (9.38 − 12.64)	7.65 (6.52 − 8.89)	5.58 (2.11 − 15.46)	3.62 (0.94 − 13.16)	1.96 (1.06 − 5.13)	234.78 (105.03 − 589.27)	152.97 (50.76 − 499.79)	81.81 (49.95 − 182.04)
High-income Asia Pacific	46.62 (41.13 − 53.65)	17.29 (15.06 − 20.07)	29.33 (25.57 − 33.76)	17.74 (14.89 − 19.54)	6.69 (6.19 − 7.08)	11.05 (8.63 − 12.55)	485.22 (434.13 − 531.78)	211.94 (197.05 − 228.01)	273.28 (235.95 − 305.76)
High-income North America	33.65 (29.72 − 38.72)	13.84 (12.16 − 15.87)	19.81 (17.34 − 22.99)	19.71 (17.78 − 20.83)	8.15 (7.57 − 8.56)	11.55 (10.12 − 12.38)	525.52 (495.26 − 554.72)	221.87 (210.94 − 233.29)	303.64 (281.13 − 323.38)
North Africa and the Middle East	32.39 (28.11 − 36.31)	15.73 (13.71 − 17.73)	16.65 (14.35 − 18.73)	12.26 (9.70− 15.97)	6.20 (4.46 − 9.03)	6.06 (4.51 − 8.06)	433.90 (351.65 − 547.03)	218.84 (165.02 − 305.76)	215.07 (168 − 279.83)
Oceania	1.27 (1.12 − 1.42)	0.64 (0.57 − 0.73)	0.62 (0.56 − 0.70)	0.70 (0.50 − 0.96)	0.34 (0.20 − 0.57)	0.36 (0.25 − 0.50)	31.04 (23.60 − 40.11)	15.80 (10.33 − 24.52)	15.24 (10.95 − 20.24)
South Asia	140.53 (120.48 − 162.71)	75.53 (64.40 − 87.75)	65.01 (56.02 − 75.42)	67.62 (48.40 − 93.44)	37.65 (18.61 − 61.30)	29.97 (22.66 − 41.56)	2362.78 (1772.14 − 3126.99)	1334.95 (738.77 − 2034.90)	1027.82 (797.12 − 1375.85)
Southeast Asia	74.81 (65.47 − 85.58)	36.53 (31.77 − 41.88)	38.28 (33.65 − 44.15)	37.58 (31.54 − 51.34)	18.06 (13.81 − 26.90)	19.52 (15.20 − 28.72)	1279.43 (1094.27 − 1636.99)	658.43 (521.73 − 918.62)	621.01 (499.52 − 832.12)
Southern Latin America	8.42 (7.55 − 9.48)	3.29 (2.95 − 3.73)	5.12 (4.55 − 5.84)	3.52 (3.25 − 3.75)	1.53 (1.41 − 1.64)	1.99 (1.81 − 2.14)	108.16 (101.03 − 114.85)	48.41 (44.85 − 51.99)	59.75 (55.28 − 64.18)
Southern Sub-Saharan Africa	3.59 (3.11 − 4.14)	1.73 (1.49 − 1.98)	1.86 (1.61 − 2.19)	1.22 (1.01 − 1.57)	0.59 (0.45 − 0.84)	0.63 (0.51 − 0.78)	47.01 (39.29 − 58.93)	24.07 (18.84 − 33.54)	22.94 (18.89 − 27.48)
Tropical Latin America	26. 00 (22.46 − 30.29)	10.22 (8.80 − 11.87)	15.78 (13.58 − 18.43)	14.03 (13.23 − 14.63)	5.33 (5.06 − 5.61)	8.71 (8.01 − 9.18)	460.76 (440.24 − 479.16)	178.07 (169.66 − 186.85)	282.69 (267.25 − 296.22)
Western Europe	43.95 (39.40 − 49.55)	18.63 (16.41 − 20.98)	25.32 (22.68 − 28.70)	21.91 (19.17 − 23.40)	9.27 (8.57 − 9.74)	12.65 (10.70 − 13.74)	511.25 (473.46 − 544.42)	226.36 (212.89 − 240.12)	284.89 (258.23 − 305.67)

Western Sub-Saharan Africa	17.26 (14.76 − 19.80)	9.35 (8.02 − 10.73)	7.91 (6.70 − 9.10)	5.15 (2.60 − 11.59)	2.94 (0.99 − 9.23)	2.21 (1.44 − 4.61)	232.05 (137.19 − 464.76)	136.74 (62.36 − 370.48)	95.31 (67.70 − 171.76)

DALYs disability-adjusted life-years, *UI* uncertainty interval

Table S4 ASIR, ASMR, and ASDR for subarachnoid hemorrhage (SAH) in 21 regions in 2021

Location	ASIR/100,000 persons (95% UI)			ASMR/100,000 persons (95% UI)			ASDR /100,000 persons (95% UI)		
	Both	Male	Female	Both	Male	Female	Both	Male	Female
Andean Latin America	12.29 (11.04 − 13.73)	11.02 (9.82 − 12.43)	13.48 (12.02 − 15.11)	6.01 (4.87 − 7.29)	5.16 (4.05 − 6.38)	6.77 (5.46 − 8.40)	199.49 (166.06 − 238.99)	179.70 (144.13 − 220.19)	217.34 (179.04 − 264.63)
Australasia	6.06 (5.34 − 6.86)	5.15 (4.51 − 5.83)	6.94 (6.04 − 7.94)	2.48 (2.22 − 2.68)	2.37 (2.16 − 2.58)	2.59 (2.27 − 2.83)	73.27 (68.07 − 78.65)	67.75 (63.05 − 72.85)	78.37 (71.78 − 85.49)
Caribbean	10.40 (9.34 − 11.71)	8.82 (7.86 − 9.97)	11.93 (10.65 − 13.47)	5.02 (3.88 − 6.25)	4.37 (3.17 − 5.88)	5.64 (3.99 − 7.19)	191.37 (146.14 − 234.04)	160.79 (117.34 − 206.86)	220.85 (146.49 − 283.13)
Central Asia	8.97 (8.10 − 9.98)	10.20 (9.17 − 11.31)	7.86 (7.04 − 8.77)	5.20 (4.73 − 5.76)	6.30 (5.68 − 7.06)	4.32 (3.86 − 4.81)	143.22 (129.04 − 158.53)	176.40 (158.86 − 197.43)	114.41 (102.27 − 127.13)
Central Europe	7.27 (6.55 − 8.02)	7.46 (6.67 − 8.26)	7.15 (6.39 − 7.92)	3.39 (3.11 − 3.65)	3.54 (3.25 − 3.85)	3.24 (2.93 − 3.55)	106.16 (97.77 − 114.30)	110.43 (101.11 − 120.26)	101.77 (93.08 − 110.76)
Central Latin America	11.07 (9.92 − 12.51)	9.59 (8.50 − 10.84)	12.40 (11.13 − 14.08)	4.76 (4.22 − 5.31)	4.40 (3.85 − 4.98)	5.05 (4.41 − 5.74)	151.45 (135.49 − 170.06)	143 (126.16 − 162.59)	158.25 (139.19 − 180.17)
Central Sub-Saharan Africa	7.29 (6.26 − 8.51)	8.58 (7.36 − 10.06)	6.22 (5.32 − 7.35)	3.15 (1.24 − 8.95)	3.97 (0.87 − 16.73)	2.55 (1.38 − 6.28)	99.08 (47.49 − 248.36)	120.36 (37.58 − 445.13)	80.51 (49.44 − 175.09)
East Asia	7.89 (6.94 − 9.03)	8.84 (7.73 − 10.20)	7.05 (6.16 − 8.03)	4.71 (3.49 − 5.87)	5.62 (3.37 − 7.77)	4.03 (2.84 − 5.41)	116.37 (89.68 − 141.75)	133.76 (86.14 − 178.88)	100.87 (76.37 − 130.67)
Eastern Europe	9.59 (8.42 − 10.87)	11.19 (9.80 − 12.78)	8.22 (7.22 − 9.36)	5.49 (5.03 − 5.93)	6.54 (5.90 − 7.16)	4.62 (4.12 − 5.14)	162.91 (150.36 − 175.08)	200.83 (181.05 − 220.18)	130.59 (116 − 144.77)

Eastern Sub-Saharan Africa	7.25 (6.25 − 8.62)	8.77 (7.54 − 10.31)	5.89 (5.05 − 6.94)	2.92 (1.10 − 8.24)	3.95 (0.98 − 15.05)	2.03 (1.07 − 5.46)	96.66 (42.93 − 245.35)	127.42 (41.61 − 429.22)	68.25 (41.18 − 161.60)
High-income Asia Pacific	14.09 (12.30 − 16.39)	11.93 (10.22 − 13.76)	15.95 (13.81 − 18.66)	3.90 (3.48 − 4.20)	3.73 (3.49 − 3.96)	3.88 (3.28 − 4.29)	135.70 (124.59 − 146.65)	136.84 (128.08 − 146.82)	131.39 (118.46 − 144.12)
High-income North America	6.37 (5.56 − 7.31)	5.62 (4.94 − 6.39)	7.15 (6.21 − 8.28)	3.15 (2.87 − 3.31)	2.97 (2.77 − 3.13)	3.30 (2.98 − 3.50)	97.81 (92.56 − 103.14)	89.40 (84.76 − 94)	105.46 (99 − 112.12)
North Africa and the Middle East	5.96 (5.27 − 6.66)	5.75 (5.10 − 6.44)	6.22 (5.50 − 7.01)	2.85 (2.28 − 3.69)	2.84 (1.99 − 4.13)	2.86 (2.15 − 3.77)	82.79 (67.60 − 104.61)	81.08 (60.92 − 113.57)	84.64 (65.68 − 109.65)
Oceania	12.52 (11.25 − 13.97)	11.55 (10.35 − 13.11)	13.50 (12.10 − 15.08)	8.61 (6.03 − 11.95)	7.23 (4.18 − 12.80)	9.96 (6.78 − 14.27)	285.62 (209.42 − 379.65)	269.77 (173 − 436.66)	302.38 (215.65 − 404.09)
South Asia	8.44 (7.33 − 9.75)	9.12 (7.90 − 10.50)	7.80 (6.74 − 9.05)	4.47 (3.14 − 6.23)	5.09 (2.47 − 8.35)	3.90 (2.94 − 5.49)	142.60 (106.78 − 189.60)	161.16 (87.73 − 248.39)	124.34 (96.59 − 166.87)
Southeast Asia	10.89 (9.66 − 12.36)	11.12 (9.82 − 12.61)	10.70 (9.47 − 12.16)	6.02 (5.02 − 8.42)	6.00 (4.56 − 9.23)	5.91 (4.61 − 8.95)	182.03 (155.77 − 235.86)	190.48 (151.77 − 268.36)	171.75 (138.38 − 232.82)
Southern Latin America	10.65 (9.51 − 11.93)	9.01 (8.10 − 10.17)	12.17 (10.73 − 13.85)	4.13 (3.82 − 4.39)	4.12 (3.80 − 4.43)	4.17 (3.81 − 4.48)	134.19 (125.36 − 142.61)	129.99 (120.33 − 139.57)	138.22 (128.30 − 148.36)
Southern Sub-Saharan Africa	5.26 (4.59 − 6.10)	5.53 (4.83 − 6.36)	5.07 (4.37 − 5.91)	2.05 (1.70 − 2.62)	2.24 (1.72 − 3.16)	1.89 (1.53 − 2.34)	69.35 (58.45 − 86.11)	76.17 (60.43 − 104.79)	63.05 (52.41 − 75.64)
Tropical Latin America	10.21 (8.88 − 11.79)	8.66 (7.50 – 10.00)	11.66 (10.10 − 13.57)	5.45 (5.12 − 5.68)	4.61 (4.35 − 4.87)	6.20 (5.71 − 6.53)	177.53 (169.49 − 184.75)	147.03 (140.10 − 154.33)	204.92 (194.01 − 214.57)
Western Europe	6.31 (5.57 − 7.13)	5.81 (5.15 − 6.54)	6.81 (5.94 − 7.75)	2.28 (2.07 − 2.41)	2.28 (2.14 − 2.39)	2.29 (2.05 − 2.45)	67.75 (63.56 − 71.94)	65.66 (62.01 − 69.54)	69.78 (64.68 − 74.36)

Western Sub-Saharan Africa	5.00 (4.34 − 5.84)	5.64 (4.87 − 6.54)	4.42 (3.79 − 5.16)	2.22 (1.12 − 5.09)	2.51 (0.76 − 8.51)	1.95 (1.26 − 4.38)	77.81 (44.39 − 161.92)	91.73 (37.43 − 266.26)	65.08 (45.87 − 124.61)

ASIR age-standardized incidence rate, *ASMR* age-standardized mortality rate, *ASDR* age-standardized disability-adjusted life-year rate, *UI* uncertainty interval

Table S5 EAPC of ASIR, ASMR, and ASDR for subarachnoid hemorrhage (SAH) in 21 regions from 1990 to 2021

Location	EAPC of ASIR (95% CI)			EAPC of ASMR (95% CI)			EAPC of ASDR (95% CI)		
	Both	Male	Female	Both	Male	Female	Both	Male	Female
Andean Latin America	-0.99 (-1.05 to -0.93)	-0.89 (-0.96 to -0.82)	-1.07 (-1.13 to -1.01)	-1.03 (-1.18 to -0.88)	-1.10 (-1.25 to -0.96)	-0.98 (-1.15 to -0.82)	-1.32 (-1.45 to -1.19)	-1.33 (-1.44 to -1.21)	-1.32 (-1.47 to -1.16)
Australasia	-0.80 (-0.85 to -0.76)	-0.60 (-0.65 to -0.54)	-0.92 (-0.96 to -0.87)	-1.96 (-2.06 to -1.85)	-1.47 (-1.63 to -1.31)	-2.24 (-2.34 to -2.15)	-2.16 (-2.24 to -2.08)	-1.91 (-2.04 to -1.79)	-2.32 (-2.40 to -2.25)
Caribbean	-0.43 (-0.50 to -0.37)	-0.34 (-0.40 to -0.27)	-0.50 (-0.56 to -0.43)	-0.75 (-0.87 to -0.64)	-0.76 (-0.91 to -0.61)	-0.75 (-0.84 to -0.65)	-0.67 (-0.80 to -0.53)	-0.72 (-0.89 to -0.55)	-0.63 (-0.74 to -0.51)
Central Asia	-0.05 (-0.09 to -0.02)	0.03 (-0.01 to 0.08)	-0.19 (-0.21 to -0.16)	0.54 (0.33 − 0.75)	0.55 (0.34 − 0.77)	0.41 (0.21 − 0.61)	-0.05 (-0.21 to 0.12)	0.15 (-0.04 to 0.33)	-0.34 (-0.50 to -0.18)
Central Europe	-1.14 (-1.23 to -1.04)	-0.95 (-1.03 to -0.87)	-1.30 (-1.41 to -1.19)	-1.65 (-1.91 to -1.38)	-1.43 (-1.65 to -1.22)	-1.84 (-2.14 to -1.54)	-2.00 (-2.23 to -1.76)	-1.79 (-1.99 to -1.59)	-2.19 (-2.45 to -1.92)
Central Latin America	-0.28 (-0.30 to -0.25)	-0.12 (-0.14 to -0.10)	-0.41 (-0.44 to -0.38)	0.40 (0.26 − 0.54)	0.76 (0.63 − 0.90)	0.11 (-0.04 to 0.26)	-0.02 (-0.10 to 0.06)	0.37 (0.29 to 0.45)	-0.33 (-0.42 to -0.25)
Central Sub-Saharan Africa	-0.25 (-0.29 to -0.22)	-0.20 (-0.24 to -0.17)	-0.26 (-0.31 to -0.22)	-0.48 (-0.56 to -0.41)	-0.37 (-0.47 to -0.26)	-0.50 (-0.58 to -0.42)	-0.57 (-0.63 to -0.51)	-0.47 (-0.55 to -0.39)	-0.69 (-0.76 to -0.62)
East Asia	-3.60 (-3.97 to -3.22)	-3.62 (-4.04 to -3.19)	-3.60 (-3.92 to -3.28)	-6.56 (-7.21 to -5.91)	-6.01 (-6.56 to -5.45)	-7.13 (-7.87 to -6.39)	-6.17 (-6.76 to -5.57)	-5.71 (-6.24 to -5.18)	-6.70 (-7.36 to -6.03)
Eastern Europe	-0.27 (-0.52 to -0.02)	-0.24 (-0.46 to -0.03)	-0.36 (-0.63 to -0.09)	-0.72 (-1.65 to 0.22)	-0.77 (-1.62 to 0.09)	-0.77 (-1.76 to 0.23)	-0.74 (-1.46 to -0.02)	-0.71 (-1.42 to -0.01)	-0.82 (-1.56 to -0.08)

Eastern Sub-Saharan Africa	-0.90 (-0.97 to -0.83)	-0.77 (-0.84 to -0.70)	-1.02 (-1.10 to -0.95)	-1.55 (-1.61 to -1.50)	-1.62 (-1.69 to -1.55)	-1.29 (-1.36 to -1.22)	-1.44 (-1.50 to -1.39)	-1.47 (-1.53 to -1.41)	-1.32 (-1.39 to -1.25)
High-income Asia Pacific	-0.73 (-0.80 to -0.66)	-0.54 (-0.60 to -0.48)	-0.77 (-0.86 to -0.69)	-2.90 (-3.00 to -2.80)	-2.44 (-2.55 to -2.32)	-3.21 (-3.31 to -3.11)	-2.54 (-2.60 to -2.47)	-2.25 (-2.33 to -2.18)	-2.75 (-2.83 to -2.68)
High-income North America	-0.55 (-0.72 to -0.37)	-0.17 (-0.34 to 0.01)	-0.77 (-0.94 to -0.60)	-0.58 (-0.73 to -0.43)	0.10 (-0.09 to 0.29)	-0.97 (-1.12 to -0.82)	-1.01 (-1.11 to -0.92)	-0.53 (-0.62 to -0.44)	-1.32 (-1.43 to -1.21)
North Africa and the Middle East	-1.54 (-1.63 to -1.44)	-1.59 (-1.69 to -1.48)	-1.49 (-1.59 to -1.39)	-2.61 (-2.75 to -2.48)	-3.08 (-3.22 to -2.95)	-2.11 (-2.25 to -1.97)	-2.90 (-3.01 to -2.79)	-3.22 (-3.34 to -3.09)	-2.56 (-2.66 to -2.46)
Oceania	-0.70 (-0.74 to -0.65)	-0.56 (-0.60 to -0.51)	-0.79 (-0.84 to -0.73)	-1.21 (-1.25 to -1.18)	-1.13 (-1.20 to -1.06)	-1.23 (-1.27 to -1.20)	-1.05 (-1.08 to -1.02)	-0.97 (-1.02 to -0.92)	-1.10 (-1.16 to -1.04)
South Asia	-1.12 (-1.23 to -1.02)	-1.00 (-1.10 to -0.91)	-1.22 (-1.35 to -1.10)	-1.38 (-1.43 to -1.33)	-1.50 (-1.56 to -1.45)	-1.10 (-1.19 to -1.01)	-1.35 (-1.39 to -1.30)	-1.45 (-1.50 to -1.39)	-1.13 (-1.22 to -1.05)
Southeast Asia	-0.89 (-0.96 to -0.82)	-0.81 (-0.87 to -0.75)	-0.98 (-1.06 to -0.90)	-1.46 (-1.54 to -1.37)	-1.42 (-1.49 to -1.36)	-1.52 (-1.63 to -1.41)	-1.39 (-1.46 to -1.32)	-1.29 (-1.35 to -1.22)	-1.53 (-1.61 to -1.45)
Southern Latin America	-1.52 (-1.64 to -1.40)	-1.44 (-1.57 to -1.32)	-1.56 (-1.68 to -1.44)	-2.68 (-2.90 to -2.46)	-2.79 (-3.09 to -2.48)	-2.57 (-2.74 to -2.40)	-2.85 (-3.02 to -2.67)	-3.03 (-3.27 to -2.78)	-2.68 (-2.82 to -2.55)
Southern Sub-Saharan Africa	-0.41 (-0.52 to -0.30)	-0.39 (-0.51 to -0.28)	-0.44 (-0.54 to -0.34)	0.67 (0.28 − 1.05)	0.22 (-0.16 to 0.61)	1.11 (0.68 − 1.54)	0.50 (0.16 − 0.84)	0.20 (-0.15 to 0.54)	0.79 (0.40 − 1.18)
Tropical Latin America	-1.36 (-1.51 to -1.22)	-1.25 (-1.37 to -1.14)	-1.45 (-1.62 to -1.28)	-1.06 (-1.26 to -0.85)	-1.02 (-1.18 to -0.87)	-1.09 (-1.32 to -0.85)	-1.64 (-1.82 to -1.47)	-1.69 (-1.82 to -1.57)	-1.62 (-1.83 to -1.42)
Western Europe	-0.90 (-0.95 to -0.85)	-0.76 (-0.79 to -0.72)	-0.99 (-1.05 to -0.92)	-1.51 (-1.71 to -1.31)	-0.97 (-1.20 to -0.75)	-1.80 (-1.99 to -1.60)	-1.96 (-2.11 to -1.81)	-1.71 (-1.86 to -1.55)	-2.12 (-2.27 to -1.97)

Western Sub-Saharan Africa	-0.81 (-0.89 to -0.73)	-0.84 (-0.93 to -0.76)	-0.74 (-0.81 to -0.67)	-1.33 (-1.43 to -1.22)	-1.43 (-1.55 to -1.30)	-1.08 (-1.16 to -1.00)	-1.17 (-1.27 to -1.07)	-1.18 (-1.29 to -1.06)	-1.01 (-1.09 to -0.93)

ASIR age-standardized incidence rate, *ASMR* age-standardized mortality rate, *ASDR* age-standardized disability-adjusted life-year rate, *EAPC* estimated annual percentage change, *CI* confidence interval

Table S6 Incidence, deaths, and DALYs for subarachnoid hemorrhage (SAH) in 204 countries in 2021

Location	Incidence/1000 (95% UI)			Deaths/1000 (95% UI)			DALYs/1000 (95% UI)		
	Both	Male	Female	Both	Male	Female	Both	Male	Female
Afghanistan	1534.69 (1346.32 − 1740.13)	738.04 (646.36 − 848.88)	796.65 (692.76 − 901.91)	726.21 (382.74 − 1212.60)	398.67 (147.65 − 798.81)	327.54 (86.58 − 546.25)	30,687.15 (17,825.55 − 48,511.74)	16,726.01 (7414.32 − 30,588.86)	13,961.15 (4594.91 − 21,617.64)
Albania	241.98 (214.00 − 270.70)	118.13 (102.89 − 133.91)	123.84 (109.02 − 138.97)	100.75 (65.32 − 139.88)	43.34 (24.69 − 67.97)	57.41 (35.17 − 85.89)	2576.29 (1882.52 – 3418.90)	1165.76 (752.12 − 1730.49)	1410.53 (959.27 − 2042.66)
Algeria	2541.09 (2187.15 − 2873.71)	1219.34 (1065.49 − 1383.66)	1321.75 (1130.05 − 1527.35)	795.2 (511.96 − 1218.69)	351.25 (196.45 − 643.40)	443.95 (261.77 − 753.62)	26,647.75 (18,742.46 − 38,755.54)	11,457.38 (7112.93 − 19,498.49)	15,190.37 (9940.56 − 23,765.15)
American Samoa	5.46 (4.86 − 6.11)	2.60 (2.29 − 2.97)	2.85 (2.52 − 3.23)	2.68 (2.13 − 3.42)	1.17 (0.86 − 1.61)	1.51 (1.14 − 1.98)	100.16 (80.00 − 124.59)	50.21 (37.94 − 67.24)	49.95 (39.18 − 63.36)
Andorra	7.97 (6.90 − 9.27)	3.33 (2.80 − 3.99)	4.64 (4.01 − 5.32)	2.82 (1.91 − 3.82)	0.84 (0.48 − 1.22)	1.98 (1.26 − 2.81)	80.50 (59.00 − 106.41)	26.97 (18.12 − 36.78)	53.53 (37.68 − 72.52)
Angola	1209.00 (1026.82 − 1391.51)	638.85 (541.36 − 739.74)	570.15 (482.74 − 671.57)	360.18 (188.29 − 662.81)	198.22 (76.48 − 468.68)	161.96 (99.96 − 323.00)	15,347.95 (9101.51 – 26,373.12)	8572.31 (4057.64 − 18,167.82)	6775.63 (4646.16 − 12,111.82)
Antigua and Barbuda	10.59 (9.28 − 11.85)	4.56 (3.97 − 5.17)	6.04 (5.27 − 6.96)	4.13 (3.82 − 4.47)	1.92 (1.72 − 2.15)	2.21 (2.02 − 2.41)	127.99 (119.32 − 137.72)	58.04 (52.48 − 64.09)	69.95 (64.53 − 76.66)

Argentina	5583.39 (4971.78 − 6303.69)	2257.11 (2013.02 − 2567.39)	3326.29 (2941.57 − 3810.99)	2218.52 (2029.62 − 2373.98)	992.73 (904.02 − 1073.41)	1225.79 (1105.00 − 1337.87)	70,009.21 (64,857.55 − 74,938.90)	31,946.86 (29,332.83 − 34,772.48)	38,062.35 (35,051.50 − 41,502.46)
Armenia	225.86 (200.57 − 252.96)	115.31 (102.23 − 128.49)	110.55 (95.86 − 125.56)	97.76 (87.19 − 110.31)	51.58 (45.73 − 57.88)	46.18 (40.28 − 52.53)	2816.67 (2508.63 − 3187.89)	1608.29 (1433.74 − 1799.42)	1208.38 (1059.20 − 1384.55)
Australia	2226.4 (1969.18 − 2538.89)	896.86 (792.03 − 1020.74)	1329.54 (1164.55 − 1523.66)	1,080.42 (946.80 − 1,178.80)	465.68 (419.55 − 511.93)	614.74 (519.50 − 680.09)	26,840.85 (24,677.84 − 28,931.76)	11,667.59 (10,770.69 − 12,592.48)	15,173.25 (13,697.92 − 16,676.88)
Austria	1239.61 (1072.25 − 1429.03)	534.28 (450.36 − 626.70)	705.33 (601.43 − 809.97)	424.91 (370.90 − 467.94)	167.42 (149.16 − 184.12)	257.49 (214.74 − 290.00)	11,325.27 (10,132.50 − 12,465.83)	4692.03 (4221.14 − 5190.59)	6633.24 (5865.52 − 7393.10)
Azerbaijan	621.77 (525.01 − 725.82)	313.51 (261.83 − 367.61)	308.26 (260.38 − 361.68)	106.49 (69.91 − 151.97)	51.60 (28.42 − 84.08)	54.89 (30.27 − 89.57)	4441.45 (3319.27 − 5866.28)	2214.17 (1446.23 − 3263.91)	2227.28 (1580.99 − 3159.14)
Bahamas	37.39 (32.96 − 42.04)	15.69 (13.75 − 17.77)	21.71 (18.80 − 24.72)	13.91 (11.29 − 17.24)	6.41 (5.17 − 8.07)	7.51 (6.13 − 9.32)	508.89 (417.00 − 625.22)	237.12 (193.13 − 297.46)	271.77 (221.21 − 333.00)
Bahrain	61.62 (50.68 − 72.58)	37.65 (30.46 − 45.28)	23.97 (19.98 − 27.96)	14.60 (10.93 − 18.91)	8.19 (5.15 − 12.13)	6.41 (4.79 − 8.89)	621.90 (492.22 − 776.68)	368.41 (265.36 − 501.27)	253.5 (202.46 − 324.96)
Bangladesh	19,095.71 (16,867.10 − 21,923.94)	11,460.58 (10,055.90 − 13,152.99)	7635.13 (6619.94 − 8856.16)	10,874.09 (6356.78 − 18,041.40)	6455.33 (2757.30 − 13,618.49)	4418.76 (2852.19 − 7457.33)	368,375.40 (234,653.89 − 583,507.42)	228,644.86 (113,471.13 − 447,747.29)	139,730.53 (93,452.44 − 214,920.84)

Barbados	41.43 (37.17 − 47.17)	18.94 (16.81 − 21.65)	22.50 (19.74 − 26.11)	22.70 (18.17 − 28.06)	10.70 (8.40 − 13.50)	12.00 (9.52 − 14.85)	637.16 (505.37 − 786.19)	301.62 (237.57 − 382.77)	335.53 (266.25 − 421.53)
Belarus	948.23 (848.85 − 1062.92)	430.12 (379.75 − 491.20)	518.12 (461.43 − 588.71)	594.21 (483.06 − 706.86)	298.31 (240.66 − 362.74)	295.9 (242.46 − 347.37)	17,141.25 (14,053.93 − 20,345.71)	9644.88 (7766.08 − 11,681.48)	7496.36 (6192.45 − 8777.40)
Belgium	1027.04 (912.00 − 1175.85)	441.87 (388.83 − 502.42)	585.17 (515.18 − 675.66)	547.68 (476.41 − 595.53)	245.16 (222.49 − 267.45)	302.53 (250.01 − 341.68)	12,760.85 (11,780.47 − 13,743.24)	5806.30 (5400.21 − 6259.55)	6954.55 (6218.15 − 7682.58)
Belize	31.25 (27.67 − 35.01)	13.61 (11.98 − 15.27)	17.64 (15.40 − 20.03)	8.85 (7.72 − 10.04)	4.13 (3.52 − 4.79)	4.72 (4.13 − 5.35)	337.81 (298.01 − 380.55)	159.25 (136.56 − 183.49)	178.56 (157.76 − 202.68)
Benin	490.32 (418.39 − 564.59)	270.83 (230.12 − 310.82)	219.49 (185.52 − 255.04)	149.97 (69.63 − 349.43)	77.02 (22.57 − 276.13)	72.95 (42.53 − 159.00)	6294.81 (3449.82 − 13,226.78)	3409.75 (1363.27 − 10,381.75)	2885.06 (1885.61 − 5501.21)
Bermuda	7.96 (6.97 − 9.13)	3.73 (3.28 − 4.23)	4.23 (3.60 − 4.95)	2.91 (2.46 − 3.50)	1.79 (1.51 − 2.16)	1.12 (0.92 − 1.40)	80.13 (68.64 − 94.44)	47.20 (39.85 − 56.52)	32.93 (27.70 − 39.25)
Bhutan	52.68 (45.26 − 59.98)	28.21 (23.81 − 32.32)	24.47 (21.31 − 28.11)	22.72 (14.88 − 34.83)	11.56 (5.89 − 21.67)	11.17 (7.15 − 17.24)	765.87 (521.57 − 1149.23)	399.33 (222.01 − 714.35)	366.55 (243.59 − 547.94)
Bolivia (Plurinational State of Bolivia)	1485.15 (1316.35 − 1663.76)	602.13 (528.21 − 680.27)	883.02 (776.94 − 1005.11)	908.85 (645.45 − 1276.17)	323.48 (213.10 − 460.45)	585.37 (391.67 − 847.71)	31,155.19 (22,888.72 − 42,571.15)	12,114.64 (8469.69 − 16,879.53)	19,040.55 (13,242.16 − 27,117.94)

Bosnia and Herzegovina	387.15 (350.53 − 428.06)	170.42 (152.79 − 191.76)	216.73 (195.01 − 241.56)	189.92 (137.96 − 245.51)	81.51 (53.72 − 116.24)	108.41 (76.79 − 143.66)	5279.04 (3928.97 − 6756.72)	2385.51 (1639.57 − 3315.24)	2893.53 (2157.05 − 3694.58)
Botswana	109.51 (93.28 − 127.77)	54.27 (46.12 − 63.99)	55.24 (46.95 − 64.65)	19.89 (12.90 − 34.14)	10.61 (6.10 − 22.69)	9.27 (6.30 − 17.95)	848.54 (596.71 − 1352.76)	465.84 (288.38 − 942.09)	382.70 (285.78 − 648.23)
Brazil	25,289.74 (21,783.79 − 29,544.03)	9913.47 (8525.51 − 11,539.22)	15,376.27 (13,197.96 − 17,973.86)	13,700.99 (12,895.32 − 14,272.27)	5197.85 (4929.47 − 5474.60)	8503.14 (7848.01 − 8962.32)	449,297.39 (429,646.53 − 466,339.18)	173,468.00 (165,104.63 − 182,237.62)	275,829.40 (260,962.04 − 289,177.31)
Brunei Darussalam	67.29 (58.61 − 78.59)	29.46 (24.83 − 34.73)	37.83 (32.72 − 44.07)	26.11 (21.25 − 31.69)	11.47 (8.59 − 15.63)	14.64 (11.52 − 18.18)	989.43 (829.81 − 1186.82)	475.80 (366.38 − 634.94)	513.62 (419.75 − 626.85)
Bulgaria	900.42 (823.04 − 985.29)	424.60 (379.65 − 473.98)	475.83 (433.10 − 522.10)	663.10 (573.34 − 760.56)	308.64 (262.41 − 358.95)	354.46 (307.88 − 408.26)	16,938.38 (14,548.43 − 19,524.05)	8457.08 (7145.78 − 9956.06)	8481.30 (7293.11 − 9748.48)
Burkina Faso	797.22 (686.89 − 911.74)	426.53 (365.07 − 494.39)	370.69 (316.01 − 425.39)	233.70 (89.04 − 585.63)	154.15 (40.05 − 501.27)	79.55 (43.22 − 182.02)	10,137.66 (4992.22 − 21,931.44)	6573.35 (2472.70 − 18,119.68)	3564.31 (2338.34 − 6716.83)
Burundi	605.39 (519.10 − 699.64)	367.74 (312.62 − 430.59)	237.65 (202.37 − 275.96)	204.74 (49.21 − 724.39)	141.11 (20.35 − 645.76)	63.63 (25.93 − 201.84)	8501.31 (2814.36 − 26,543.67)	5721.83 (1284.41 − 23,229.58)	2779.47 (1395.60 − 7367.05)
Côte d’Ivoire	1007.65 (863.30 − 1152.43)	589.03 (502.64 − 677.97)	418.62 (352.38 − 484.32)	343.66 (167.20 − 736.84)	215.30 (79.10 − 631.36)	128.36 (79.60 − 259.47)	15,993.22 (9038.23 − 30,972.89)	10,269.55 (4,749.09 − 25,989.22)	5723.67 (3934.85 − 10,388.23)

Cabo Verde	27.25 (22.66 − 31.99)	14.12 (11.87 − 16.54)	13.12 (10.72 − 15.64)	7.89 (3.78 − 18.51)	4.38 (1.52 − 14.75)	3.52 (1.90 − 8.78)	321.98 (183.28 − 709.37)	189.85 (79.96 − 559.29)	132.13 (87.32 − 272.94)
Cambodia	1495.21 (1316.14 − 1692.35)	642.81 (558.54 − 732.18)	852.4 (745.30 − 980.75)	670.52 (475.42 − 1044.27)	265.93 (173.33 − 463.71)	404.59 (278.92 − 663.58)	22,819.02 (16,681.61 − 32,966.48)	9900.94 (6617.30 − 15,783.85)	12,918.08 (9404.47 − 18,509.05)
Cameroon	1151.84 (983.63 − 1335.44)	644.29 (554.35 − 753.86)	507.56 (426.56 − 585.11)	424.55 (190.87 − 908.65)	252.84 (81.50 − 718.56)	171.71 (97.50 − 362.22)	19,001.32 (9624.58 − 37,450.38)	11,631.00 (4733.27 − 28,972.28)	7370.32 (4677.30 − 14,064.42)
Canada	3883.29 (3430.04 − 4441.58)	1527.50 (1344.13 − 1781.08)	2355.79 (2049.18 − 2721.29)	1655.34 (1460.06 − 1800.31)	679.43 (611.19 − 743.55)	975.91 (843.82 − 1078.03)	44,084.87 (40,485.46 − 47,579.39)	18,448.62 (16,862.28 − 20,014.60)	25,636.25 (23,152.68 − 28,023.40)
Central African Republic	252.19 (214.88 − 296.86)	139.28 (118.43 − 164.99)	112.91 (95.48 − 134.23)	111.33 (40.86 − 250.56)	68.47 (16.77 − 197.88)	42.86 (17.88 − 103.79)	4610.71 (1,987.36 − 9,667.42)	2894.89 (921.30 − 7528.10)	1715.81 (816.38 − 3775.54)
Chad	642.34 (548.22 − 735.13)	366.69 (309.26 − 422.18)	275.65 (233.34 − 318.31)	250.43 (105.47 − 583.27)	152.09 (44.82 − 493.70)	98.34 (55.17 − 206.54)	11,576.82 (5842.51 − 24,324.68)	7199.74 (2840.87 − 19,688.17)	4377.08 (2646.51 − 8547.62)
Chile	2225.08 (1960.75 − 2514.83)	823.95 (704.14 − 940.47)	1401.13 (1230.65 − 1609.24)	948.87 (873.77 − 1023.39)	407.49 (374.01 − 445.52)	541.38 (486.52 − 592.99)	28,538.95 (26,750.13 − 30,664.66)	12,624.98 (11,585.62 − 13,669.74)	15,913.97 (14,700.59 − 17,290.18)
China	145,138.48 (125,425.42 − 169,016.38)	78,990.17 (67,566.71 − 93,431.54)	66,148.31 (57,391.10 − 76,476.82)	91,802.18 (66,671.88 − 116,215.44)	49,084.73 (29,036.95 − 69,317.92)	42,717.45 (29,206.73 − 57,848.12)	2,296,534.29 (1,727,441.71 − 2,847,370.05)	1,257,996.98 (796,778.55 − 1,713,591.99)	1,038,537.31 (752,194.25 − 1,367,867.96)

Colombia	7570.42 (6798.04 − 8534.63)	2963.21 (2613.97 − 3368.20)	4607.21 (4061.30 − 5229.22)	3353.40 (2803.39 − 3960.97)	1346.94 (1103.01 − 1620.52)	2006.46 (1678.32 − 2368.03)	100,436.71 (84,643.23 − 118,485.56)	42,007.44 (34,912.91 − 50,038.55)	58,429.27 (49,326.05 − 68,039.44)
Comoros	44.52 (38.29 − 52.36)	24.96 (21.42 − 29.27)	19.56 (16.51 − 23.33)	13.36 (5.37 − 31.44)	7.66 (1.79 − 25.28)	5.70 (2.91 − 13.47)	522.13 (253.76 − 1121.88)	301.41 (96.66 − 860.28)	220.72 (135.24 − 450.93)
Congo	243.97 (206.70 − 283.01)	130.45 (109.31 − 152.54)	113.52 (96.16 − 133.28)	79.72 (42.28 − 151.51)	39.06 (14.36 − 100.59)	40.66 (24.79 − 81.97)	3248.73 (1906.14 − 5801.05)	1642.77 (742.45 − 3799.42)	1605.96 (1032.74 − 3009.24)
Cook Islands	2.03 (1.79 − 2.29)	0.90 (0.78 − 1.03)	1.13 (0.99 − 1.30)	0.66 (0.43 − 0.91)	0.29 (0.17 − 0.42)	0.37 (0.22 − 0.57)	24.30 (18.41 − 31.25)	12.26 (8.38 − 16.61)	12.04 (8.74 − 16.79)
Costa Rica	541.04 (470.49 − 616.01)	223.81 (194.45 − 259.67)	317.24 (271.76 − 365.50)	199.87 (173.33 − 221.37)	91.18 (78.80 − 101.29)	108.69 (93.54 − 123.30)	6377.41 (5673.36 − 6991.35)	2940.34 (2597.87 − 3264.89)	3437.07 (3019.20 − 3827.41)
Croatia	391.58 (355.45 − 430.61)	166.98 (148.01 − 186.92)	224.61 (202.91 − 250.16)	299.23 (261.30 − 342.72)	125.39 (108.45 − 145.28)	173.85 (149.93 − 200.25)	6910.34 (6053.79 − 7851.17)	3152.29 (2752.72 − 3609.86)	3758.05 (3268.47 − 4316.02)
Cuba	1277.57 (1126.31 − 1483.76)	532.28 (462.68 − 614.61)	745.29 (647.08 − 885.30)	522.16 (447.07 − 593.85)	226.63 (191.61 − 265.65)	295.53 (251.79 − 340.17)	15,520.54 (13,536.87 − 17,518.75)	6823.75 (5845.03 − 7944.13)	8696.79 (7543.08 − 9988.83)
Cyprus	123.44 (107.89 − 141.82)	55.39 (47.74 − 63.99)	68.05 (59.28 − 78.82)	47.63 (37.64 − 60.37)	20.47 (15.17 − 27.35)	27.16 (19.72 − 35.68)	1257.64 (1044.79 − 1519.86)	577.12 (449.97 − 742.20)	680.52 (550.89 − 837.90)

Czechia	956.85 (857.03 − 1066.05)	442.83 (390.44 − 501.67)	514.02 (457.20 − 577.20)	468.27 (410.44 − 534.55)	200.42 (173.03 − 231.36)	267.85 (230.91 − 305.71)	12,536.82 (11,056.17 − 14,366.04)	5617.56 (4855.15 − 6403.30)	6919.26 (6096.78 − 7966.28)
Democratic People’s Republic of Korea	3649.43 (3191.87 − 4137.08)	1760.99 (1506.72 − 2020.88)	1888.44 (1650.58 − 2138.23)	2432.33 (1456.97 − 4474.06)	1088.50 (568.55 − 2634.69)	1343.83 (828.80 − 2263.13)	70,875.16 (44,787.01 − 120,722.57)	34,729.55 (19,668.47 − 75,677.75)	36,145.61 (23,362.41 − 56,933.53)
Democratic Republic of the Congo	4234.42 (3629.78 − 4964.34)	2453.87 (2070.42 − 2882.92)	1780.55 (1508.62 − 2091.37)	1264.46 (431.66 − 4110.64)	741.74 (132.03 − 3424.99)	522.72 (250.35 − 1424.84)	50,353.05 (21,957.53 − 144,645.63)	30,300.82 (8499.10 − 117,111.30)	20,052.23 (11,458.62 − 46,588.35)
Denmark	654.66 (576.88 − 748.18)	277.89 (237.85 − 321.76)	376.77 (330.78 − 435.87)	326.15 (290.61 − 354.04)	135.13 (120.36 − 147.84)	191.02 (165.64 − 211.12)	7589.44 (6947.78 − 8132.83)	3287.72 (2951.11 − 3573.46)	4301.72 (3925.10 − 4713.80)
Djibouti	71.71 (60.61 − 84.62)	45.01 (38.18 − 53.18)	26.70 (22.07 − 32.07)	20.22 (7.92 − 45.13)	14.48 (4.43 − 39.44)	5.74 (2.92 − 13.24)	856.1 (390.85 − 1773.94)	613.07 (226.20 − 1530.53)	243.03 (144.23 − 489.18)
Dominica	6.85 (6.00 − 7.82)	3.31 (2.88 − 3.80)	3.54 (3.10 − 4.06)	3.59 (2.91 − 4.39)	1.87 (1.39 − 2.47)	1.72 (1.35 − 2.17)	110.06 (90.83 − 135.01)	58.91 (45.15 − 76.36)	51.15 (40.82 − 63.62)
Dominican Republic	1175.47 (1037.38 − 1336.76)	454.90 (398.32 − 516.53)	720.57 (630.60 − 834.72)	419.43 (309.60 − 548.32)	168.76 (115.28 − 243.86)	250.67 (176.49 − 344.26)	15,791.69 (12,272.54 − 19,934.23)	6558.81 (4590.79 − 9036.58)	9232.89 (6820.53 − 12,413.07)
Ecuador	2585.02 (2336.50 − 2906.17)	1127.11 (1010.51 − 1281.30)	1457.90 (1292.44 − 1665.69)	1256.57 (1001.49 − 1554.13)	559.27 (436.80 − 722.68)	697.30 (551.00 − 858.82)	40,189.93 (32,586.06 − 49,510.92)	18,842.19 (15,137.10 − 24,110.63)	21,347.74 (17,270.60 − 26,072.77)

Egypt	4577.52 (3929.51 − 5241.39)	2178.16 (1879.93 − 2477.29)	2399.36 (2046.95 − 2788.90)	1673.83 (1141.50 − 2457.01)	952.65 (608.99 − 1579.00)	721.18 (423.91 − 1229.95)	64,820.56 (47,313.50 − 93,503.97)	36,414.82 (24,837.36 − 57,734.99)	28,405.74 (18,717.26 − 44,515.53)
El Salvador	615.97 (548.02 − 687.01)	227.57 (202.25 − 252.40)	388.41 (343.54 − 442.15)	211.64 (162.80 − 272.37)	76.54 (51.19 − 100.42)	135.10 (97.68 − 177.91)	7379.09 (5845.32 − 9175.76)	2877.37 (1933.23 − 3723.98)	4501.72 (3432.06 − 5759.98)
Equatorial Guinea	48.70 (40.55 − 56.39)	27.03 (22.29 − 31.59)	21.67 (18.08 − 25.76)	9.39 (4.64 − 15.84)	4.65 (1.94 − 9.62)	4.74 (2.03 − 9.94)	439.98 (239.05 − 694.41)	231.42 (118.90 − 437.71)	208.56 (111.23 − 391.19)
Eritrea	358.22 (304.37 − 419.38)	197.39 (166.07 − 234.38)	160.83 (135.38 − 191.98)	122.80 (44.13 − 283.26)	78.10 (17.94 − 228.85)	44.70 (23.67 − 103.98)	5250.00 (2186.24 − 11,402.07)	3445.06 (1007.82 − 9258.03)	1804.95 (1059.48 − 3672.18)
Estonia	120.72 (107.53 − 135.56)	55.08 (48.35 − 62.12)	65.64 (57.67 − 74.45)	65.96 (57.23 − 74.03)	32.69 (28.47 − 37.41)	33.27 (27.40 − 37.84)	1547.44 (1348.79 − 1740.07)	872.16 (765.68 − 994.00)	675.28 (573.75 − 760.45)
Eswatini	41.82 (35.93 − 48.00)	21.10 (18.14 − 24.39)	20.72 (17.64 − 24.01)	13.79 (8.59 − 22.32)	7.23 (3.92 − 13.00)	6.56 (3.75 − 10.86)	555.25 (350.16 − 894.68)	309.95 (181.44 − 538.58)	245.30 (154.47 − 388.48)
Ethiopia	4,82.1 (3638.05 − 4945.19)	2615.17 (2216.80 − 3021.22)	1666.93 (1409.76 − 1947.82)	1025.25 (344.09 − 3099.83)	688.86 (140.10 − 2750.00)	336.39 (165.94 − 907.61)	42,930.72 (18,318.57 − 113,072.26)	27,930.69 (8410.40 − 97,918.73)	15,000.03 (8720.55 − 34,692.11)
Fiji	132.13 (119.02 − 146.39)	41.49 (36.42 − 46.79)	90.64 (81.27 − 101.48)	53.49 (40.36 − 70.99)	19.67 (14.44 − 27.12)	33.82 (24.27 − 46.55)	2153.70 (1687.54 − 2733.81)	908.12 (691.05 − 1198.08)	1245.58 (932.44 − 1654.83)

Finland	874.77 (771.60 − 993.05)	373.01 (325.63 − 431.42)	501.76 (440.82 − 574.62)	400.34 (348.64 − 435.53)	168.35 (150.87 − 181.76)	232.00 (195.88 − 258.41)	10,567.90 (9751.63 − 11,445.02)	4810.79 (4452.91 − 5169.72)	5757.11 (5190.55 − 6382.90)
France	6760.79 (6084.51 − 7534.56)	2947.32 (2608.71 − 3297.97)	3813.47 (3400.82 − 4323.20)	3012.92 (2631.61 − 3283.36)	1271.22 (1150.52 − 1374.03)	1741.70 (1456.29 − 1939.10)	72,173.24 (65,760.27 − 78,075.55)	33,330.30 (30,552.33 − 36,217.39)	38,842.94 (34,636.79 − 43,294.27)
Gabon	83.02 (70.44 − 96.40)	44.30 (37.44 − 52.09)	38.73 (32.41 − 46.01)	21.08 (12.11 − 36.50)	12.16 (5.68 − 25.35)	8.92 (4.64 − 16.55)	836.76 (524.55 − 1382.20)	489.43 (261.80 − 932.80)	347.33 (217.96 − 582.48)
Gambia	85.92 (73.49 − 99.39)	47.57 (40.90 − 55.25)	38.35 (32.26 − 44.39)	35.26 (15.22 − 87.20)	19.67 (5.58 − 71.27)	15.59 (8.40 − 34.12)	1520.21 (756.23 − 3556.87)	895.81 (331.45 − 2862.91)	624.40 (368.59 − 1223.12)
Georgia	831.28 (760.82 − 912.40)	405.80 (364.68 − 451.32)	425.48 (385.70 − 479.11)	522.71 (431.72 − 616.72)	245.80 (197.02 − 298.14)	276.90 (228.86 − 327.11)	12,933.66 (10,722.33 − 15,161.39)	7079.01 (5770.19 − 8603.93)	5854.66 (4875.63 − 6793.77)
Germany	8638.99 (7600.19 − 10,001.42)	3674.14 (3177.88 − 4263.59)	4964.85 (4297.00 − 5792.14)	4090.89 (3540.24 − 4459.91)	1729.36 (1549.33 − 1891.22)	2361.53 (1947.08 − 2631.34)	103,377.59 (94,795.23 − 111,589.09)	44,721.66 (41,165.68 − 48,268.40)	58,655.93 (52,880.17 − 64,151.90)
Ghana	1569.86 (1334.46 − 1,818.74)	856.61 (730.96 − 995.56)	713.25 (595.85 − 841.12)	625.10 (322.94 − 1243.40)	340.42 (123.63 − 928.25)	284.68 (173.32 − 553.44)	26,770.80 (14,853.99 − 50,608.87)	15,520.79 (6497.14 − 38,995.68)	11,250.02 (7459.27 − 20,388.40)
Greece	1234.24 (1093.06 − 1413.49)	544.25 (472.14 − 621.45)	689.99 (603.46 − 795.02)	702.33 (617.77 − 759.86)	325.06 (293.28 − 353.47)	377.27 (317.62 − 416.16)	16,241.38 (15,015.66 − 17,522.97)	7945.86 (7246.03 − 8565.63)	8295.52 (7518.93 − 9066.38)

Greenland	7.48 (6.70 − 8.41)	3.37 (2.97 − 3.86)	4.11 (3.69 − 4.64)	4.90 (3.68 − 6.12)	2.11 (1.15 − 2.83)	2.80 (2.20 − 3.54)	160.97 (124.87 − 195.15)	69.56 (41.47 − 92.20)	91.42 (74.44 − 115.18)
Grenada	11.42 (10.19 − 12.96)	4.85 (4.22 − 5.63)	6.57 (5.80 − 7.52)	5.28 (4.59 − 5.97)	2.41 (2.03 − 2.76)	2.86 (2.49 − 3.26)	171.70 (147.35 − 194.93)	81.05 (68.51 − 92.55)	90.65 (78.56 − 104.19)
Guam	18.54 (16.49 − 20.73)	8.63 (7.65 − 9.75)	9.91 (8.82 − 11.15)	5.80 (4.82 − 6.87)	2.81 (2.18 − 3.66)	2.99 (2.31 − 3.73)	250.82 (215.72 − 292.88)	133.79 (109.39 − 166.33)	117.03 (97.03 − 139.84)
Guatemala	1198.23 (1065.64 − 1345.01)	519.84 (459.76 − 587.43)	678.39 (596.66 − 770.22)	300.86 (256.65 − 347.89)	141.80 (117.06 − 165.84)	159.06 (133.59 − 184.89)	12,478.24 (10,724.55 − 14,233.55)	5929.18 (5018.87 − 6851.13)	6549.05 (5608.67 − 7536.45)
Guinea	554.97 (479.73 − 633.15)	316.83 (272.95 − 366.37)	238.14 (203.14 − 275.13)	202.33 (91.65 − 440.90)	109.97 (33.73 − 338.40)	92.36 (54.18 − 209.21)	9022.69 (4897.76 − 17,588.95)	5087.26 (2148.55 − 13,208.57)	3935.43 (2561.80 − 7567.36)
Guinea-Bissau	76.46 (64.55 − 88.27)	41.65 (35.08 − 48.36)	34.8 (29.54 − 40.26)	40.79 (17.07 − 89.93)	22.48 (5.99 − 71.23)	18.3 (10.04 − 39.00)	1822.08 (849.90 − 3821.58)	1047.38 (349.79 − 2986.93)	774.70 (453.99 − 1553.56)
Guyana	80.84 (72.78 − 90.40)	35.54 (31.87 − 39.70)	45.31 (40.47 − 51.75)	37.48 (28.77 − 47.93)	19.52 (14.71 − 25.05)	17.95 (13.56 − 23.00)	1402.46 (1080.68 − 1786.78)	743.14 (569.50 − 947.90)	659.32 (499.95 − 847.09)
Haiti	1464.4 (1290.88 − 1659.95)	606.96 (528.34 − 693.83)	857.44 (747.80 − 971.51)	1077.60 (568.22 − 1681.55)	421.31 (186.90 − 795.37)	656.29 (222.42 − 1068.50)	45,553.99 (24,479.19 − 66,863.52)	17,300.25 (8880.76 − 28,796.04)	28,253.74 (9637.76 − 43,560.81)

Honduras	920.07 (819.19 − 1035.64)	375.62 (331.27 − 421.14)	544.45 (478.93 − 617.50)	774.85 (542.42 − 1012.95)	277.43 (172.40 − 406.17)	497.42 (325.35 − 687.57)	25,507.01 (17,323.85 − 34,149.77)	9349.25 (5765.56 − 13,956.93)	16,157.76 (10,297.74 − 22,413.86)
Hungary	848.18 (766.51 − 942.09)	348.67 (310.53 − 389.31)	499.51 (452.58 − 559.87)	381.26 (324.13 − 437.16)	156.40 (134.32 − 180.06)	224.87 (190.50 − 259.44)	11,389.01 (9775.44 − 13,016.55)	4,924.69 (4269.08 − 5613.96)	6464.33 (5497.68 − 7438.95)
Iceland	34.39 (30.37 − 39.56)	16.46 (14.30 − 19.22)	17.92 (15.62 − 20.73)	12.29 (10.40 − 13.74)	5.00 (4.33 − 5.64)	7.29 (5.98 − 8.27)	316.20 (284.32 − 350.03)	143.08 (127.47 − 158.86)	173.12 (153.08 − 193.96)
India	101,503.37 (86,895.35 − 118,265.62)	53,959.99 (45,889.42 − 63,232.67)	47,543.38 (40,775.29 − 55,450.18)	48,284.62 (33,671.85 − 66,531.02)	26,849.20 (13,299.45 − 43,696.33)	21,435.43 (15,961.55 − 29,437.49)	1,657,353.60 (1,212,105.37 − 2,198,269.51)	931,950.01 (521,672.35 − 1,439,379.61)	725,403.59 (558,930.04 − 970,274.14)
Indonesia	29,374.98 (25,055.82 − 34,149.62)	14,009.73 (11,854.27 − 16,422.74)	15,365.25 (13,129.84 − 17,924.10)	14,949.13 (10,996.66 − 22,995.92)	6564.25 (3798.69 − 11,412.74)	8384.87 (5234.01 − 13,349.73)	542,472.01 (421,992.91 − 753,770.57)	254,540.77 (164,454.57 − 418,494.09)	287,931.24 (194,850.02 − 419,814.98)
Iran (Islamic Republic of Iran)	3971.54 (3395.11 − 4544.82)	1890.21 (1612.05 − 2165.37)	2081.33 (1762.61 − 2406.35)	1005.82 (786.71 − 1212.57)	485.50 (339.03 − 646.35)	520.32 (399.25 − 634.58)	33,898.63 (28,616.20 − 40,648.70)	16,532.87 (12,610.84 − 21,517.99)	17,365.75 (14,300.88 − 21,461.09)
Iraq	1676.02 (1428.11 − 1905.89)	798.31 (674.88 − 915.16)	877.70 (746.02 − 1008.34)	581.50 (417.59 − 786.57)	291.61 (197.45 − 409.51)	289.89 (199.44 − 404.10)	23,351.88 (17,842.97 − 30,730.60)	11,636.24 (8387.46 − 15,552.30)	11,715.64 (8657.67 − 15,652.41)
Ireland	499.88 (442.87 − 575.51)	203.86 (178.11 − 234.00)	296.02 (260.27 − 343.55)	213.20 (185.30 − 234.49)	91.61 (81.98 − 101.91)	121.59 (100.45 − 137.19)	5338.20 (4881.03 − 5806.33)	2401.15 (2196.14 − 2638.16)	2937.05 (2590.24 − 3242.95)

Israel	605.78 (522.51 − 697.88)	281.19 (240.36 − 325.29)	324.58 (277.32 − 378.54)	168.17 (148.22 − 184.94)	96.00 (86.21 − 106.15)	72.17 (61.03 − 80.81)	4646.90 (4226.13 − 5067.46)	2547.40 (2314.46 − 2783.00)	2099.50 (1863.34 − 2340.86)
Italy	5151.36 (4585.79 − 5770.49)	2159.24 (1904.62 − 2436.40)	2992.13 (2670.02 − 3367.88)	2964.51 (2531.17 − 3219.10)	1248.90 (1130.76 − 1324.13)	1715.61 (1394.57 − 1904.91)	61,278.41 (55,770.61 − 66,042.38)	27,102.85 (25,354.46 − 28,881.16)	34,175.55 (30,242.41 − 37,297.47)
Jamaica	351.00 (314.58 − 394.28)	140.64 (124.21 − 160.80)	210.36 (186.13 − 239.69)	148.86 (115.90 − 189.64)	62.01 (46.38 − 80.97)	86.85 (67.68 − 110.18)	4600.41 (3582.35 − 5838.54)	1879.89 (1440.10 – 2471.13)	2720.52 (2104.19 − 3440.84)
Japan	37,011.29 (32,479.52 − 42,503.85)	13,565.76 (11,782.04 − 15,809.76)	23,445.52 (20,386.05 − 27,189.32)	14,559.96 (12,000.36 − 16,026.64)	5490.75 (5140.62 − 5729.09)	9069.21 (6869.84 − 10,354.10)	388,913.3 (344,185.46 − 426,023.17)	169,485.66 (157,138.01 − 182,011.17)	219,427.64 (187,697.19 − 244,982.01)
Jordan	490.88 (407.61 − 574.76)	264.36 (222.43 − 307.63)	226.51 (184.48 − 268.46)	38.12 (27.66 − 49.08)	19.93 (12.29 − 28.29)	18.19 (12.45 − 24.94)	2460.34 (1963.10 − 2999.05)	1300.16 (960.92 − 1682.93)	1160.18 (914.98 − 1476.87)
Kazakhstan	1852.81 (1645.98 − 2077.09)	978.39 (856.22 − 1123.83)	874.42 (777.12 − 980.84)	1057.07 (909.20 − 1217.86)	507.74 (425.19 − 599.75)	549.34 (466.72 − 639.69)	31,554.73 (26,965.96 − 36,229.48)	16,789.09 (14,071.40 − 19,714.56)	14,765.64 (12,627.65 − 17,158.74)
Kenya	2161.52 (1843.53 − 2498.53)	1252.51 (1064.79 − 1447.02)	909.01 (770.38 − 1062.26)	502.16 (223.46 − 1215.21)	300.35 (98.58 − 948.80)	201.80 (104.04 − 571.88)	20,912.59 (10,865.31 − 44,370.20)	12,653.06 (5148.50 − 34,733.40)	8259.53 (5043.20 − 20,456.05)
Kiribati	21.31 (18.68 − 24.12)	11.04 (9.16 − 12.89)	10.27 (9.05 − 11.65)	7.35 (5.24 − 10.68)	4.86 (3.07 − 8.17)	2.49 (1.75 − 3.41)	354.62 (256.81 − 491.14)	243.90 (164.72 − 378.04)	110.72 (81.89 − 144.73)

Kuwait	250.15 (200.09 − 302.14)	123.72 (100.60 − 148.14)	126.43 (98.77 − 155.27)	15.98 (13.22 − 19.34)	11.22 (9.04 − 14.02)	4.77 (4.03 − 5.57)	1126.21 (936.42 − 1330.90)	643.96 (530.30 − 767.65)	482.24 (389.59 − 595.75)
Kyrgyzstan	564.29 (502.58 − 629.38)	304.26 (269.23 − 343.90)	260.03 (231.15 − 292.06)	301.38 (251.17 − 358.07)	174.87 (141.98 − 210.04)	126.51 (105.59 − 152.73)	10,558.35 (8725.60 − 12,536.73)	6343.40 (5167.97 − 7602.76)	4214.95 (3497.51 − 5089.89)
Lao People's Democratic Republic	571.33 (498.31 − 650.21)	281.71 (244.58 − 324.19)	289.62 (251.96 − 331.04)	275.17 (194.80 − 404.90)	131.54 (73.70 − 228.15)	143.64 (86.45 − 226.69)	10,871.26 (8037.53 − 15,242.21)	5483.49 (3410.61 − 8952.14)	5387.77 (3506.13 − 7809.14)
Latvia	198.15 (176.41 − 222.37)	90.20 (79.68 − 101.35)	107.95 (94.78 − 124.00)	122.44 (107.70 − 137.72)	53.67 (45.96 − 62.07)	68.77 (60.24 − 77.88)	2961.82 (2614.68 − 3352.06)	1487.76 (1270.67 − 1735.41)	1474.06 (1296.35 − 1677.90)
Lebanon	326.77 (279.93 − 369.03)	143.82 (123.27 − 162.99)	182.95 (157.38 − 208.64)	130.53 (103.19 − 164.02)	69.38 (49.73 − 92.93)	61.15 (44.94 − 79.97)	3676.20 (3055.91 − 4408.26)	1895.23 (1426.25 − 2435.91)	1780.97 (1445.41 − 2222.84)
Lesotho	82.35 (71.38 − 93.56)	37.10 (31.59 − 42.54)	45.24 (38.80 − 52.29)	38.09 (22.20 − 64.35)	19.80 (10.03 − 36.72)	18.29 (9.99 − 32.16)	1365.16 (827.30 − 2258.12)	754.50 (397.45 − 1372.25)	610.66 (357.85 − 1012.46)
Liberia	207.21 (174.91 − 243.38)	120.64 (101.41 − 143.34)	86.57 (72.95 − 101.83)	77.89 (26.26 − 235.91)	43.61 (7.58 − 198.62)	34.28 (16.60 − 93.40)	3515.31 (1452.24 − 9517.07)	2029.56 (548.08 − 7866.03)	1485.75 (834.29 − 3566.29)
Libya	332.89 (281.20 − 386.40)	153.03 (128.03 − 179.39)	179.87 (152.46 − 210.62)	114.23 (65.96 − 177.86)	44.16 (24.02 − 83.48)	70.07 (33.86 − 111.84)	4863.90 (2938.65 − 7359.76)	1958.08 (1208.13 − 3321.29)	2905.83 (1543.79 − 4478.64)

Lithuania	267.97 (238.69 − 303.07)	116.58 (103.30 − 132.90)	151.39 (133.88 − 174.90)	169.92 (147.66 − 190.40)	74.57 (63.70 − 85.72)	95.35 (82.32 − 107.02)	4201.30 (3689.91 − 4696.50)	2087.12 (1790.77 − 2388.48)	2114.18 (1831.33 − 2385.72)
Luxembourg	47.14 (40.99 − 54.59)	21.87 (18.76 − 25.42)	25.27 (21.94 − 29.92)	17.54 (15.52 − 19.47)	7.91 (6.98 − 8.96)	9.64 (8.31 − 10.85)	443.27 (402.70 − 491.55)	208.32 (187.00 − 232.52)	234.95 (210.49 − 261.23)
Madagascar	1770.30 (1503.54 − 2078.11)	1050.35 (897.45 − 1242.21)	719.95 (600.16 − 845.96)	736.12 (252.70 − 2030.67)	460.42 (107.09 − 1713.50)	275.69 (135.96 − 670.67)	32,456.78 (13,116.97 − 81,902.68)	20,665.65 (6088.50 − 69,796.34)	11,791.13 (6653.10 − 27,023.09)
Malawi	783.74 (667.19 − 912.03)	460.00 (390.70 − 541.78)	323.74 (272.61 − 378.63)	297.67 (99.54 − 853.78)	183.01 (39.47 − 747.22)	114.66 (50.18 − 327.41)	12,328.54 (4,838.82 − 32,934.30)	7812.61 (2204.56 − 28,636.82)	4515.93 (2335.66 − 11,364.58)
Malaysia	2751.37 (2408.23 − 3100.60)	1282.72 (1119.56 − 1459.34)	1468.65 (1274.78 − 1674.70)	932.45 (717.27 − 1140.61)	382.03 (235.88 − 527.43)	550.42 (402.00 − 725.20)	32,528.88 (25,809.99 − 38,492.27)	14,641.60 (10,384.82 − 19,041.41)	17,887.28 (13,924. − 22,024.35)
Maldives	39.57 (33.31 − 46.27)	24.29 (20.27 − 29.05)	15.27 (12.96 − 17.59)	8.89 (6.83 − 11.31)	4.93 (3.47 − 7.10)	3.97 (3.02 − 5.17)	354.15 (279.54 − 455.92)	218.61 (165.59 − 311.84)	135.54 (107.83 − 171.83)
Mali	826.47 (707.59 − 943.21)	489.95 (415.74 − 561.99)	336.51 (282.31 − 389.47)	266.06 (126.08 − 573.70)	128.43 (41.11 − 415.76)	137.63 (78.34 − 273.52)	12,358.67 (7098.42 − 23,855.83)	6273.06 (2871.03 − 17,161.97)	6085.61 (3768.13 − 11,547.35)
Malta	40.43 (34.94 − 46.50)	18.03 (15.46 − 20.91)	22.41 (19.02 − 26.11)	10.46 (9.21 − 11.75)	4.55 (4.04 − 5.14)	5.92 (5.09 − 6.75)	320.79 (285.49 − 361.84)	141.81 (125.69 − 161.04)	178.97 (157.15 − 201.81)

Marshall Islands	9.04 (7.90 − 10.25)	4.53 (3.93 − 5.20)	4.52 (3.94 − 5.10)	4.68 (2.99 − 6.96)	2.63 (1.35 − 4.34)	2.06 (1.07 − 3.18)	208.39 (138.78 − 300.94)	121.93 (68.04 − 190.75)	86.46 (49.21 − 128.95)
Mauritania	164.94 (140.09 − 192.74)	89.68 (76.01 − 105.32)	75.26 (63.18 − 87.68)	52.79 (23.24 − 121.62)	25.47 (7.28 − 94.54)	27.32 (14.86 − 60.97)	2171.38 (1138.84 − 4635.35)	1112.57 (447.70 − 3544.95)	1058.81 (648.31 − 2122.60)
Mauritius	171.61 (151.72 − 195.38)	78.83 (69.52 − 90.23)	92.78 (81.50 − 107.16)	97.47 (89.77 − 103.96)	47.94 (43.98 − 51.57)	49.53 (44.71 − 53.51)	3138.94 (2870.25 − 3347.17)	1640.35 (1489.47 − 1774.47)	1498.59 (1361.01 − 1624.87)
Mexico	12,882.66 (11,193.66 − 14,846.74)	5411.15 (4705.19 − 6223.49)	7471.51 (6462.49 − 8680.30)	5113.92 (4541.28 − 5694.27)	2367.70 (1994.46 − 2781.54)	2746.22 (2328.33 − 3151.19)	172,718.14 (154,806.56 − 192,396.29)	84,028.15 (71,521.48 − 97,365.69)	88,689.99 (76,596.13 − 101,848.64)
Micronesia (Federated States of Micronesia)	16.62 (14.63 − 18.70)	8.05 (7.01 − 9.17)	8.57 (7.57 − 9.79)	8.84 (5.71 − 12.62)	4.76 (2.73 − 7.76)	4.08 (2.41 − 6.37)	367.48 (247.67 − 505.07)	215.71 (134.82 − 331.41)	151.77 (94.35 − 226.14)
Monaco	3.60 (3.13 − 4.20)	1.49 (1.27 − 1.78)	2.11 (1.82 − 2.46)	1.62 (1.17 − 2.18)	0.51 (0.32 − 0.71)	1.11 (0.74 − 1.56)	42.65 (32.27 − 55.42)	15.00 (10.51 − 19.76)	27.66 (19.73 − 37.87)
Mongolia	329.70 (289.50 − 373.98)	173.59 (149.02 − 201.99)	156.10 (136.91 − 176.75)	263.24 (192.17 − 341.42)	138.08 (87.09 − 203.)	125.16 (92.00 − 167.17)	8234.61 (6170.67 − 10,980.21)	4595.66 (2894.05 − 6740.20)	3638.95 (2736.06 − 4790.46)
Montenegro	39.85 (34.88 − 45.07)	18.32 (15.84 − 20.97)	21.53 (18.90 − 24.33)	19.10 (13.62 − 25.70)	6.69 (4.08 − 10.33)	12.41 (8.01 − 17.75)	530.98 (407.03 − 683.52)	203.55 (141.46 − 291.10)	327.43 (233.76 − 445.86)

Morocco	2445.18 (2129.40 − 2778.28)	1156.05 (1009.13 − 1308.95)	1289.13 (1114.17 − 1470.76)	1111.11 (651.02 − 1761.57)	455.58 (221.94 − 905.80)	655.53 (286.04 − 1056.42)	35,213.52 (21,441.38 − 53,544.01)	13,840.03 (7468.13 − 25,791.33)	21,373.49 (10,262.43 − 33,851.62)
Mozambique	1445.65 (1242.59 − 1674.33)	857.42 (730.24 − 994.14)	588.23 (500.39 − 693.08)	694.00 (222.66 − 1992.21)	491.39 (121.50 − 1781.65)	202.61 (89.30 − 547.92)	27,379.83 (10,081.10 − 74,493.15)	19,822.65 (5813.91 − 67,281.51)	7557.18 (3823.91 − 18,009.91)
Myanmar	4975.62 (4419.14 − 5617.97)	2495.96 (2183.02 − 2855.89)	2479.66 (2187.72 − 2831.76)	2854.08 (2017.94 − 4436.34)	1496.80 (959.53 − 2484.40)	1357.28 (940.69 − 2180.22)	103,279.71 (77,326.03 − 142,870.59)	57,606.64 (38,816. − 87,396.20)	45,673.07 (33,576.65 − 64,893.04)
Namibia	101.37 (87.23 − 117.42)	50.38 (42.98 − 57.72)	50.99 (43.44 − 59.44)	27.05 (17.46 − 43.96)	14.82 (8.89 − 27.52)	12.23 (7.67 − 22.55)	1037.20 (708.86 − 1628.37)	590.37 (363.43 − 1021.63)	446.84 (304.47 − 776.30)
Nauru	1.46 (1.29 − 1.62)	0.70 (0.61 − 0.80)	0.76 (0.67 − 0.85)	0.89 (0.63 − 1.21)	0.39 (0.23 − 0.58)	0.50 (0.32 − 0.70)	42.04 (30.98 − 55.77)	20.09 (12.67 − 28.64)	21.95 (15.26 − 30.11)
Nepal	2283.78 (1968.77 − 2635.52)	1077.61 (923.10 − 1252.45)	1206.17 (1024.47 − 1410.46)	1068.19 (630.31 − 1674.85)	567.53 (247.50 − 1153.04)	500.66 (304.78 − 904.24)	36,133.93 (22,914.28 − 54,335.61)	19,198.76 (9628.18 − 37,069.74)	16,935.17 (11,192.16 − 27,130.54)
Netherlands	1693.99 (1495.57 − 1948.73)	686.48 (596.92 − 793.87)	1007.51 (880.72 − 1174.56)	792.40 (697.82 − 860.64)	328.22 (296.35 − 360.78)	464.18 (398.21 − 514.37)	19,116.60 (17,523.75 − 20,504.29)	8133.43 (7448.74 − 8846.33)	10,983.17 (9938.32 − 12,008.67)
New Zealand	404.06 (355.83 − 460.19)	160.36 (139.26 − 184.34)	243.70 (215.06 − 276.07)	240.43 (214.90 − 261.71)	97.96 (90.19 − 106.07)	142.47 (123.46 − 157.21)	6421.40 (5923.81 − 6890.71)	2731.84 (2537.35 − 2927.76)	3689.56 (3337.63 − 4016.11)

Nicaragua	573.03 (498.02 − 651.34)	226.25 (195.32 − 259.16)	346.78 (299.74 − 396.46)	142.75 (111.83 − 174.44)	55.74 (38.48 − 72.99)	87.01 (64.35 − 111.29)	5484.41 (4476.19 − 6642.19)	2233.02 (1667.26 − 2864.32)	3251.38 (2541.45 − 4051.41)
Niger	916.56 (786.20 − 1048.63)	509.46 (431.42 − 587.58)	407.10 (347.61 − 470.10)	272.18 (106.09 − 665.75)	154.48 (33.22 − 510.29)	117.70 (63.30 − 297.43)	11,983.47 (6065.72 − 25,235.97)	6886.83 (2387.41 − 19,330.77)	5096.64 (3125.07 − 10,703.81)
Nigeria	7451.63 (6273.52 − 8603.02)	3852.81 (3256.98 − 4452.12)	3598.82 (3017.29 − 4164.76)	1651.78 (810.75 − 3669.01)	959.88 (327.57 − 3043.67)	691.91 (403.54 − 1433.00)	77,265.21 (47,024.06 − 148,445.10)	46,001.92 (22,439.88 − 120,010.29)	31,263.29 (21,553.93 − 53,948.42)
Niue	0.25 (0.22 − 0.28)	0.10 (0.09 − 0.12)	0.14 (0.13 − 0.16)	0.14 (0.11 − 0.18)	0.06 (0.04 − 0.08)	0.08 (0.06 − 0.11)	5.29 (4.33 − 6.62)	2.50 (1.90 − 3.38)	2.79 (2.17 − 3.51)
North Macedonia	344.85 (312.59 − 379.36)	147.29 (132.77 − 164.12)	197.56 (178.83 − 220.55)	225.00 (170.92 − 285.92)	80.55 (56.29 − 110.45)	144.44 (102.72 − 191.41)	6415.29 (5011.07 − 8044.85)	2437.59 (1749.06 − 3235.09)	3977.7 (2897.49 − 5132.06)
Northern Mariana Islands	6.57 (5.80 − 7.46)	3.65 (3.16 − 4.24)	2.92 (2.58 − 3.32)	3.15 (2.49 − 4.02)	1.51 (1.11 − 2.09)	1.64 (1.28 − 2.14)	115.85 (92.74 − 144.80)	62.97 (48.16 − 84.60)	52.89 (41.84 − 65.69)
Norway	547.74 (477.16 − 629.35)	241.57 (209.96 − 279.52)	306.17 (267.43 − 352.01)	229.64 (201.32 − 247.79)	99.77 (91.02 − 106.31)	129.87 (110.89 − 142.62)	5553.51 (5076.10 − 6019.25)	2624.70 (2421.31 − 2826.88)	2928.82 (2633.33 − 3203.34)
Oman	322.15 (277.46 − 363.75)	173.87 (148.18 − 197.16)	148.28 (129.75 − 168.79)	31.15 (20.85 − 42.06)	18.02 (11.35 − 28.21)	13.13 (8.30 − 18.38)	1662.46 (1306.76 − 2106.09)	967.86 (715.12 − 1374.94)	694.61 (537.09 − 886.13)

Pakistan	17,598.22 (15,071.32 − 20,382.69)	9001.18 (7665.90 − 10,392.15)	8597.04 (7313.98 − 9992.53)	7368.31 (4886.94 − 11,101.50)	3766.67 (1953.94 − 7013.72)	3601.64 (2119.33 − 5648.52)	300,149.86 (213,300.67 − 421,458.55)	154,761.87 (92,755.66 − 268,978.81)	145,387.99 (92,024.37 − 213,639.49)
Palau	3.08 (2.71 − 3.55)	1.57 (1.36 − 1.81)	1.51 (1.33 − 1.74)	1.28 (0.96 − 1.64)	0.72 (0.48 – 1.00)	0.56 (0.42 − 0.74)	52.71 (40.61 − 65.42)	32.85 (23.37 − 43.08)	19.86 (15.49 − 24.89)
Palestine	207.70 (174.47 − 238.66)	98.23 (81.94 − 113.36)	109.47 (91.63 − 126.55)	23.57 (18.25 − 28.70)	11.46 (7.81 − 15.24)	12.10 (9.22 − 15.40)	1210.67 (1015.31 − 1438.06)	588.46 (458.23 − 746.84)	622.21 (511.88 − 763.45)
Panama	521.86 (462.04 − 588.07)	223.98 (196.17 − 255.54)	297.89 (259.56 − 338.14)	221.44 (173.53 − 265.51)	106.68 (81.69 − 130.65)	114.76 (92.07 − 136.83)	6784.43 (5454.03 − 8048.62)	3244.60 (2526.02 − 3922.13)	3539.83 (2923.28 − 4175.78)
Papua New Guinea	805.64 (705.51 − 903.20)	443.26 (386.17 − 505.75)	362.39 (314.09 − 407.29)	504.94 (340.43 − 731.71)	245.37 (134.47 − 441.36)	259.57 (161.63 − 377.57)	22,851.5 (16,572.05 − 30,613.99)	11,541.92 (7017.34 − 18,635.19)	11,309.58 (7581.23 − 15,717.86)
Paraguay	711.66 (632.87 − 793.84)	306.28 (269.62 − 348.31)	405.38 (359.63 − 460.37)	333.54 (249.61 − 435.58)	128.71 (87.07 − 183.34)	204.83 (145.17 − 274.67)	11,462.69 (8839.32 − 14,748.88)	4602.15 (3202.31 − 6384.99)	6860.55 (5093.49 − 9124.79)
Peru	3772.06 (3341.19 − 4235.87)	1719.17 (1517.17 − 1931.93)	2052.89 (1801.89 − 2346.99)	1476.93 (1061.29 − 1977.84)	631.66 (411.98 − 870.50)	845.27 (576.56 − 1151.15)	55,188.15 (40,896.99 − 70,949.88)	25,387.33 (17,470.04 − 33,956.76)	29,800.82 (21,522.72 − 39,382.91)
Philippines	9471.19 (8154.81 − 10,901.26)	4542.52 (3869.07 − 5256.30)	4928.66 (4244.72 − 5691.49)	3720.96 (3035.96 − 4446.95)	1768.90 (1240.10 − 2345.75)	1952.06 (1498.73 − 2439.43)	150,709.5 (120,818.33 − 177,279.40)	76,018.32 (53,462.09 − 96,147.03)	74,691.18 (59,008.75 − 91,727.42)

Poland	3318.46 (2910.54 − 3855.53)	1594.78 (1387.98 − 1845.70)	1723.68 (1509.01 − 1995.52)	1678.47 (1525.17 − 1830.65)	796.07 (707.78 − 880.91)	882.40 (769.66 − 980.63)	51,621.50 (47,153.90 − 56,396.05)	26,659.8 (23,825.74 − 29,431.65)	24,961.69 (22,249.87 − 27,769.39)
Portugal	1092.22 (944.36 − 1235.12)	480.14 (417.88 − 548.56)	612.08 (509.67 − 697.21)	782.12 (681.47 − 852.09)	328.85 (298.02 − 357.27)	453.28 (376.05 − 504.75)	16,751.03 (15,399.43 − 18,034.68)	7447.06 (6834.82 − 8027.22)	9303.98 (8311.73 − 10,127.79)
Puerto Rico	368.76 (321.36 − 421.27)	140.66 (122.54 − 162.45)	228.10 (197.11 − 266.12)	132.08 (109.08 − 153.80)	53.87 (43.97 − 64.16)	78.21 (64.13 − 91.50)	3580.39 (3025.33 − 4154.94)	1491.17 (1230.16 − 1771.55)	2089.22 (1778.09 − 2431.81)
Qatar	187.75 (151.72 − 227.41)	120.21 (94.48 − 147.01)	67.54 (55.62 − 80.27)	29.36 (20.26 − 42.01)	23.47 (15.45 − 35.29)	5.89 (4.09 − 7.99)	1506.07 (1126.67 − 1988.79)	1174.45 (844.62 − 1641.80)	331.62 (256.73 − 412.13)
Republic of Korea	8825.64 (7695.53 − 10,359.89)	3374.53 (2847.80 − 4023.55)	5451.11 (4686.08 − 6403.42)	3044.19 (2481.61 − 3586.88)	1146.77 (835.48 − 1436.48)	1897.43 (1454.77 − 2351.03)	90,558.76 (78,706.31 − 104,699.58)	39,885.12 (31,784.15 − 48,558.87)	50,673.64 (42,246.13 − 60,083.44)
Republic of Moldova	272.68 (237.22 − 308.68)	140.72 (122.02 − 161.45)	131.97 (113.91 − 152.48)	120.75 (108.64 − 135.55)	66.67 (57.73 − 77.24)	54.08 (47.51 − 61.67)	3618.55 (3235.68 − 4074.43)	2091.80 (1813.17 − 2397.75)	1526.75 (1357.87 − 1710.24)
Romania	3152.63 (2901.62 − 3424.40)	1436.84 (1296.00 − 1580.76)	1715.79 (1568.14 − 1884.19)	1874.89 (1627.43 − 2109.37)	851.55 (720.91 − 990.78)	1023.34 (875.91 − 1163.69)	45,287.46 (38,768.24 − 51,071.02)	22,571.05 (19,009.20 − 26,219.91)	22,716.41 (19,747.74 − 25,509.23)
Russian Federation	20,253.16 (17,894.64 − 23,245.53)	9720.20 (8468.33 − 11,255.42)	10,532.96 (9,271.69 − 12,085.71)	13,480.56 (12,416.88 − 14,527.99)	5968.02 (5336.43 − 6572.80)	7512.54 (6699.71 − 8248.70)	370,212.45 (343,245.15 − 400,092.75)	189,299.50 (169,762.43 − 208,066.50)	180,912.95 (162,531.85 − 199,108.07)

Rwanda	639.91 (543.50 − 738.03)	348.43 (294.66 − 405.00)	291.48 (247.36 − 337.88)	184.12 (65.72 − 478.93)	115.60 (28.57 − 393.85)	68.52 (30.36 − 172.32)	7397.00 (3223.68 − 18,048.47)	4642.17 (1480.96 − 14,595.15)	2754.84 (1507.35 − 6107.47)
Saint Kitts and Nevis	6.86 (6.04 − 7.80)	3.07 (2.68 − 3.51)	3.79 (3.32 − 4.33)	2.90 (2.39 − 3.44)	1.37 (1.13 − 1.63)	1.53 (1.25 − 1.83)	95.42 (79.50 − 114.09)	47.44 (39.42 − 57.17)	47.97 (39.64 − 57.71)
Saint Lucia	21.00 (18.61 − 23.86)	8.50 (7.43 − 9.87)	12.50 (10.96 − 14.25)	8.66 (7.04 − 10.37)	3.47 (2.77 − 4.18)	5.19 (4.26 − 6.22)	273.48 (225.69 − 328.04)	113.11 (91.80 − 134.55)	160.38 (133.51 − 191.98)
Saint Vincent and the Grenadines	10.76 (9.60 − 12.17)	4.74 (4.19 − 5.42)	6.02 (5.33 − 6.86)	4.38 (3.83 − 4.97)	2.07 (1.78 − 2.36)	2.31 (1.98 − 2.66)	147.58 (129.32 − 167.83)	67.11 (58.44 − 76.12)	80.47 (69.56 − 93.10)
Samoa	26.20 (23.46 − 29.56)	11.93 (10.48 − 13.55)	14.27 (12.68 − 16.20)	11.70 (8.22 − 16.58)	5.18 (3.07 − 8.77)	6.53 (4.54 − 9.10)	460.49 (332.31 − 625.23)	233.56 (146.26 − 368.68)	226.93 (164.78 − 306.29)
San Marino	2.87 (2.46 − 3.40)	1.30 (1.11 − 1.52)	1.58 (1.33 − 1.88)	0.52 (0.32 − 0.75)	0.22 (0.12 − 0.34)	0.30 (0.18 − 0.46)	17.05 (12.58 − 22.75)	7.39 (5.30 − 10.30)	9.66 (7.06 − 13.16)
Sao Tome and Principe	10.08 (8.57 − 11.82)	5.44 (4.61 − 6.54)	4.63 (3.88 − 5.42)	3.24 (1.37 − 8.15)	1.61 (0.35 − 6.57)	1.62 (0.95 − 3.69)	142.47 (68.18 − 343.78)	76.86 (23.75 − 278.99)	65.61 (40.74 − 131.78)
Saudi Arabia	1604.26 (1316.61 − 1891.46)	832.54 (664.90 − 1011.11)	771.72 (641.46 − 901.70)	336.04 (214.79 − 465.54)	165.60 (73.61 − 253.54)	170.44 (107.68 − 254.47)	16,539.95 (11,317.84 − 22,019.72)	8193.95 (4264.49 − 11,971.09)	8346.00 (5695.61 − 11,678.85)

Senegal	595.54 (506.38 − 691.25)	328.51 (278.31 − 380.65)	267.02 (223.37 − 312.68)	237.52 (108.55 − 544.97)	128.53 (35.16 − 432.16)	108.99 (64.03 − 249.26)	9847.76 (5069.51 − 21,004.63)	5584.19 (2062.34 − 16,699.14)	4263.57 (2739.08 − 8460.16)
Serbia	1373.21 (1258.09 − 1495.38)	543.76 (489.93 − 597.45)	829.45 (761.10 − 910.64)	757.68 (600.16 − 953.56)	275.47 (193.43 − 372.65)	482.22 (366.31 − 628.55)	19,696.47 (15,842.08 − 23,923.38)	7609.17 (5660.33 − 9916.25)	12,087.3 (9574.65 − 15,463.68)
Seychelles	8.36 (7.23 − 9.60)	4.11 (3.53 − 4.80)	4.25 (3.67 − 4.96)	2.25 (1.50 − 3.27)	1.04 (0.38 − 2.10)	1.21 (0.67 − 1.89)	89.20 (66.43 − 116.62)	44.81 (22.27 − 76.28)	44.39 (30.34 − 61.81)
Sierra Leone	351.57 (299.28 − 399.69)	197.69 (169.88 − 226.91)	153.87 (128.90 − 178.54)	136.51 (58.95 − 360.71)	72.35 (18.22 − 284.44)	64.16 (33.60 − 154.49)	6315.55 (3170.72 − 14,617.01)	3475.46 (1287.35 − 11,348.81)	2840.09 (1645.41 − 5977.10)
Singapore	713.55 (597.65 − 859.53)	317.44 (259.31 − 388.50)	396.11 (329.06 − 483.72)	111.52 (100.10 − 121.40)	44.35 (40.01 − 48.59)	67.17 (58.43 − 74.44)	4757.52 (4160.94 − 5325.43)	2089.94 (1829.91 − 2354.98)	2667.58 (2314.84 − 3034.42)
Slovakia	409.31 (361.00 − 459.32)	187.51 (162.98 − 211.72)	221.8 (195.31 − 251.68)	189.52 (147.78 − 234.63)	78.21 (55.03 − 101.19)	111.31 (81.18 − 145.65)	5837.04 (4790.73 − 7064.08)	2589.81 (1938.82 − 3294.32)	3247.23 (2557.23 − 4111.65)
Slovenia	162.54 (142.95 − 186.10)	71.49 (61.79 − 81.92)	91.05 (79.94 − 104.50)	63.00 (52.89 − 71.93)	25.21 (21.56 − 29.16)	37.79 (30.77 − 44.03)	1582.88 (1353.35 − 1791.26)	703.5 (593.94 − 805.58)	879.38 (742.80 − 1010.02)
Solomon Islands	112.77 (99.91 − 127.72)	51.51 (45.12 − 59.74)	61.26 (53.91 − 69.09)	40.77 (25.84 − 59.32)	21.58 (11.65 − 35.21)	19.19 (11.67 − 30.20)	1647.28 (1130.89 − 2290.45)	931.13 (562.73 − 1409.25)	716.15 (476.09 − 1026.80)

Somalia	1001.64 (849.77 − 1170.70)	593.92 (498.30 − 695.95)	407.71 (342.92 − 486.08)	330.63 (59.38 − 1095.32)	222.07 (17.23 − 933.67)	108.56 (30.76 − 470.07)	14,347.46 (3648.98 − 43,037.79)	9787.95 (1474.81 − 37,194.85)	4559.50 (1750.69 − 16,702.65)
South Africa	2390.06 (2039.24 − 2797.73)	1102.25 (938.98 − 1277.95)	1287.81 (1090.70 − 1528.03)	677.05 (553.16 − 784.29)	301.61 (198.90 − 392.16)	375.44 (295.42 − 444.08)	25,801.10 (21,831.83 − 29,660.51)	12,139.52 (8829.35 − 15,307.73)	13,661.58 (11,164.11 − 16,088.19)
South Sudan	399.91 (339.72 − 461.15)	236.77 (202.83 − 272.57)	163.14 (136.20 − 193.29)	137.86 (56.11 − 316.88)	91.4 (26.04 − 264.56)	46.46 (24.51 − 106.46)	5871.41 (2869.72 − 12,151.43)	3798.14 (1432.15 − 9478.12)	2073.27 (1235.00 − 4176.28)
Spain	4623.60 (4083.83 − 5223.32)	1991.00 (1741.61 − 2259.31)	2632.59 (2320.48 − 2997.14)	2474.26 (2122.66 − 2720.22)	1140.3 (1025.38 − 1245.45)	1333.95 (1081.96 − 1508.69)	53,156.45 (48,538.07 − 57,519.32)	24,860.37 (22,852.85 − 26,836.15)	28,296.08 (24,941.82 − 31,144.73)
Sri Lanka	2615.54 (2304.16 − 2954.90)	1179.22 (1037.64 − 1337.26)	1436.32 (1253.23 − 1638.97)	495.72 (330.61 − 684.68)	229.96 (137.35 − 342.11)	265.77 (171.91 − 389.69)	17,825.05 (13,270.39 − 22,811.94)	8464.03 (5749.78 − 11,967.60)	9361.03 (7005.49 − 12,348.52)
Sudan	1851.3 (1607.52 − 2098.60)	938.68 (818.83 − 1068.29)	912.62 (777.22 − 1045.95)	771.99 (386.66 − 1350.76)	371.13 (147.51 − 819.32)	400.86 (154.57 − 728.82)	32,378.94 (17,157.46 − 53,172.79)	15,044.62 (7145.74 − 29,209.22)	17,334.32 (7658.97 − 29,892.03)
Suriname	64.21 (56.99 − 72.81)	28.19 (24.94 − 31.92)	36.02 (31.65 − 41.49)	28.17 (21.08 − 36.66)	13.19 (9.27 − 18.61)	14.98 (11.15 − 19.52)	992.69 (783.20 − 1248.41)	467.73 (344.87 − 648.16)	524.96 (408.20 − 658.61)
Sweden	1038.10 (894.94 − 1202.39)	480.49 (411.84 − 559.25)	557.61 (478.59 − 649.23)	411.84 (352.30 − 466.38)	156.43 (131.59 − 179.55)	255.41 (209.72 − 294.47)	9604.48 (8563.56 − 10,707.74)	4026.13 (3468.20 − 4608.31)	5578.35 (4854.45 − 6304.65)

Switzerland	765.47 (666.81 − 891.10)	323.96 (277.87 − 377.43)	441.51 (383.37 − 515.11)	332.46 (279.45 − 370.91)	140.1 (125.02 − 154.51)	192.36 (154.83 − 219.42)	7361.69 (6596.10 − 8087.88)	3307.48 (3026.20 − 3616.11)	4054.21 (3534.62 − 4506.76)
Syrian Arab Republic	879.73 (767.05 − 986.19)	446.22 (392.99 − 506.01)	433.51 (373.01 − 493.71)	279.22 (185.27 − 400.02)	147.99 (88.77 − 235.90)	131.23 (85.48 − 190.87)	10,445.98 (7545.48 − 14,292.22)	5314.39 (3494.99 − 7925.51)	5131.59 (3699.41 − 7067.51)
Taiwan (Province of China)	3028.13 (2678.85 − 3453.44)	1387.72 (1223.11 − 1580.99)	1640.41 (1422. − 1924.11)	945.36 (846.02 − 1022.61)	466.42 (421.84 − 506.57)	478.94 (412.08 − 529.35)	29,545.28 (26,678.39 − 32,187.60)	14,671.57 (13,294.60 − 16,098.76)	14,873.71 (13,102.69 − 16,560.59)
Tajikistan	761.02 (667.80 − 854.18)	418.11 (364.44 − 479.95)	342.91 (301.11 − 390.98)	212.32 (150.04 − 287.77)	105.63 (60.26 − 164.51)	106.69 (72.80 − 150.86)	8099.04 (5901.39 − 10,920.35)	4129.71 (2485.66 − 6547.90)	3969.34 (2840.38 − 5424.45)
Thailand	12,269.69 (10,909.05 − 14,011.31)	6298.93 (5568.43 − 7208.36)	5970.76 (5255.81 − 6927.66)	8543.31 (6551.23 − 10,927.66)	4517.17 (3212.70 − 6106.95)	4026.14 (2888.98 − 5298.93)	233,062.81 (183,333.81 − 302,013.56)	136,342.07 (101,956.59 − 184,277.50)	96,720.74 (75,135.19 − 123,182.82)
Timor-Leste	92.56 (80.63 − 104.85)	45.55 (39.39 − 52.15)	47.01 (41.10 − 53.43)	53.86 (30.59 − 95.44)	25.59 (12.68 − 58.94)	28.27 (15.55 − 49.33)	1924.13 (1235.21 − 3120.90)	980.87 (567.20 − 1937.43)	943.26 (557.12 − 1502.53)
Togo	334.96 (288.49 − 387.04)	184.4 (158.34 − 214.46)	150.56 (127.67 − 174.97)	140.56 (57.97 − 361.35)	78.04 (19.05 − 286.37)	62.52 (33.61 − 156.76)	5987.71 (2824.42 − 13,839.38)	3476.41 (1128.42 − 11,140.56)	2511.3 (1486.44 − 5449.89)
Tokelau	0.19 (0.17 − 0.21)	0.08 (0.07 − 0.09)	0.11 (0.10 − 0.13)	0.11 (0.08 − 0.16)	0.05 (0.03 − 0.08)	0.07 (0.05 − 0.09)	4.36 (3.40 − 6.03)	1.94 (1.27 − 3.28)	2.42 (1.73 − 3.05)

Tonga	9.05 (8.05 − 10.15)	3.99 (3.50 − 4.54)	5.06 (4.47 − 5.71)	3.18 (2.18 − 4.69)	1.20 (0.75 − 2.15)	1.98 (1.35 − 2.95)	119.85 (87.23 − 169.30)	55.81 (37.09 − 92.45)	64.04 (47.12 − 87.18)
Trinidad and Tbago	166.61 (148.85 − 189.43)	73.76 (65.76 − 83.99)	92.85 (82.09 − 106.03)	90.64 (69.45 − 115.83)	44.68 (33.49 − 58.32)	45.96 (35.66 − 58.43)	2965.92 (2321.28 − 3758.70)	1514.37 (1153.62 − 1954.36)	1451.55 (1136.25 − 1837.00)
Tunisia	767.91 (664.31 − 875.11)	363.58 (315.39 − 418.66)	404.33 (345.11 − 464.54)	263.35 (139.40 − 445.55)	121.21 (53.86 − 230.81)	142.14 (74.39 − 252.20)	7970.29 (4964.27 − 12,533.00)	3625.55 (2016.48 − 6626.64)	4344.74 (2776.24 − 7083.32)
Turkey	6169.61 (5446.59 − 6954.53)	2920.49 (2556.81 − 3299.99)	3249.12 (2844.71 − 3713.89)	3415.29 (2636.05 − 4257.15)	1773.91 (1269.21 − 2407.07)	1641.38 (1220.45 − 2178.43)	99,852.48 (80,291.96 − 123,128.32)	52,869.42 (39,709.85 − 69,296.15)	46,983.05 (36,856.65 − 59,660.54)
Turkmenistan	563.93 (503.34 − 631.09)	318.47 (279.99 − 362.41)	245.46 (219.98 − 276.74)	350.16 (262.27 − 495.80)	210.56 (143.16 − 351.51)	139.59 (85.11 − 192.55)	12,388.27 (9440.57 − 16,975.60)	7762.46 (5420.33 − 12,377.87)	4625.80 (2896.96 − 6368.75)
Tuvalu	1.85 (1.64 − 2.08)	0.89 (0.77 − 1.01)	0.96 (0.85 − 1.11)	1.05 (0.68 − 1.52)	0.50 (0.28 − 0.87)	0.55 (0.33 − 0.82)	40.50 (27.75 − 56.76)	22.00 (13.26 − 35.70)	18.50 (11.95 − 26.33)
Uganda	1607.48 (1384.23 − 1857.58)	925.08 (795.16 − 1073.65)	682.39 (581.75 − 799.13)	399.37 (148.12 − 1058.35)	273.93 (74.44 − 917.85)	125.44 (63.18 − 319.28)	17,762.41 (8,210.98 − 42,884.09)	12,131.48 (4,260.48 − 36,992.55)	5630.94 (3410.45 − 12,175.08)
Ukraine	5676.21 (5004.12 − 6503.61)	2848.57 (2488.26 − 3290.37)	2827.64 (2485.49 − 3258.87)	3645.61 (2770.94 − 4573.76)	1686.33 (1147.26 − 2301.37)	1959.29 (1367.09 − 2723.78)	94,973.97 (71,883.26 − 118,614.15)	51,040.73 (34,805.97 − 69,218.29)	43,933.25 (30,651.32 − 60,287.28)

United Arab Emirates	542.19 (427.08 − 668.26)	357.92 (277.53 − 452.73)	184.27 (148.73 − 224.12)	108.02 (79.68 − 143.28)	66.52 (46.52 − 94.57)	41.50 (28.73 − 56.21)	5088.33 (4053.37 − 6405.76)	3293.14 (2506.10 − 4399.25)	1795.19 (1380.55 − 2303.95)
United Kingdom	7198.45 (6369.08 − 8166.66)	2854.47 (2493.45 − 3270.10)	4343.98 (3851.66 − 4923.13)	3927.12 (3515.88 − 4140.82)	1546.37 (1442.48 − 1606.16)	2380.75 (2077.43 − 2543.75)	91,480.24 (86,290.63 − 96,130.90)	37,995.65 (36,109.53 − 39,698.69)	53,484.60 (49,748.35 − 56,667.98)
United Republic of Tanzania	2552.67 (2192.55 − 2944.60)	1441.15 (1232.44 − 1676.09)	1111.52 (944.51 − 1299.34)	625.34 (244.56 − 1704.66)	380.27 (92.42 − 1457.43)	245.07 (122.84 − 555.52)	26,522.26 (12,745.68 − 64,684.43)	16,351.04 (5779.16 − 54,275.74)	10,171.22 (5962.75 − 19,912.03)
United States of America	29,757.84 (26,203.88 − 34,291.00)	12,307.46 (10,763.88 − 14,120.81)	17,450.38 (15,245.10 − 20,223.94)	18,044.53 (16,291.84 − 19,093.20)	7470.84 (6947.25 − 7859.94)	10,573.69 (9288.51 − 11,332.28)	481,261.46 (452,792.89 − 508,845.19)	203,350.05 (192,684.85 − 214,164.26)	277,911.41 (257,859.64 − 295,747.98)
United States Virgin Islands	12.78 (11.25 − 14.51)	5.48 (4.81 − 6.24)	7.30 (6.36 − 8.49)	3.97 (2.99 − 5.13)	2.03 (1.46 − 2.88)	1.93 (1.38 − 2.72)	120.07 (92.64 − 152.59)	60.96 (44.97 − 83.23)	59.11 (44.21 − 80.40)
Uruguay	608.58 (548.13 − 679.35)	212.39 (190.68 − 240.81)	396.20 (349.54 − 446.55)	354.16 (324.52 − 381.12)	129.95 (117.80 − 141.05)	224.21 (200.40 − 244.04)	9603.19 (8914.60 − 10,263.93)	3835.65 (3504.47 − 4160.03)	5767.54 (5315.75 − 6207.58)
Uzbekistan	2063.02 (1793.11 − 2334.85)	1130.39 (971.59 − 1284.06)	932.63 (796.37 − 1061.39)	1094.56 (914.44 − 1327.23)	660.80 (546.11 − 818.11)	433.76 (362.67 − 519.56)	34,891.39 (29,824.73 − 41,371.44)	21,603.58 (18,264.89 − 26,071.36)	13,287.81 (11,365.10 − 15,667.70)
Vanuatu	40.13 (35.50 − 45.04)	20.50 (18.01 − 23.41)	19.62 (17.24 − 22.13)	18.69 (11.65 − 27.60)	10.42 (5.99 − 18.19)	8.26 (3.52 − 12.92)	846.60 (572.56 − 1223.17)	500.83 (312.66 − 815.10)	345.76 (180.65 − 525.60)

Venezuela (Bolivarian Republic of Venezuela)	3643.01 (3244.55 − 4223.40)	1388.36 (1211.25 − 1602.81)	2254.66 (1981.51 − 2622.87)	1627.65 (1206.80 − 2110.47)	648.84 (471.04 − 866.30)	978.80 (707.21 − 1280.83)	55,022.54 (41,131.60 − 70,454.58)	22,348.21 (16,629.98 − 29,472.14)	32,674.33 (24,107.52 − 42,362.36)
Viet Nam	10,871.03 (9514.81 − 12,535.68)	5594.07 (4845.55 − 6518.70)	5276.96 (4607.13 − 6076.13)	4925.50 (2830.24 − 8829.73)	2596.26 (1287.50 – 4 905.39)	2329.24 (1389.51 − 4700.93)	158,571.90 (105,632.77 − 248,409.91)	91,624.28 (52,638.78 − 154,717.37)	66,947.61 (47,676.90 − 110,889.03)
Yemen	1615.19 (1418.98 − 1807.11)	765.69 (669.39 − 868.09)	849.50 (738.55 − 963.29)	784.65 (373.99 − 1445.49)	410.71 (144.74 − 895.80)	373.94 (156.48 − 783.64)	29,475.80 (15,799.29 − 48,741.58)	14,789.09 (6322.19 − 28,833.65)	14,686.71 (6,905.98 − 26,860.15)
Zambia	842.93 (718.60 − 971.98)	506.59 (434.63 − 589.13)	336.34 (284.26 − 391.71)	281.59 (124.51 − 591.04)	172.36 (57.79 − 478.24)	109.23 (57.99 − 257.97)	11,534.78 (5692.32 − 22,740.92)	7161.02 (2797.97 − 18,274.02)	4373.76 (2632.14 − 9406.17)
Zimbabwe	865.10 (754.11 − 985.62)	463.08 (404.11 − 528.06)	402.01 (345.84 − 468.47)	444.33 (293.58 − 708.67)	235.70 (145.32 − 414.30)	208.62 (126.78 − 338.31)	17,404.23 (11,843.38 − 26,503.73)	9809.48 (6499.23 − 16,427.68)	7594.75 (4812.69 − 11,566.36)

DALYs disability-adjusted life-years, *UI* uncertainty interval

Table S7 ASIR, ASMR, and ASDR for subarachnoid hemorrhage (SAH) in 204 countries in 2021

Location	ASIR/100,000 persons (95% UI)			ASMR/100,000 persons (95% UI)			ASDR/100,000 persons (95% UI)		
	**Both**	**Male**	**Female**	**Both**	**Male**	**Female**	**Both**	**Male**	**Female**
Afghanistan	8.88 (7.88 − 10.14)	8.94 (7.80 − 10.27)	8.86 (7.90 − 10.08)	6.60 (3.24 − 11.83)	7.76 (2.46 − 16.74)	5.52 (1.49 − 10.12)	186.26 (101.62 − 309.66)	209.59 (83.69 − 420.17)	165.42 (52.85 − 266.91)
Albania	6.73 (5.93 − 7.56)	6.92 (6.04 − 7.75)	6.61 (5.75 − 7.42)	2.49 (1.61 − 3.46)	2.34 (1.34 − 3.66)	2.61 (1.61 − 3.92)	69.05 (50.78 − 91.71)	65.92 (42.56 − 95.86)	71.75 (49.16 − 102.32)
Algeria	6.27 (5.48 − 7.07)	6.06 (5.30 − 6.85)	6.44 (5.55 − 7.32)	2.60 (1.72 − 3.95)	2.22 (1.25 − 3.94)	3.14 (1.71 − 4.97)	69.50 (48.57 − 99.79)	59.10 (36.61 − 100.26)	81.47 (52.34 − 127.29)
American Samoa	11.56 (10.39 − 12.83)	10.65 (9.47 − 11.98)	12.38 (11.09 − 13.96)	6.03 (4.83 − 7.57)	4.85 (3.56 − 6.66)	7.07 (5.41 − 9.21)	202.28 (164.02 − 251.73)	195.44 (149.58 − 260.78)	207.59 (161.59 − 261.46)
Andorra	6.10 (5.27 − 6.98)	5.19 (4.42 − 6.06)	7.08 (6.13 − 8.02)	1.80 (1.22 − 2.44)	1.12 (0.64 − 1.62)	2.47 (1.59 − 3.50)	57.46 (41.75 − 75.52)	38.68 (26.40 − 52.26)	77.28 (54.56 − 104.80)
Angola	6.31 (5.46 − 7.40)	7.11 (6.06 − 8.25)	5.67 (4.85 − 6.71)	2.74 (1.43 − 5.16)	3.31 (1.20 − 8.33)	2.31 (1.43 − 4.93)	88.71 (50.99 − 154.18)	104.45 (46.28 − 234.63)	75.47 (51.27 − 139.61)
Antigua and Barbuda	10.34 (9.19 − 11.48)	9.54 (8.42 − 10.66)	11.19 (9.88 − 12.66)	3.99 (3.71 − 4.30)	4.12 (3.75 − 4.56)	3.95 (3.62 − 4.31)	118.60 (110.98 − 127.41)	114.75 (104.41 − 125.55)	122.69 (113.55 − 133.57)
Argentina	10.86 (9.70 − 12.25)	9.49 (8.49 − 10.73)	12.18 (10.70 − 13.90)	4.06 (3.72 − 4.34)	4.17 (3.80 − 4.51)	4.01 (3.62 − 4.36)	134.14 (124.15 − 143.62)	132.47 (121.54 − 144.07)	136.28 (125.94 − 147.98)
Armenia	6.16 (5.46 − 6.90)	7.03 (6.25 − 7.84)	5.42 (4.65 − 6.17)	2.34 (2.08 − 2.64)	2.96 (2.63 − 3.32)	1.83 (1.58 − 2.08)	71.12 (63.51 − 80.44)	92.59 (82.77 − 103.61)	52.82 (46.08 − 60.61)

Australia	6.11	5.21	6.98	2.38	2.30	2.46	70.06	65.21	74.54
	(5.37 − 6.92)	(4.55 − 5.94)	(6.04 − 8.05)	(2.13 − 2.59)	(2.08 − 2.52)	(2.14 − 2.70)	(64.90 − 75.38)	(60.33 − 70.40)	(67.84 − 81.71)
Austria	8.86	8.19	9.52	2.22	2.11	2.32	71.68	65.92	76.82
	(7.58 − 10.19)	(6.95 − 9.50)	(8.04 − 10.98)	(1.98 − 2.42)	(1.89 − 2.30)	(2. 00 − 2.58)	(64.60 − 78.56)	(59.64 − 72.33)	(68.82 − 85.49)
Azerbaijan	5.85	6.14	5.53	1.10	1.18	1.03	39.40	41.36	37.44
	(5.03 − 6.68)	(5.26 − 7.06)	(4.72 − 6.37)	(0.74 − 1.57)	(0.66 − 1.90)	(0.56 − 1.67)	(29.64 − 51.87)	(27 − 61.29)	(26.61 − 53.09)
Bahamas	8.97	8.14	9.76	3.31	3.36 2.73 −	3.28	115.57	115.16	115.71
	(7.98 − 9.97)	(7.20 − 9.08)	(8.55 − 11.01)	(2.70 − 4.08)	4.15)	(2.69 − 4.07)	(94.93 − 141.03)	(94.13 − 143.51)	(94.78 − 141.63)
Bahrain	4.48	4.38	4.72	1.99	1.96	2.09	52.26	49.39	57.26
	(3.90 − 5.04)	(3.78 − 4.97)	(4.09 − 5.35)	(1.45 − 2.51)	(1.09 − 2.82)	(1.57 − 2.90)	(41.52 − 63.45)	(33.95 − 66.97)	(45.42 − 74.29)
Bangladesh	12.74	15.27	10.35	7.93	9.15	6.74	243.46	299.85	190.56
	(11.31 − 14.63)	(13.49 − 17.39)	(8.97 − 11.96)	(4.69 − 13.16)	(3.86 − 19.57)	(4.34 − 12.00)	(154.02 − 386.81)	(147.80 − 589.48)	(128.77 − 302)
Barbados	10.24	10.01	10.49	4.69	4.96	4.49	144.16	148.79	140.18
	(9.21 − 11.50)	(8.93 − 11.28)	(9.30 − 11.94)	(3.74 − 5.82)	(3.90 − 6.24)	(3.54 − 5.60)	(114.34 − 179.24)	(117.25 − 189.32)	(111.44 − 176.76)
Belarus	7.36	8.06	6.70	3.93	5.27	2.96	123.73	164.45	90.30
	(6.57 − 8.19)	(7.17 − 9.08)	(5.93 − 7.57)	(3.19 − 4.68)	(4.32 − 6.38)	(2.40 − 3.50)	(101.18 − 146.64)	(133.10 − 197.23)	(74.37 − 106.39)
Belgium	5.72	5.30	6.20	2.31	2.39	2.28	67.19	65.12	69.69
	(5.02 − 6.53)	(4.68 − 5.96)	(5.35 − 7.16)	(2.09 − 2.49)	(2.18 − 2.59)	(2.00 − 2.55)	(62.95 − 72.38)	(60.39 − 70.30)	(63.87 − 76.43)
Belize	8.43	7.51	9.34	2.73	2.52	2.94	92.36	86.10	98.71
	(7.49 − 9.44)	(6.65 − 8.42)	(8.27 − 10.56)	(2.39 − 3.10)	(2.16 − 2.92)	(2.57 − 3.31)	(81.22 − 103.89)	(74.20 − 99.57)	(87.08 − 111.53)
Benin	5.41	6.20	4.72	2.60	2.70	2.49	84.74	92.30	77.35
	(4.70 − 6.35)	(5.37 − 7.15)	(3.99 − 5.59)	(1.22 − 6.16)	(0.73 − 10.08)	(1.41 − 5.60)	(44.30 − 183.61)	(33.70 − 303.30)	(48.79 − 155.28)

Bermuda	8.64 (7.54 − 9.71)	8.34 (7.30 − 9.28)	9.04 (7.73 − 10.38)	2.24 (1.89 − 2.70)	3.14 (2.65 − 3.80)	1.51 (1.24 − 1.90)	72.39 (61.63 − 86.07)	91.28 (76.77 − 108.23)	55.76 (46.52 − 66.15)
Bhutan	7.64 (6.68 − 8.71)	7.89 (6.80 − 9.05)	7.38 (6.48 − 8.43)	3.68 (2.44 − 5.58)	3.66 (1.90 − 6.88)	3.70 (2.38 − 5.66)	114.23 (78.27 − 170.02)	114.23 (64.30 − 204.19)	114.16 (76.21 − 170.24)
Bolivia (Plurinational State of Bolivia)	14.43 (12.86 − 16.20)	12.02 (10.55 − 13.52)	16.71 (14.80 − 19.03)	9.70 (6.98 − 13.63)	7.12 (4.65 − 10.17)	11.98 (8.02 − 17.33)	300.13 (219.35 − 411.66)	234.42 (162.75 − 325.88)	359.90 (249.22 − 515.21)
Bosnia and Herzegovina	7.73 (6.93 − 8.59)	7.57 (6.78 − 8.46)	7.92 (7.05 − 8.84)	3.25 (2.38 − 4.21)	3.19 (2.14 − 4.53)	3.28 (2.35 − 4.33)	102.57 (77.01 − 130.54)	100.70 (69.44 − 140.09)	103.86 (77.63 − 133.43)
Botswana	5.86 (5.12 − 6.74)	6.29 (5.44 − 7.32)	5.58 (4.84 − 6.49)	1.34 (0.93 − 2.21)	1.55 (0.93 − 3.01)	1.17 (0.81 − 2.18)	46.75 (33.73 − 72.24)	53.35 (34.98 − 101.50)	40.68 (30.67 − 68.33)
Brazil	10.20 (8.84 − 11.81)	8.64 (7.47 − 9.97)	11.65 (10.06 − 13.57)	5.45 (5.12 − 5.68)	4.62 (4.36 − 4.87)	6.19 (5.72 − 6.53)	177.61 (169.76 − 184.33)	147.19 (140.13 − 154.67)	204.89 (194.17 − 214.64)
Brunei Darussalam	15.60 (13.78 − 17.87)	12.43 (10.70 − 14.52)	18.34 (16.04 − 21.30)	7.00 (5.73 − 8.50)	5.57 (4.20 − 7.46)	8.23 (6.41 − 10.28)	224.11 (188.53 − 267.80)	196.73 (152.17 − 259.19)	247.95 (201.13 − 299.61)
Bulgaria	8.32 (7.54 − 9.13)	8.82 (7.87 − 9.81)	7.96 (7.18 − 8.74)	5.09 (4.38 − 5.86)	5.66 (4.82 − 6.58)	4.62 (3.99 − 5.36)	154.16 (132.14 − 178.07)	169.73 (144.36 − 198.67)	139.96 (119.41 − 161.15)
Burkina Faso	5.00 (4.31 − 5.83)	5.68 (4.87 − 6.66)	4.42 (3.76 − 5.23)	2.25 (0.82 − 5.81)	3.08 (0.71 − 10.68)	1.51 (0.79 − 3.70)	76.70 (34.78 − 176.90)	103.14 (33.48 − 314.74)	52.56 (33.08 − 108.35)
Burundi	7.99 (6.86 − 9.49)	9.39 (7.97 − 11.15)	6.58 (5.58 − 7.87)	3.76 (0.85 − 13.92)	5.10 (0.68 − 24.70)	2.41 (0.93 − 8.27)	119.38 (37.30 − 389.51)	155.25 (32.85 − 663.13)	81.13 (39.71 − 228.46)

Côte d'Ivoire	5.07 (4.37 − 5.84)	5.68 (4.88 − 6.55)	4.43 (3.75 − 5.21)	2.45 (1.19 − 5.34)	2.73 (0.92 − 8.62)	2.12 (1.31 − 4.47)	87.78 (48.28 − 175.32)	102.29 (43.34 − 278.10)	71.25 (47.95 − 133.71)
Cabo Verde	5.18 (4.36 − 6.08)	5.58 (4.77 − 6.47)	4.88 (3.99 − 5.82)	1.66 (0.81 − 3.81)	1.95 (0.70 − 6.69)	1.36 (0.74 − 3.37)	62.53 (36.17 − 135.13)	74.46 (32.71 − 220.39)	49.99 (32.90 − 103.76)
Cambodia	10.80 (9.55 − 12.18)	10.34 (9.09 − 11.68)	11.20 (9.91 − 12.81)	5.88 (4.20 − 10.09)	5.29 (3.39 − 9.61)	6.19 (4.32 − 10.85)	165.54 (122.80 − 242.74)	155.18 (105.68 − 255.96)	170.76 (124.35 − 257.70)
Cameroon	5.27 (4.56 − 6.14)	5.99 (5.19 − 7.02)	4.61 (3.91 − 5.38)	2.80 (1.27 − 6.19)	3.26 (0.99 − 9.72)	2.36 (1.30 − 5.32)	97.33 (48.64 − 195.34)	116.35 (44.28 − 310.22)	78.64 (48.18 − 153.59)
Canada	7.03 (6.11 − 8.04)	5.90 (5.15 − 6.75)	8.14 (6.93 − 9.45)	2.36 (2.13 − 2.55)	2.24 (2.02 − 2.45)	2.47 (2.18 − 2.71)	76.13 (69.97 − 82.49)	69.67 (63.95 − 75.54)	82.01 (74.43 − 89.56)
Central African Republic	7.50 (6.45 − 8.84)	8.75 (7.53 − 10.27)	6.54 (5.58 − 7.78)	4.49 (1.55 − 11.58)	5.92 (1.24 − 19.89)	3.40 (1.44 − 9.43)	141.68 (57.82 − 314.50)	183.26 (51.72 − 529.94)	105.63 (51.92 − 243.45)
Chad	5.67 (4.90 − 6.60)	6.41 (5.53 − 7.44)	4.91 (4.22 − 5.78)	3.45 (1.34 − 8.47)	3.80 (0.93 − 13.40)	3.10 (1.67 − 6.79)	117.76 (52.85 − 264.50)	135.53 (45.26 − 428.69)	100.31 (59.08 − 199.65)
Chile	9.61 (8.46 − 10.82)	7.66 (6.61 − 8.67)	11.40 (9.98 − 13.06)	3.79 (3.50 − 4.08)	3.66 (3.36 − 4.00)	3.91 (3.54 − 4.27)	120.38 (112.85 − 129.21)	114.32 (104.88 − 123.67)	125.87 (116.27 − 136.73)
China	7.81 (6.88 − 8.95)	8.78 (7.67 − 10.15)	6.95 (6.06 − 7.92)	4.72 (3.45 − 5.95)	5.68 (3.31 − 7.93)	4.02 (2.76 − 5.43)	115.49 (86.86 − 142.50)	133.36 (84.49 − 181.13)	99.70 (73.36 − 130.60)
Colombia	13.97 (12.55 − 15.71)	11.80 (10.43 − 13.42)	15.85 (14.02 − 17.98)	6.09 (5.08 − 7.19)	5.43 (4.45 − 6.53)	6.62 (5.54 − 7.80)	184.51 (155.49 − 217.65)	166.35 (138.46 − 198.16)	199.23 (168.05 − 232.34)
Comoros	7.36 (6.39 − 8.62)	8.58 (7.43 − 10.11)	6.27 (5.37 − 7.45)	2.63 (1.07 − 6.33)	3.22 (0.75 − 10.95)	2.12 (1.07 − 5.20)	88.71 (43.07 − 188.52)	105.73 (33.82 − 311.80)	72.99 (44.57 − 152.96)

Congo	6.17 (5.35 − 7.09)	6.72 (5.77 − 7.74)	5.71 (4.91 − 6.68)	2.69 (1.45 − 5.27)	2.69 (0.96 − 7.22)	2.70 (1.69 − 5.63)	87.67 (51.87 − 155.51)	87.63 (38.14 − 208.30)	87.83 (57.82 − 166.36)
Cook Islands	9.90 (8.77 − 11.05)	9.57 (8.32 − 10.91)	10.21 (8.99 − 11.52)	2.83 (1.87 − 3.90)	2.68 (1.62 − 3.93)	2.92 (1.78 − 4.48)	113.54 (86.33 − 144.32)	122.29 (83.55 − 167.92)	105.96 (77.35 − 145.18)
Costa Rica	10.25 (8.95 − 11.66)	9.07 (7.91 − 10.48)	11.32 (9.73 − 12.96)	3.65 (3.16 − 4.04)	3.64 (3.15 − 4.05)	3.65 (3.15 − 4.13)	117.80 (104.96 − 129.08)	116.54 (103.00 − 129.22)	118.69 (104.38 − 132.05)
Croatia	5.93 (5.34 − 6.60)	5.72 (5.06 − 6.44)	6.13 (5.53 − 6.88)	3.39 (2.95 − 3.91)	3.48 (3.01 − 4.03)	3.29 (2.83 − 3.78)	93.44 (82.06 − 106.54)	95.56 (83.67 − 109.43)	91.18 (78.65 − 105.33)
Cuba	8.44 (7.42 − 9.54)	7.31 (6.43 − 8.25)	9.56 (8.35 − 11.02)	2.79 (2.39 − 3.17)	2.61 (2.22 − 3.04)	2.94 (2.50 − 3.40)	91.85 (80.29 − 103.71)	85.42 (73.37 − 98.66)	97.56 (84.53 − 112.17)
Cyprus	7.01 (6.13 − 7.98)	6.71 (5.82 − 7.71)	7.25 (6.26 − 8.33)	2.65 (2.11 − 3.36)	2.44 (1.78 − 3.20)	2.74 (2.03 − 3.55)	68.28 (56.81 − 81.96)	66.26 (51.35 − 84.27)	69.20 (56.59 − 84.39)
Czechia	6.11 (5.35 − 6.81)	6.15 (5.39 − 6.90)	6.10 (5.30 − 6.89)	2.30 (2.01 − 2.64)	2.30 (1.99 − 2.63)	2.31 (2.00 − 2.66)	73.74 (64.69 − 84.50)	70.61 (60.87 − 79.99)	76.91 (67.21 − 88.84)
Democratic People's Republic of Korea	11.65 (10.29 − 13.04)	13.34 (11.67 − 15.12)	10.79 (9.51 − 12.19)	7.72 (4.65 − 14.04)	8.76 (4.46 − 22.79)	7.08 (4.41 − 11.88)	216.33 (136.18 − 370.31)	238.75 (132.87 − 541.67)	198.52 (129.22 − 309.49)
Democratic Republic of the Congo	7.75 (6.67 − 9.12)	9.28 (7.93 − 10.99)	6.47 (5.51 − 7.72)	3.30 (1.08 − 10.89)	4.25 (0.68 − 20.46)	2.60 (1.22 − 7.31)	102.20 (42.99 − 302.44)	126.40 (31.62 − 540.51)	81.11 (44.90 − 198.66)
Denmark	7.42 (6.51 − 8.47)	6.64 (5.65 − 7.60)	8.17 (7.10 − 9.46)	2.73 (2.47 − 2.95)	2.59 (2.32 − 2.83)	2.85 (2.53 − 3.12)	76.66 (70.56 − 81.99)	71.00 (64.50 − 77.11)	82.03 (75.23 − 89.18)

Djibouti	7.49<br>(6.45 − 8.73)	8.77<br>(7.57 − 10.24)	6.09<br>(5.14 − 7.32)	2.83<br>(1.15 − 6.26)	3.69<br>(1.18 − 10.42)	1.88<br>(0.94 − 4.34)	94.58<br>(44.72 − 194.76)	121.89<br>(46.59 − 308.79)	62.85<br>(37.71 − 127.25)
Dominica	8.99<br>(7.91 − 10.12)	8.90<br>(7.83 − 10.06)	9.31<br>(8.08 − 10.59)	4.42<br>(3.56 − 5.39)	4.97<br>(3.75 − 6.49)	4.00<br>(3.13 − 5.04)	136.67<br>(113.11 − 166.88)	146.93<br>(113.61 − 188.01)	127.09<br>(100.80 − 158.85)
Dominican Republic	10.97<br>(9.68 − 12.47)	8.62<br>(7.57 − 9.81)	13.30<br>(11.67 − 15.39)	4.04<br>(2.98 − 5.27)	3.32<br>(2.28 − 4.79)	4.72<br>(3.32 − 6.51)	146.74<br>(114.48 − 185.95)	122.13<br>(86.09 − 167.31)	170.70<br>(126.51 − 229.28)
Ecuador	14.98<br>(13.58 − 16.91)	13.47<br>(12.10 − 15.34)	16.38<br>(14.53 − 18.74)	7.69<br>(6.14 − 9.47)	7.23<br>(5.71 − 9.28)	8.09<br>(6.40 − 9.94)	234.98<br>(190.75 − 288.98)	226.17<br>(181.86 − 289.46)	242.30<br>(196.25 − 295.55)
Egypt	5.52<br>(4.84 − 6.20)	5.06<br>(4.44 − 5.71)	6.00<br>(5.23 − 6.80)	2.99<br>(2.05 − 4.36)	3.00<br>(1.86 − 4.76)	3.17<br>(1.82 − 5.56)	83.75<br>(60.65 − 119.58)	86.60<br>(57.93 − 138.54)	82.63<br>(53.12 − 130.84)
El Salvador	9.75<br>(8.67 − 10.93)	8.26<br>(7.28 − 9.25)	10.88<br>(9.63 − 12.39)	3.37<br>(2.57 − 4.34)	2.86<br>(1.90 − 3.78)	3.73<br>(2.70 − 4.94)	118.51<br>(93.68 − 147.91)	105.43<br>(70.53 − 137.47)	127.57<br>(97.29 − 163.78)
Equatorial Guinea	5.30<br>(4.51 − 6.21)	5.83<br>(4.94 − 6.79)	4.82<br>(4.06 − 5.75)	1.56<br>(0.78 − 2.63)	1.65<br>(0.65 − 3.58)	1.48<br>(0.66 − 3.12)	54.32<br>(30.08 − 83.95)	56.97<br>(27.94 − 109.37)	51.60<br>(28.03 − 98.49)
Eritrea	8.20<br>(7.10 − 9.67)	9.44<br>(8.11 − 11.07)	7.21<br>(6.18 − 8.66)	3.84<br>(1.45 − 9.04)	5.51<br>(1.24 − 18.03)	2.69<br>(1.44 − 6.61)	127.23<br>(54.59 − 272.81)	174.16<br>(50.64 − 496.55)	88.32<br>(52.48 − 186.04)
Estonia	6.36<br>(5.57 − 7.12)	6.81<br>(5.96 − 7.62)	5.98<br>(5.17 − 6.78)	2.41<br>(2.08 − 2.71)	3.47<br>(3.03 − 3.97)	1.65<br>(1.36 − 1.88)	70.24<br>(61.16 − 79.30)	96.99<br>(85.21 − 110.54)	47.91<br>(41.36 − 54.51)
Eswatini	5.57<br>(4.90 − 6.44)	6.40<br>(5.62 − 7.37)	5.05<br>(4.39 − 5.89)	2.32<br>(1.50 − 3.70)	2.72<br>(1.54 − 4.84)	2.00<br>(1.18 − 3.30)	75.24<br>(48.72 − 118.52)	90.16<br>(53.54 − 155.93)	62.46<br>(39.88 − 97.75)

Ethiopia	6.55 (5.60 − 7.73)	8.02 (6.86 − 9.42)	5.05 (4.32 − 5.96)	2.15 (0.70 − 6.65)	2.88 (0.58 − 11.81)	1.41 (0.67 − 3.87)	70.39 (29.41 − 189.81)	90.73 (26.69 − 327.23)	49.81 (28.82 − 117.11)
Fiji	16.55 (15.02 − 18.22)	9.66 (8.62 − 10.77)	22.05 (19.96 − 24.56)	7.83 (5.97 − 10.32)	5.30 (4.01 − 7.21)	9.37 (6.84 − 12.62)	254.88 (198.88 − 321.88)	205.23 (158.32 − 268.68)	292.81 (218.47 − 387.12)
Finland	10.27 (9.00 − 11.74)	9.42 (8.25 − 10.78)	11.07 (9.58 − 12.73)	3.35 (3.00 − 3.61)	3.33 (3.04 − 3.57)	3.33 (2.94 − 3.65)	110.60 (102.95 − 119.05)	110.57 (102.31 − 119.66)	109.92 (101.01 − 120.35)
France	6.48 (5.75 − 7.26)	6.29 (5.57 − 7.07)	6.68 (5.87 − 7.66)	2.09 (1.89 − 2.25)	2.22 (2.04 − 2.38)	1.98 (1.72 − 2.18)	66.55 (61.47 − 71.66)	69.99 (64.37 − 75.65)	63.21 (57.62 − 69.70)
Gabon	5.96 (5.18 − 6.97)	6.65 (5.71 − 7.71)	5.41 (4.55 − 6.43)	1.95 (1.16 − 3.29)	2.37 (1.14 − 4.97)	1.61 (0.84 − 2.97)	64.90 (41.12 − 105.68)	77.18 (41.40 − 148.76)	53.74 (33.70 − 91.22)
Gambia	5.27 (4.56 − 6.15)	6.00 (5.13 − 6.96)	4.62 (3.93 − 5.42)	3.05 (1.31 − 7.59)	3.29 (0.88 − 12.50)	2.80 (1.48 − 6.54)	105.25 (51.58 − 242.97)	119.66 (42.19 − 402.38)	91.09 (53.03 − 186.17)
Georgia	16.12 (14.71 − 17.80)	19.18 (17.22 − 21.32)	13.29 (11.92 − 15.03)	8.83 (7.26 − 10.49)	11.02 (8.85 − 13.37)	7.08 (5.83 − 8.37)	244.00 (201.68 − 286.44)	319.30 (260.34 − 388.32)	177.12 (146.67 − 206.65)
Germany	6.30 (5.42 − 7.26)	5.71 (4.97 − 6.57)	6.90 (5.85 − 8.08)	2.17 (1.94 − 2.34)	2.10 (1.91 − 2.28)	2.27 (1.98 − 2.48)	69.11 (64.47 − 74.57)	63.91 (59.08 − 68.84)	74.43 (68.18 − 80.98)
Ghana	6.08 (5.21 − 7.10)	7.13 (6.11 − 8.21)	5.22 (4.38 − 6.16)	3.19 (1.70 − 6.19)	3.54 (1.28 − 10.25)	2.85 (1.73 − 5.55)	113.34 (64.95 − 211.59)	134.78 (57.36 − 341.66)	93.91 (62.08 − 168.83)
Greece	7.20 (6.30 − 8.23)	6.79 (5.92 − 7.71)	7.59 (6.51 − 8.77)	2.86 (2.60 − 3.05)	3.06 (2.79 − 3.30)	2.69 (2.37 − 2.93)	86.72 (80.58 − 93.76)	93.60 (85.63 − 101.08)	80.30 (73.14 − 88.07)
Greenland	11.62 (10.55 − 12.93)	10.39 (9.41 − 11.54)	13.29 (12.04 − 14.81)	7.53 (5.65 − 9.37)	6.05 (3.30 − 8.20)	9.03 (7.11 − 11.59)	231.20 (182.48 − 279.81)	183.56 (113.00 − 239.09)	284.02 (228.51 − 354.89)

Grenada	10.27<br>(9.19 − 11.49)	9.89<br>(8.85 − 11.05)	11.60<br>(10.34 − 13.22)	4.67<br>(4.08 − 5.27)	4.76<br>(4.12 − 5.42)	4.82<br>(4.17 − 5.50)	146.31<br>(126.09 − 165.44)	140.85<br>(120.74 − 159.74)	154.24<br>(133.98 − 176.63)
Guam	10.11<br>(8.99 − 11.25)	9.94<br>(8.86 − 11.08)	10.13<br>(8.92 − 11.40)	2.95<br>(2.46 − 3.46)	3.04<br>(2.38 − 3.97)	2.80<br>(2.19 − 3.46)	136.77<br>(117.70 − 159.68)	150.35<br>(122.41 − 186.29)	121.31<br>(101.23 − 144.34)
Guatemala	8.94<br>(8.00 − 10.10)	8.21<br>(7.30 − 9.30)	9.58<br>(8.45 − 10.92)	2.53<br>(2.16 − 2.91)	2.53<br>(2.09 − 2.96)	2.53<br>(2.13 − 2.93)	93.75<br>(80.59 − 107.15)	92.76<br>(78.44 − 107.43)	94.36<br>(80.70 − 108.20)
Guinea	6.05<br>(5.19 − 7.05)	7.19<br>(6.20 − 8.44)	4.99<br>(4.25 − 5.79)	3.04<br>(1.32 − 6.88)	3.21<br>(0.85 − 10.70)	2.90<br>(1.65 − 7.23)	107.15<br>(55.14 − 216.70)	119.50<br>(45.79 − 334.98)	96.41<br>(61.18 − 201.26)
Guinea-Bissau	5.68<br>(4.91 − 6.62)	6.59<br>(5.64 − 7.75)	4.92<br>(4.21 − 5.77)	4.41<br>(1.85 − 9.87)	4.89<br>(1.20 − 16.49)	3.98<br>(2.20 − 9.45)	151.44<br>(70.21 − 320.57)	175.81<br>(53.99 − 539.94)	129.49<br>(75.22 − 268.57)
Guyana	11.29<br>(10.21 − 12.57)	10.50<br>(9.44 − 11.70)	12.09<br>(10.81 − 13.76)	5.51<br>(4.26 − 6.96)	6.05<br>(4.61 − 7.69)	5.03<br>(3.83 − 6.40)	191.24<br>(147.74 − 242.52)	209.36<br>(161.29 − 266.13)	174.22<br>(133.18 − 221.81)
Haiti	14.75<br>(13.08 − 16.88)	13.03<br>(11.53 − 14.96)	16.30<br>(14.32 − 18.64)	12.78<br>(6.59 − 20.85)	10.95<br>(4.42 − 22.25)	14.45<br>(4.97 − 24.62)	434.98<br>(237.15 − 658.46)	352.07<br>(168.36 − 635.23)	511.90<br>(180.91 − 806.50)
Honduras	12.35<br>(11.06 − 13.91)	10.77<br>(9.56 − 12.22)	13.73<br>(12.17 − 15.66)	11.86<br>(8.47 − 15.42)	9.13<br>(5.67 − 13.37)	14.22<br>(9.44 − 19.38)	340.96<br>(235.34 − 447.31)	263.84<br>(167.08 − 390.44)	407.97<br>(268.02 − 562.14)
Hungary	5.99<br>(5.33 − 6.64)	5.68<br>(5.03 − 6.37)	6.33<br>(5.61 − 7.12)	2.17<br>(1.84 − 2.50)	2.15<br>(1.84 − 2.46)	2.18<br>(1.84 − 2.55)	75.62<br>(65.06 − 86.61)	72.49<br>(63.06 − 82.50)	78.50<br>(66.46 − 91.25)
Iceland	7.24<br>(6.34 − 8.32)	7.10<br>(6.17 − 8.22)	7.40<br>(6.37 − 8.58)	2.05<br>(1.76 − 2.27)	1.87<br>(1.64 − 2.10)	2.19<br>(1.85 − 2.46)	62.92<br>(56.80 − 69.61)	59.92<br>(53.82 − 66.50)	65.54<br>(58.33 − 73.23)
India	7.70<br>(6.65 − 8.95)	8.25<br>(7.11 − 9.57)	7.19<br>(6.21 − 8.37)	3.95<br>(2.73 − 5.50)	4.55<br>(2.18 − 7.46)	3.42<br>(2.54 − 4.73)	125.93<br>(91.47 − 167.76)	142.61<br>(78.43 − 221.67)	109.71<br>(84.38 − 146.99)

Indonesia	10.94<br>(9.54 − 12.64)	10.66<br>(9.26 − 12.24)	11.26<br>(9.81 − 13.06)	6.53<br>(4.72 − 10.63)	5.58<br>(3.03 − 10.30)	7.27<br>(4.56 − 12.88)	198.84<br>(154.02 − 287.20)	182.64<br>(115.14 − 298.94)	212.18<br>(142.83 − 317.26)
Iran (Islamic Republic of Iran)	4.76<br>(4.15 − 5.41)	4.54<br>(3.95 − 5.16)	4.98<br>(4.31 − 5.69)	1.40<br>(1.05 − 1.69)	1.30<br>(0.88 − 1.74)	1.50<br>(1.09 − 1.83)	40.90<br>(34.14 − 48.29)	39.05<br>(29.87 − 50.44)	42.79<br>(35.07 − 51.96)
Iraq	5.11<br>(4.47 − 5.76)	5.04<br>(4.43 − 5.68)	5.30<br>(4.58 − 6.06)	2.36<br>(1.69 − 3.07)	2.44<br>(1.60 − 3.38)	2.30<br>(1.58 − 3.21)	74.78<br>(56.72 − 97.71)	73.59<br>(52.78 − 99.14)	76.47<br>(56.60 − 101.96)
Ireland	7.53<br>(6.64 − 8.52)	6.51<br>(5.64 − 7.41)	8.49<br>(7.40 − 9.79)	2.68<br>(2.36 − 2.94)	2.57<br>(2.29 − 2.86)	2.76<br>(2.35 − 3.09)	74.87<br>(68.89 − 81.33)	71.17<br>(64.98 − 78.17)	78.06<br>(69.12 − 86.04)
Israel	5.65<br>(4.84 − 6.54)	5.49<br>(4.68 − 6.32)	5.83<br>(4.90 − 6.84)	1.33<br>(1.18 − 1.45)	1.71<br>(1.53 − 1.89)	1.02<br>(0.88 − 1.13)	41.16<br>(37.50 − 44.92)	48.03<br>(43.66 − 52.58)	35.16<br>(31.26 − 39.42)
Italy	4.84<br>(4.26 − 5.39)	4.48<br>(3.95 − 5.02)	5.20<br>(4.57 − 5.87)	1.87<br>(1.66 − 2.01)	1.94<br>(1.78 − 2.05)	1.83<br>(1.59 − 1.99)	52.22<br>(48.49 − 55.95)	51.57<br>(48.37 − 54.88)	53.00<br>(48.58 − 57.31)
Jamaica	11.67<br>(10.55 − 13.08)	9.67<br>(8.57 − 11.00)	13.63<br>(12.09 − 15.49)	4.75<br>(3.68 − 6.04)	4.23<br>(3.16 − 5.51)	5.25<br>(4.07 − 6.67)	149.99<br>(116.73 − 190.13)	126.71<br>(97.23 − 166.70)	172.53<br>(133.38 − 218.04)
Japan	15.83<br>(13.71 − 18.40)	13.59<br>(11.68 − 15.77)	17.76<br>(15.35 − 20.76)	4.21<br>(3.76 − 4.49)	4.18<br>(3.99 − 4.35)	4.06<br>(3.42 − 4.45)	150.89<br>(138.85 − 162.62)	156.53<br>(146.64 − 166.62)	142.30<br>(127.94 − 156.17)
Jordan	4.69<br>(4.03 − 5.37)	4.80<br>(4.16 − 5.55)	4.56<br>(3.81 − 5.33)	0.51<br>(0.36 − 0.65)	0.47<br>(0.29 − 0.68)	0.55<br>(0.37 − 0.74)	24.18<br>(19.26 − 29.41)	23.26<br>(17.12 − 30.18)	25.24<br>(19.93 − 32.09)
Kazakhstan	10.15<br>(9.12 − 11.28)	12.05<br>(10.73 − 13.66)	8.64<br>(7.68 − 9.66)	6.46<br>(5.60 − 7.37)	7.46<br>(6.41 − 8.64)	5.65<br>(4.84 − 6.53)	169.52<br>(145.47 − 193.34)	203.46<br>(172.66 − 236.42)	141.10<br>(121.09 − 163.10)
Kenya	6.46<br>(5.58 − 7.60)	7.81<br>(6.76 − 9.11)	5.34<br>(4.60 − 6.26)	2.07<br>(0.93 − 5.05)	2.70<br>(0.89 − 9.07)	1.60<br>(0.82 − 4.50)	68.12<br>(35.50 − 148.42)	84.38<br>(34.00 − 243.07)	53.90<br>(32.92 − 134.44)

Kiribati	22.01 (19.58 − 24.58)	22.43 (19.14 − 26.07)	20.95 (18.62 − 23.77)	9.04 (6.61 − 13.07)	11.66 (7.21 − 20.35)	6.54 (4.67 − 8.91)	349.55 (258.11 − 479.78)	485.74 (327.54 − 778.30)	223.08 (168.27 − 288.73)
Kuwait	5.55 (4.76 − 6.31)	5.27 (4.57 − 5.98)	5.83 (4.99 − 6.69)	0.51 (0.42 − 0.61)	0.63 (0.51 − 0.78)	0.34 (0.29 − 0.40)	25.93 (21.96 − 30.34)	27.85 (23.38 − 32.96)	23.69 (19.34 − 28.70)
Kyrgyzstan	9.56 (8.55 − 10.68)	11.08 (9.85 − 12.55)	8.30 (7.40 − 9.35)	6.13 (5.19 − 7.21)	8.19 (6.90 − 9.75)	4.64 (3.87 − 5.56)	186.36 (156.23 − 220.95)	242.92 (200.40 − 288.78)	139.54 (116.59 − 166.50)
Lao People’s Democratic Republic	9.87 (8.74 − 11.12)	9.89 (8.77 − 11.26)	9.88 (8.69 − 11.20)	5.68 (4.03 − 8.68)	5.33 (2.87 − 9.88)	5.93 (3.58 − 10.07)	183.57 (135.18 − 261.22)	180.91 (110.08 − 293.35)	184.74 (120.21 − 273.55)
Latvia	7.23 (6.39 − 8.05)	7.92 (6.98 − 8.86)	6.65 (5.80 − 7.58)	3.14 (2.75 − 3.56)	4.04 (3.44 − 4.67)	2.51 (2.19 − 2.86)	93.12 (81.10 − 106.48)	118.05 (100.67 − 137.63)	72.22 (62.47 − 82.18)
Lebanon	5.55 (4.80 − 6.29)	5.09 (4.38 − 5.79)	6.00 (5.19 − 6.81)	2.06 (1.62 − 2.58)	2.36 (1.71 − 3.17)	1.79 (1.32 − 2.38)	61.49 (51.22 − 73.63)	67.07 (50.60 − 86.05)	56.37 (45.83 − 70.26)
Lesotho	6.46 (5.64 − 7.39)	6.89 (5.96 − 7.88)	6.28 (5.45 − 7.31)	3.49 (2.08 − 5.80)	4.44 (2.32 − 8.09)	2.86 (1.62 − 4.98)	106.98 (65.68 − 176.90)	135.16 (73.95 − 243.24)	85.57 (50.33 − 141.88)
Liberia	5.51 (4.73 − 6.47)	6.32 (5.35 − 7.50)	4.66 (4.01 − 5.48)	2.91 (0.97 − 9.10)	2.99 (0.47 − 14.83)	2.82 (1.40 − 8.63)	101.07 (41.10 − 284.17)	108.11 (27.86 − 449.31)	93.45 (51.21 − 235.23)
Libya	5.09 (4.47 − 5.76)	4.73 (4.11 − 5.39)	5.48 (4.77 − 6.30)	2.15 (1.29 − 3.35)	1.71 (0.94 − 3.25)	2.61 (1.37 − 4.15)	79.74 (49.55 − 120.40)	65.15 (40.48 − 110.35)	94.98 (51.39 − 144.08)
Lithuania	6.75 (5.96 − 7.56)	7.02 (6.21 − 7.91)	6.51 (5.66 − 7.35)	3.03 (2.64 − 3.41)	3.79 (3.26 − 4.35)	2.47 (2.12 − 2.79)	91.73 (80.30 − 102.93)	113.05 (97.61 − 129.04)	73.66 (63.19 − 83.74)
Luxembourg	5.22 (4.50 – 6.00)	5.12 (4.43 − 5.88)	5.38 (4.57 − 6.24)	1.58 (1.41 − 1.76)	1.68 (1.48 − 1.89)	1.54 (1.35 − 1.73)	45.54 (41.35 − 50.45)	45.26 (40.70 − 50.39)	46.31 (41.65 − 51.55)

Madagascar	9.87<br>(8.43 − 11.47)	11.85<br>(10.15 − 13.86)	8.04<br>(6.82 − 9.56)	5.37<br>(1.86 − 15.13)	6.89<br>(1.52 − 26.77)	4.02<br>(1.96 − 9.86)	182.17<br>(73.64 − 469.84)	231.59<br>(67.03 − 815.27)	136.05<br>(76.23 − 309.78)
Malawi	6.95<br>(5.91 − 8.15)	8.63<br>(7.35 − 10.26)	5.58<br>(4.69 − 6.58)	3.51<br>(1.19 − 10.68)	4.62<br>(0.98 − 20.55)	2.62<br>(1.12 − 7.85)	114.81<br>(45.32 − 308.47)	150.65<br>(41.30 − 585.91)	83.34<br>(42.11 − 217.98)
Malaysia	9.05<br>(8.05 − 10.12)	8.14<br>(7.22 − 9.19)	9.97<br>(8.79 − 11.20)	3.50<br>(2.67 − 4.28)	2.69<br>(1.66 − 3.65)	4.34<br>(3.18 − 5.70)	107.43<br>(84.91 − 127.31)	91.99<br>(64.86 − 120.29)	122.71<br>(96.03 − 150.78)
Maldives	8.55<br>(7.54 − 9.73)	8.40<br>(7.33 − 9.68)	8.64<br>(7.57 − 9.80)	2.64<br>(2.05 − 3.31)	2.38<br>(1.68 − 3.29)	2.88<br>(2.19 − 3.73)	79.13<br>(63.73 − 97.86)	75.77<br>(57.92 − 103.89)	80.91<br>(64.85 − 101.62)
Mali	5.19<br>(4.46 − 6.02)	6.18<br>(5.28 − 7.19)	4.18<br>(3.49 − 4.90)	2.48<br>(1.11 − 5.53)	2.28<br>(0.62 − 7.80)	2.70<br>(1.50 − 5.63)	87.74<br>(47.00 − 178.27)	84.54<br>(33.14 − 254.34)	91.18<br>(56.32 − 175.31)
Malta	6.14<br>(5.27 − 7.10)	5.66<br>(4.86 − 6.52)	6.66<br>(5.66 − 7.77)	1.18<br>(1.05 − 1.32)	1.16<br>(1.04 − 1.31)	1.21<br>(1.06 − 1.36)	45.19<br>(40.40 − 50.63)	42.76<br>(38.27 − 48.23)	47.59<br>(41.66 − 53.81)
Marshall Islands	20.18<br>(18.03 − 22.51)	18.41<br>(16.27 − 20.78)	22.00<br>(19.52 − 24.75)	12.31<br>(7.83 − 17.85)	12.06<br>(6.26 − 20.18)	12.64<br>(6.69 − 18.94)	431.34<br>(286.73 − 619.46)	469.86<br>(261.01 − 738.53)	391.72<br>(222.68 − 578.96)
Mauritania	5.18<br>(4.40 − 6.09)	5.76<br>(4.92 − 6.81)	4.61<br>(3.88 − 5.46)	2.25<br>(0.98 − 5.16)	2.11<br>(0.58 − 8.13)	2.39<br>(1.31 − 5.47)	77.82<br>(39.96 − 164.89)	78.16<br>(30.11 − 256.56)	77.90<br>(47.34 − 159.35)
Mauritius	10.76<br>(9.55 − 12.13)	10.37<br>(9.22 − 11.69)	11.16<br>(9.77 − 12.74)	5.72<br>(5.27 − 6.09)	6.14<br>(5.68 − 6.58)	5.23<br>(4.73 − 5.64)	187.30<br>(171.59 − 199.49)	206.00<br>(188.34 − 221.29)	167.21<br>(152.04 − 181.06)
Mexico	9.82<br>(8.60 − 11.24)	8.71<br>(7.62 − 9.99)	10.82<br>(9.43 − 12.48)	4.04<br>(3.59 − 4.49)	3.95<br>(3.32 − 4.63)	4.10<br>(3.48 − 4.69)	130.47<br>(117.11 − 144.99)	132.76<br>(113.25 − 153.60)	127.76<br>(110.88 − 146.31)

Micronesia (Federated States of Micronesia)	19.75 (17.67 − 22.13)	17.95 (15.79 − 20.37)	20.80 (18.55 − 23.52)	11.87 (7.68 − 16.99)	11.59 (6.43 − 19.19)	11.46 (6.72 − 17.37)	406.22 (273.77 − 555.66)	451.81 (278.93 − 702.69)	349.75 (219.14 − 522.15)
Monaco	5.60 (4.82 − 6.42)	4.95 (4.21 − 5.73)	6.24 (5.33 − 7.17)	1.87 (1.34 − 2.55)	1.38 (0.85 − 1.91)	2.29 (1.51 − 3.28)	65.82 (49.14 − 88.01)	51.14 (35.04 − 68.92)	79.53 (55.75 − 112.94)
Mongolia	13.01 (11.61 − 14.64)	15.34 (13.61 − 17.37)	11.34 (10.03 − 12.81)	12.41 (9.07 − 16.05)	14.54 (9.03 − 21.18)	10.77 (7.81 − 14.27)	312.23 (232.02 − 397.20)	375.93 (240.63 − 544.06)	259.56 (195.67 − 342.32)
Montenegro	5.23 (4.58 − 5.88)	5.26 (4.58 − 5.95)	5.28 (4.59 − 5.98)	2.15 (1.54 − 2.89)	1.74 (1.06 − 2.70)	2.46 (1.60 − 3.50)	61.62 (47.52 − 78.33)	51.25 (35.88 − 73.48)	70.46 (50.93 − 95.15)
Morocco	6.87 (6.05 − 7.77)	6.71 (5.91 − 7.62)	7.07 (6.17 − 8.04)	3.43 (2.02 − 5.41)	2.96 (1.46 − 5.70)	3.89 (1.69 − 6.29)	97.77 (59.95 − 148.98)	78.97 (42.84 − 147.04)	116.54 (56.45 − 184.82)
Mozambique	8.46 (7.27 − 10.10)	10.77 (9.26 − 12.77)	6.60 (5.59 − 7.94)	5.38 (1.75 − 15.43)	8.23 (1.91 − 31.61)	3.14 (1.33 − 8.51)	174.18 (64.61 − 475.93)	265.87 (75.07 − 928.51)	96.80 (48.49 − 240.55)
Myanmar	9.48 (8.49 − 10.69)	10.46 (9.22 − 11.90)	8.69 (7.71 − 9.78)	5.89 (4.15 − 9.64)	6.83 (4.29 − 11.89)	5.08 (3.50 − 8.42)	191.83 (142.91 − 273.19)	229.52 (154.05 − 354.83)	158.06 (116.39 − 227.25)
Namibia	5.78 (5.05 − 6.68)	6.44 (5.59 − 7.52)	5.32 (4.56 − 6.19)	1.96 (1.33 − 3.12)	2.43 (1.51 − 4.36)	1.60 (1.03 − 2.89)	62.49 (43.55 − 94.93)	77.30 (49.83 − 134.34)	50.18 (34.69 − 86.16)
Nauru	16.13 (14.37 − 17.95)	14.84 (13.06 − 16.86)	17.03 (15.13 − 19.22)	12.87 (9.14 − 17.22)	10.46 (6.15 − 15.60)	14.56 (8.99 − 20.18)	476.38 (352.58 − 635.27)	438.93 (275.76 − 630.20)	501.68 (343.10 − 687.54)
Nepal	8.88 (7.71 − 10.34)	9.08 (7.86 − 10.53)	8.73 (7.48 − 10.12)	4.57 (2.67 − 7.23)	5.20 (2.19 − 10.50)	4.03 (2.40 − 7.55)	139.47 (88.10 − 211.95)	157.91 (78 − 307.09)	123.50 (80.69 − 203.53)

Netherlands	6.53<br>(5.68 − 7.43)	5.59<br>(4.85 − 6.36)	7.48<br>(6.48 − 8.65)	2.27<br>(2.03 − 2.45)	2.18<br>(1.98 − 2.38)	2.38<br>(2.08 − 2.60)	65.47<br>(60.53 − 70.34)	59.69<br>(55.13 − 64.83)	71.25<br>(65.24 − 77.90)
New Zealand	5.81<br>(5.07 − 6.60)	4.87<br>(4.22 − 5.59)	6.70<br>(5.85 − 7.59)	2.97<br>(2.68 − 3.21)	2.67<br>(2.47 − 2.88)	3.24<br>(2.86 − 3.55)	89.48<br>(82.69 − 95.65)	80.40<br>(74.82 − 86.36)	97.86<br>(89.34 − 105.74)
Nicaragua	10.04<br>(8.78 − 11.51)	8.49<br>(7.45 − 9.84)	11.40<br>(9.86 − 13.11)	2.75<br>(2.15 − 3.37)	2.31<br>(1.53 − 3.04)	3.11<br>(2.30 − 3.97)	96.00<br>(78.21 − 115.95)	81.50<br>(60.44 − 104.62)	108.10<br>(84.89 − 134.03)
Niger	5.83<br>(5.06 − 6.89)	6.68<br>(5.79 − 7.87)	5.03<br>(4.30 − 5.93)	2.95<br>(1.04 − 7.65)	3.38<br>(0.62 − 11.92)	2.54<br>(1.30 − 6.89)	94.89<br>(42.70 − 216.47)	109.40<br>(31.19 − 340.97)	81.32<br>(48.17 − 190.49)
Nigeria	4.46<br>(3.84 − 5.20)	4.87<br>(4.19 − 5.66)	4.10<br>(3.48 − 4.80)	1.55<br>(0.75 − 3.55)	1.81<br>(0.52 − 6.24)	1.34<br>(0.78 − 2.86)	55.39<br>(31.94 − 111.94)	67.16<br>(27.75 − 194.27)	45.27<br>(30.43 − 82.54)
Niue	13.10<br>(11.65 − 14.70)	11.63<br>(10.40 − 13.17)	14.06<br>(12.40 − 15.89)	7.37<br>(5.75 − 9.48)	6.33<br>(4.67 − 9.08)	7.91<br>(5.85 − 10.28)	298.12<br>(246.16 − 369.82)	285.45<br>(219.81 − 381.29)	305.83<br>(239.99 − 378.44)
North Macedonia	12.93<br>(11.83 − 14.06)	11.36<br>(10.31 − 12.61)	14.25<br>(12.91 − 15.65)	7.79<br>(5.94 − 9.92)	5.83<br>(4.01 − 7.96)	9.41<br>(6.74 − 12.39)	211.45<br>(166.54 − 264.51)	164.44<br>(117.31 − 216.35)	255.34<br>(188.15 − 326.84)
Northern Mariana Islands	12.95<br>(11.65 − 14.31)	13.43<br>(11.87 − 15.12)	12.26<br>(10.98 − 13.66)	7.11<br>(5.75 − 8.91)	5.73<br>(4.37 − 7.92)	8.15<br>(6.31 − 10.35)	216.18<br>(175.42 − 265.97)	214.39<br>(166.09 − 285.58)	214.78<br>(172.58 − 272.85)
Norway	6.91<br>(6.02 − 7.96)	6.38<br>(5.54 − 7.35)	7.46<br>(6.51 − 8.63)	2.20<br>(1.97 − 2.35)	2.21<br>(2.03 − 2.35)	2.15<br>(1.90 − 2.34)	63.46<br>(58.36 − 68.61)	63.67<br>(58.62 − 68.60)	62.81<br>(57.33 − 68.72)
Oman	11.42<br>(10.19 − 12.71)	10.43<br>(9.24 − 11.65)	12.75<br>(11.23 − 14.40)	1.57<br>(1.06 − 2.09)	1.72<br>(1.04 − 2.62)	1.50<br>(0.96 − 2.14)	52.11<br>(39.30 − 65.13)	50.38<br>(35.33 − 70.09)	55.97<br>(41.96 − 70.71)
Pakistan	10.37<br>(9.02 − 11.96)	10.28<br>(8.91 − 11.87)	10.50<br>(9.16 − 12.25)	5.34<br>(3.48 − 8.29)	5.20<br>(2.54 − 9.96)	5.49<br>(3.27 − 8.78)	176.88<br>(123.25 − 257.80)	174.86<br>(100.45 − 312.80)	179.62<br>(112.51 − 271.14)

Palau	14.52<br>(12.97 − 16.19)	13.46<br>(11.88 − 15.21)	15.41<br>(13.82 − 17.29)	6.59<br>(5.04 − 8.28)	5.88<br>(4.05 − 7.80)	7.28<br>(5.59 − 9.37)	245.55<br>(193.08 − 301.42)	259.94<br>(185.76 − 336.96)	216.79<br>(172.66 − 269.71)
Palestine	5.33<br>(4.62 − 6.03)	5.38<br>(4.65 − 6.12)	5.47<br>(4.74 − 6.23)	0.97<br>(0.74 − 1.17)	1.02<br>(0.69 − 1.32)	0.96<br>(0.71 − 1.23)	34.44<br>(28.83 − 40.46)	33.23<br>(25.70 − 41.96)	35.86<br>(29.47 − 43.77)
Panama	11.81<br>(10.47 − 13.30)	10.38<br>(9.09 − 11.82)	13.22<br>(11.50 – 15.00)	4.97<br>(3.90 − 5.97)	4.99<br>(3.82 − 6.11)	4.97<br>(3.99 − 5.92)	153.87<br>(123.67 − 182.57)	149.58<br>(116.60 − 180.71)	158.15<br>(130.21 − 186.72)
Papua New Guinea	10.99<br>(9.77 − 12.28)	11.01<br>(9.73 − 12.61)	11.06<br>(9.73 − 12.42)	9.06<br>(5.89 − 13.58)	7.46<br>(3.89 − 14.08)	10.89<br>(6.62 − 16.44)	290.54<br>(204.77 − 407.57)	269.57<br>(159.62 − 462.09)	316.46<br>(207.08 − 449.13)
Paraguay	10.88<br>(9.68 − 12.15)	9.40<br>(8.31 − 10.68)	12.30<br>(10.90 − 13.91)	5.47<br>(4.10 − 7.14)	4.40<br>(2.92 − 6.24)	6.48<br>(4.59 − 8.69)	176.82<br>(136.33 − 228.17)	143.20<br>(100.04 − 200.24)	209.44<br>(155.35 − 278.30)
Peru	10.45<br>(9.31 − 11.74)	9.65<br>(8.51 − 10.85)	11.20<br>(9.87 − 12.78)	4.25<br>(3.04 − 5.70)	3.71<br>(2.40 − 5.11)	4.73<br>(3.22 − 6.45)	153.50<br>(113.79 − 197.66)	141.94<br>(97.64 − 190.24)	163.92<br>(118.87 − 216.12)
Philippines	9.60<br>(8.37 − 11.03)	9.34<br>(8.11 − 10.69)	9.85<br>(8.56 − 11.42)	4.29<br>(3.54 − 5.18)	4.06<br>(2.99 − 5.70)	4.41<br>(3.38 − 5.56)	151.94<br>(125.29 − 178.28)	150.71<br>(109.92 − 191.68)	150.72<br>(119.16 − 183.66)
Poland	6.21<br>(5.40 − 7.09)	6.54<br>(5.65 − 7.48)	5.99<br>(5.17 − 6.86)	2.60<br>(2.37 − 2.84)	2.91<br>(2.59 − 3.22)	2.29<br>(2.01 − 2.56)	91.34<br>(83.49 − 99.86)	102.68<br>(92.05 − 113.39)	79.85<br>(71.17 − 89.29)
Portugal	5.65<br>(4.84 − 6.43)	5.76<br>(5.01 − 6.59)	5.55<br>(4.56 − 6.39)	3.10<br>(2.80 − 3.35)	3.18<br>(2.92 − 3.43)	3.04<br>(2.65 − 3.33)	85.82<br>(79.96 − 92.53)	85.71<br>(79.21 − 92.15)	86.17<br>(78.77 − 93.45)
Puerto Rico	7.83<br>(6.81 − 8.85)	6.42<br>(5.57 − 7.34)	9.12<br>(7.83 − 10.38)	1.97<br>(1.62 − 2.31)	1.90<br>(1.56 − 2.27)	2.01<br>(1.66 − 2.39)	66.93<br>(56.65 − 77.65)	64.28<br>(53.92 − 76.43)	68.77<br>(58.38 − 80.65)
Qatar	7.80<br>(6.85 − 8.70)	6.62<br>(5.70 − 7.46)	9.91<br>(8.78 − 11.15)	3.22<br>(2.22 − 4.42)	3.91<br>(2.42 − 5.82)	2.47<br>(1.69 − 3.54)	82.29<br>(61.54 − 108.28)	91.40<br>(63.89 − 128.50)	69.42<br>(53.14 − 89.01)

Republic of Korea	10.63<br>(9.32 − 12.29)	8.74<br>(7.48 − 10.14)	12.18<br>(10.48 − 14.26)	3.42<br>(2.79 − 4.02)	2.88<br>(2.08 − 3.53)	3.65<br>(2.83 − 4.43)	107.27<br>(93.63 − 123.56)	100.20<br>(79.99 − 121.38)	110.17<br>(93.61 − 130.11)
Republic of Moldova	5.67<br>(4.91 − 6.39)	6.49<br>(5.64 − 7.39)	4.98<br>(4.21 − 5.69)	2.08<br>(1.86 − 2.34)	2.84<br>(2.46 − 3.28)	1.52<br>(1.34 − 1.73)	66.94<br>(59.78 − 75.43)	88.46<br>(76.54 − 101.66)	49.08<br>(43.66 − 55.41)
Romania	9.66<br>(8.82 − 10.44)	10.44<br>(9.50 − 11.45)	9.00<br>(8.19 − 9.91)	5.25<br>(4.54 − 5.93)	5.79<br>(4.89 − 6.72)	4.77<br>(4.11 − 5.40)	150.40<br>(128.51 − 169.85)	166.77<br>(140.43 − 192.69)	135.16<br>(116.08 − 153.01)
Russian Federation	10.12<br>(8.88 − 11.54)	11.78<br>(10.30 − 13.47)	8.73<br>(7.66 − 9.98)	5.99<br>(5.51 − 6.44)	6.98<br>(6.26 − 7.67)	5.13<br>(4.57 − 5.64)	177.74<br>(164.71 − 191.72)	215.37<br>(193.94 − 236.16)	145.45<br>(130.03 − 160.39)
Rwanda	7.26<br>(6.28 − 8.56)	8.70<br>(7.45 − 10.30)	6.17<br>(5.24 − 7.17)	2.77<br>(1.01 − 7.37)	4.14<br>(1.04 − 14.03)	1.85<br>(0.81 − 4.75)	88.92<br>(39.00 − 209.75)	123.89<br>(39.73 − 388.75)	61.91<br>(33.87 − 137.79)
Saint Kitts and Nevis	9.97<br>(8.90 − 11.15)	9.38<br>(8.40 − 10.53)	10.69<br>(9.49 − 12.21)	4.36<br>(3.66 − 5.09)	4.47<br>(3.82 − 5.20)	4.32<br>(3.58 − 5.13)	132.11<br>(111.94 − 156.24)	134.60<br>(114.07 − 160.32)	129.44<br>(107.68 − 155.14)
Saint Lucia	9.61<br>(8.56 − 10.75)	8.07<br>(7.09 − 9.20)	11.12<br>(9.80 − 12.54)	3.69<br>(3.01 − 4.42)	3.20<br>(2.59 − 3.82)	4.15<br>(3.40 − 4.98)	118.66<br>(98.19 − 142.38)	102.08<br>(83.21 − 120.99)	134.33<br>(111.55 − 160.99)
Saint Vincent and the Grenadines	8.31<br>(7.43 − 9.22)	7.23<br>(6.40 − 8.17)	9.48<br>(8.41 − 10.65)	3.22<br>(2.83 − 3.65)	3.07<br>(2.66 − 3.49)	3.43<br>(2.95 − 3.94)	109.86<br>(96.29 − 124.86)	97.78<br>(85.21 − 111.04)	122.77<br>(106.22 − 141.75)
Samoa	15.40<br>(13.78 − 17.40)	13.18<br>(11.62 − 15.02)	17.47<br>(15.54 − 19.86)	8.10<br>(5.75 − 11.36)	6.55<br>(3.95 − 10.87)	9.29<br>(6.42 − 12.99)	272.27<br>(197.64 − 370.52)	258.39<br>(161.82 − 407.22)	281.17<br>(204.30 − 383.12)
San Marino	5.62<br>(4.80 − 6.60)	5.33<br>(4.49 − 6.21)	5.87<br>(5.00 − 6.90)	0.69<br>(0.42 − 1.02)	0.64<br>(0.36 – 1.00)	0.74<br>(0.44 − 1.13)	29.91<br>(21.78 − 40.14)	28.00<br>(19.82 − 39.04)	31.65<br>(22.99 − 42.61)

Sao Tome and Principe	5.96 (5.10 − 7.02)	6.43 (5.50 − 7.65)	5.51 (4.69 − 6.48)	2.45 (1.12 – 6.00)	2.32 (0.54 − 9.54)	2.54 (1.50 − 5.80)	90.59 (45.45 − 211.51)	92.49 (29.53 − 336.27)	87.90 (55.65 − 181.20)
Saudi Arabia	4.57 (3.96 − 5.17)	4.15 (3.55 − 4.80)	5.18 (4.47 − 5.88)	1.32 (0.90 − 1.75)	1.10 (0.59 − 1.57)	1.67 (1.08 − 2.57)	46.41 (33.48 − 59.46)	37.78 (22.02 − 52.08)	59.79 (42.13 − 83.88)
Senegal	5.13 (4.38 − 6.04)	5.83 (4.98 − 6.89)	4.50 (3.77 − 5.32)	2.75 (1.27 − 6.29)	2.98 (0.78 − 10.41)	2.53 (1.51 − 5.93)	94.31 (48.54 − 200.08)	106.38 (37.33 − 328.27)	82.87 (52.87 − 172.20)
Serbia	9.88 (9.04 − 10.69)	8.85 (7.98 − 9.73)	10.62 (9.69 − 11.63)	4.88 (3.87 − 6.08)	4.08 (2.90 − 5.48)	5.56 (4.28 − 7.28)	142.86 (115.43 − 173.60)	119.38 (89.69 − 156.86)	164.37 (130.83 − 208.97)
Seychelles	7.19 (6.29 − 8.12)	6.92 (6.05 − 7.89)	7.48 (6.50 − 8.62)	1.98 (1.31 − 2.92)	1.75 (0.61 − 3.69)	2.07 (1.15 − 3.26)	73.17 (54.77 − 94.85)	68.53 (33.75 − 119.03)	75.84 (51.98 − 105.55)
Sierra Leone	5.61 (4.80 − 6.56)	6.33 (5.47 − 7.44)	4.89 (4.10 − 5.78)	2.93 (1.21 − 8.14)	2.94 (0.63 − 12.81)	2.90 (1.52 − 7.45)	106.18 (52.39 − 262.54)	111.73 (36.45 − 404.08)	100.46 (57.27 − 223.35)
Singapore	8.72 (7.40 − 10.29)	7.66 (6.40 − 9.14)	9.80 (8.19 − 11.80)	1.32 (1.19 − 1.44)	1.06 (0.96 − 1.16)	1.54 (1.34 − 1.72)	57.01 (49.88 − 63.87)	49.42 (43.22 − 55.59)	64.02 (55.44 − 72.89)
Slovakia	5.59 (4.86 − 6.29)	5.68 (4.91 − 6.34)	5.60 (4.84 − 6.36)	2.15 (1.68 − 2.64)	2.08 (1.46 − 2.72)	2.18 (1.60 − 2.83)	73.18 (60.56 − 87.70)	70.33 (53.10 − 89.05)	75.45 (60.33 − 95.18)
Slovenia	5.27 (4.53 − 5.98)	5.03 (4.32 − 5.72)	5.52 (4.75 − 6.32)	1.37 (1.15 − 1.57)	1.37 (1.16 − 1.58)	1.35 (1.13 − 1.57)	43.32 (37.01 − 49.25)	42.52 (35.72 − 48.93)	43.81 (36.80 − 51.20)
Solomon Islands	24.22 (21.64 − 27.07)	20.17 (17.96 − 23.17)	28.30 (25.04 − 31.77)	11.68 (7.44 − 16.89)	10.56 (5.69 − 18.06)	12.79 (7.61 − 20.74)	359.24 (244.30 − 501.87)	372.44 (222.97 − 580.18)	345.78 (225.52 − 515.22)
Somalia	9.04 (7.74 − 10.81)	11.26 (9.59 − 13.31)	7.28 (6.08 − 8.70)	4.62 (0.79 − 16.89)	7.07 (0.49 − 32.98)	2.92 (0.77 − 13.89)	146.23 (36.19 − 474.18)	212.88 (29.99 − 880.64)	93.52 (34.77 − 357.19)

South Africa	4.63	4.72	4.59	1.46	1.50	1.42	49.67	50.78	48.39
	(4.02 − 5.39)	(4.08 − 5.43)	(3.93 − 5.41)	(1.20 − 1.68)	(0.97 − 1.93)	(1.12 − 1.68)	(42.12 − 56.90)	(36.57 − 6.82)	(39.75 − 56.87)
South Sudan	6.82	8.14	5.48	3.14	4.05	2.17	101.47	128.99	73.22
	(5.88 − 8.02)	(6.98 − 9.52)	(4.66 − 6.49)	(1.20 − 7.70)	(1.08 − 12.58)	(1.08 − 5.18)	(47.05 − 219.37)	(45.19 − 350.22)	(43.57 − 154.30)
Spain	6.05	5.74	6.38	2.27	2.56	2.09	63.46	65.52	62.36
	(5.27 − 6.89)	(4.98 − 6.51)	(5.48 − 7.37)	(2.02 − 2.47)	(2.33 − 2.77)	(1.80 − 2.32)	(58.71 − 68.10)	(60.26 − 70.58)	(56.69 − 68.22)
Sri Lanka	10.69	10.85	10.67	2.01	2.11	1.88	69.07	71.68	66.02
	(9.44 – 12.00)	(9.60 − 12.20)	(9.27 − 12.07)	(1.34 − 2.77)	(1.26 − 3.06)	(1.21 − 2.76)	(51.67 − 88.03)	(48.94 − 100.75)	(49.62 − 86.71)
Sudan	6.36	6.48	6.17	3.60	3.34	3.90	110.18	99.50	121.83
	(5.62 − 7.19)	(5.73 − 7.40)	(5.38 − 7.04)	(1.84 − 6.50)	(1.25 − 7.85)	(1.53 − 7.50)	(59.09 − 182.69)	(44.03 − 205.39)	(53.62 − 213.02)
Suriname	10.29	9.55	11.02	4.44	4.46	4.44	155.77	152.92	158.42
	(9.18 − 11.54)	(8.47 − 10.75)	(9.70 − 12.59)	(3.31 − 5.75)	(3.13 − 6.26)	(3.31 − 5.75)	(122.99 − 195.04)	(112.86 − 211.75)	(122.95 − 198.42)
Sweden	6.48	6.34	6.60	1.81	1.57	2.01	52.92	47.93	57.50
	(5.59 − 7.44)	(5.45 − 7.26)	(5.67 − 7.65)	(1.58 − 2.04)	(1.33 − 1.80)	(1.70 − 2.31)	(47.30 − 58.72)	(41.15 − 55.15)	(49.92 − 64.87)
Switzerland	5.52	5.00	6.03	1.66	1.67	1.67	46.55	45.65	47.41
	(4.73 − 6.42)	(4.20 − 5.81)	(5.18 − 7.03)	(1.43 − 1.84)	(1.51 − 1.83)	(1.40 − 1.88)	(42.15 − 51.03)	(41.94 − 49.83)	(41.95 − 52.61)
Syrian Arab	6.73	6.88	6.57	2.35	2.41	2.30	75.11	77.21	73.57
Republic	(5.96 − 7.48)	(6.14 − 7.68)	(5.78 − 7.38)	(1.56 − 3.31)	(1.47 − 3.86)	(1.50 − 3.17)	(54.37 − 101.19)	(51.37 − 114.62)	(53.14 − 99.06)
Taiwan	8.94	8.65	9.22	2.30	2.49	2.12	80.11	84.28	75.77
(Province of China)	(7.91 − 10.04)	(7.64 − 9.65)	(8.04 − 10.62)	(2.08 − 2.49)	(2.27 − 2.70)	(1.84 − 2.33)	(72.28 − 87.41)	(76.47 − 92.54)	(66.36 − 85.05)
Tajikistan	11.16	12.67	9.76	3.87	3.89	3.85	112.59	114.84	110.33
	(10.03 − 12.44)	(11.22 − 14.34)	(8.65 − 10.99)	(2.75 − 5.14)	(2.27 − 5.80)	(2.64 − 5.31)	(83.54 − 147.39)	(69.73 − 172.18)	(79.62 − 150.05)

Thailand	12.63 (11.30 − 14.29)	14.06 (12.49 − 15.83)	11.35 (10.03 – 13.00)	8.18 (6.29 − 10.53)	9.65 (6.92 − 12.99)	6.77 (4.90 − 8.92)	237.39 (186.44 − 308.38)	301.38 (225.96 − 407.24)	177.34 (137.47 − 224.58)
Timor-Leste	9.39 (8.25 − 10.71)	9.20 (8.02 − 10.57)	9.59 (8.40 − 10.91)	6.35 (3.51 − 11.85)	5.89 (2.79 − 14.32)	6.78 (3.71 − 12.44)	193.12 (121.46 − 327.81)	190.30 (106.41 − 401.69)	195.32 (115.09 − 318.46)
Togo	5.45 (4.69 − 6.37)	6.46 (5.56 − 7.55)	4.65 (3.95 − 5.46)	3.15 (1.34 − 8.02)	3.66 (0.85 − 14.59)	2.69 (1.42 – 7.00)	107.37 (50.83 − 256.83)	129.25 (39.95 − 446.37)	87.77 (50.91 − 202.87)
Tokelau	13.55 (12.03 − 15.22)	11.32 (9.97 − 12.95)	15.65 (13.95 − 17.75)	7.97 (5.84 − 11.52)	6.52 (3.95 − 11.47)	9.27 (6.24 − 12.31)	320.05 (251 − 442.39)	282.92 (184.46 − 478.89)	356.03 (255.77 − 446.32)
Tonga	10.04 (8.90 − 11.32)	8.96 (7.86 − 10.17)	10.82 (9.55 − 12.30)	3.91 (2.68 − 5.69)	3.01 (1.89 − 5.39)	4.48 (3.04 − 6.71)	134.15 (97.09 − 189.50)	127.01 (84.68 − 209.83)	138.26 (101.32 − 189.46)
Trinidad and Tobago	9.79 (8.80 − 10.98)	8.95 (8.03 − 10.01)	10.65 (9.42 − 12.02)	4.95 (3.80 − 6.31)	5.12 (3.87 − 6.61)	4.78 (3.69 − 6.09)	169.90 (133.29 − 215.74)	177.15 (136.21 − 227.13)	162.27 (126.68 − 205.22)
Tunisia	6.05 (5.29 − 6.80)	5.96 (5.22 − 6.78)	6.19 (5.34 – 7.00)	2.14 (1.14 − 3.59)	2.13 (0.95 − 4.06)	2.16 (1.13 − 3.83)	60.86 (37.89 − 96.26)	57.71 (32.23 − 105.15)	64.00 (40.96 − 104.05)
Turkey	6.85 (6.05 − 7.63)	6.75 (5.93 − 7.59)	6.99 (6.13 − 7.92)	3.84 (2.98 − 4.78)	4.35 (3.13 − 5.87)	3.40 (2.54 − 4.49)	107.74 (86.97 − 132.16)	119.01 (89.38 − 156.37)	97.22 (76.69 − 122.74)
Turkmenistan	12.15 (10.92 − 13.50)	13.98 (12.44 − 15.76)	10.36 (9.30 − 11.63)	8.38 (6.32 − 11.99)	11.05 (7.64 − 18.31)	6.22 (3.82 − 8.58)	264.08 (202.57 − 363.86)	343.56 (240.38 − 555.95)	191.21 (121.22 − 261.37)
Tuvalu	16.93 (15.06 – 19.00)	15.47 (13.57 − 17.47)	17.80 (15.88 − 20.43)	10.45 (6.82 − 15.16)	9.37 (5.26 − 16.67)	10.87 (6.55 − 15.90)	358.85 (245.49 − 500.52)	371.43 (225.47 − 607.89)	333.14 (215.44 − 474.79)
Uganda	6.79 (5.83 − 8.06)	8.34 (7.19 − 9.78)	5.56 (4.70 − 6.59)	2.33 (0.88 − 6.13)	3.61 (0.94 − 12.04)	1.39 (0.69 − 3.58)	78.61 (35.91 − 188.61)	114.97 (39.13 − 356.40)	48.72 (29.53 − 108.61)

Ukraine	9.20 (8.07 − 10.49)	11.05 (9.64 − 12.64)	7.53 (6.56 − 8.68)	4.97 (3.78 − 6.28)	6.22 (4.30 − 8.38)	4.01 (2.72 − 5.64)	142.97 (107.72 − 179.64)	185.21 (127.74 − 249.89)	107.64 (74.14 − 148.03)
United Arab Emirates	5.73 (4.99 − 6.43)	5.30 (4.60 − 6.02)	6.95 (6.04 − 7.82)	3.69 (2.72 − 4.85)	2.25 (1.52 − 3.10)	9.77 (6.16 − 13.74)	86.38 (67.94 − 109.03)	59.27 (44.77 − 78.14)	191.68 (135.01 − 259.80)
United Kingdom	7.08 (6.24 − 8.10)	6.06 (5.31 − 6.91)	8.03 (7.05 − 9.21)	3.01 (2.76 − 3.14)	2.72 (2.56 − 2.81)	3.26 (2.94 − 3.44)	85.03 (80.48 − 89.34)	76.80 (73.13 − 80.13)	92.62 (87.19 − 98.04)
United Republic of Tanzania	6.92 (5.94 − 8.15)	8.02 (6.89 − 9.58)	5.95 (5.08 − 6.99)	2.24 (0.87 − 6.18)	2.83 (0.63 − 11.17)	1.73 (0.83 – 4.00)	74.75 (35.77 − 182.11)	92.93 (31.09 − 320.61)	58.42 (33.93 − 119.78)
United States of America	6.30 (5.49 − 7.24)	5.59 (4.88 − 6.39)	7.04 (6.12 − 8.17)	3.24 (2.96 − 3.42)	3.06 (2.85 − 3.23)	3.40 (3.07 − 3.61)	100.36 (94.75 − 105.76)	91.75 (87.03 − 96.63)	108.18 (101.61 − 115.02)
United States Virgin Islands	9.84 (8.78 − 11.05)	8.85 (7.84 − 9.94)	10.80 (9.46 − 12.37)	2.60 (1.96 − 3.39)	3.08 (2.19 − 4.20)	2.24 (1.59 − 3.19)	92.52 (69.94 − 120.09)	105.02 (73.50 − 140.13)	81.22 (59.40 − 112.73)
Uruguay	13.40 (12.00 – 15.00)	10.59 (9.45 − 12.02)	15.87 (13.89 − 17.96)	6.68 (6.18 − 7.15)	5.97 (5.42 − 6.47)	7.21 (6.55 − 7.79)	210.03 (196.11 − 224.86)	189.49 (172.98 − 205.32)	226.72 (210.50 − 242.67)
Uzbekistan	6.87 (6.11 − 7.68)	7.75 (6.80 − 8.70)	6.02 (5.23 − 6.79)	4.51 (3.77 − 5.42)	5.96 (4.90 − 7.34)	3.36 (2.80 − 4.01)	121.28 (103.75 − 143.62)	159.12 (134.29 − 193.57)	88.70 (75.49 − 104.28)
Vanuatu	17.11 (15.29 − 19.23)	16.38 (14.39 − 18.61)	17.72 (15.77 − 20.06)	9.71 (5.90 − 14.23)	9.67 (5.42 − 17.79)	9.59 (4.18 − 14.90)	346.50 (228.12 − 497.84)	389.77 (241.97 − 644.17)	302.20 (158.32 − 453.35)
Venezuela (Bolivarian Republic of Venezuela)	12.35 (11.02 − 14.08)	9.98 (8.75 − 11.37)	14.50 (12.77 − 16.71)	5.51 (4.11 − 7.12)	4.84 (3.56 − 6.41)	6.10 (4.43 − 7.96)	184.51 (138.38 − 235.54)	160.38 (120.46 − 210.85)	206.21 (152.37 − 266.90)

Viet Nam	10.49<br>(9.29 − 11.92)	11.65<br>(10.20 − 13.24)	9.58<br>(8.43 − 10.98)	5.23<br>(2.92 − 9.86)	6.08<br>(2.92 − 12.27)	4.39<br>(2.53 − 8.97)	151.17<br>(100.92 − 239.12)	183.31<br>(103.64 − 320.19)	119.79<br>(84.93 − 201.46)
Yemen	7.67<br>(6.81 − 8.73)	7.70<br>(6.82 − 8.77)	7.68<br>(6.71 − 8.78)	5.53<br>(2.54 − 10.61)	6.12<br>(2.06 − 14.50)	5.02<br>(2.16 − 11.01)	150.97<br>(78.71 − 267.26)	157.83<br>(62.83 − 332.98)	144.87<br>(67.46 − 287.79)
Zambia	7.70<br>(6.65 − 8.97)	9.43<br>(8.19 − 11.01)	6.12<br>(5.21 − 7.25)	3.63<br>(1.65 − 7.67)	4.59<br>(1.55 − 12.96)	2.80<br>(1.47 − 6.44)	113.69<br>(56.80 − 225.18)	140.65<br>(55.80 − 364.63)	88.16<br>(52.38 − 192.61)
Zimbabwe	8.46<br>(7.44 − 9.72)	9.59<br>(8.42 − 10.93)	7.54<br>(6.55 − 8.81)	6.01<br>(4.00 − 9.61)	6.99<br>(4.25 − 12.35)	5.24<br>(3.17 − 8.54)	184.88<br>(127.85 − 282.71)	221.30<br>(141.41 − 380.50)	154.61<br>(98.78 − 241.55)

ASIR age-standardized incidence rate, *ASMR* age-standardized mortality rate, *ASDR* age-standardized disability-adjusted life-year rate, *UI* uncertainty interval

Table S8 EAPC of ASIR, ASMR, and ASDR for subarachnoid hemorrhage (SAH) in 204 countries and territories from 1990 to 2021

Location	EAPC of ASIR (95% CI)			EAPC of ASMR (95% CI)			EAPC of ASDR (95% CI)		
	Both	Male	Female	Both	Male	Female	Both	Male	Female
Afghanistan	-0.96 (-1.14 to -0.79)	-0.85 (-1.01 to -0.70)	-0.93 (-1.11 to -0.75)	-1.60 (-1.85 to -1.36)	-1.86 (-2.16 to -1.57)	-0.92 (-1.09 to -0.75)	-1.59 (-1.80 to -1.37)	-1.80 (-2.07 to -1.53)	-1.14 (-1.29 to -0.98)
Albania	-0.24 (-0.31 to -0.17)	-0.39 (-0.46 to -0.31)	-0.19 (-0.27 to -0.12)	-1.27 (-1.51 to -1.02)	-2.28 (-2.48 to -2.09)	-0.63 (-0.92 to -0.34)	-1.54 (-1.74 to -1.33)	-2.11 (-2.30 to -1.92)	-1.09 (-1.32 to -0.86)
Algeria	-1.65 (-1.76 to -1.54)	-1.65 (-1.77 to -1.53)	-1.62 (-1.74 to -1.50)	-1.65 (-1.73 to -1.56)	-2.19 (-2.23 to -2.16)	-0.95 (-1.13 to -0.77)	-2.11 (-2.14 to -2.07)	-2.45 (-2.50 to -2.40)	-1.75 (-1.82 to -1.68)
American Samoa	-0.38 (-0.41 to -0.35)	-0.64 (-0.69 to -0.59)	-0.14 (-0.16 to -0.11)	-0.52 (-0.63 to -0.41)	-0.67 (-0.74 to -0.61)	-0.34 (-0.49 to -0.18)	-0.44 (-0.52 to -0.36)	-0.64 (-0.71 to -0.57)	-0.20 (-0.35 to -0.05)
Andorra	-0.52 (-0.58 to -0.46)	-0.62 (-0.68 to -0.56)	-0.49 (-0.56 to -0.42)	-1.71 (-1.88 to -1.53)	-2.05 (-2.27 to -1.82)	-1.64 (-1.80 to -1.48)	-1.99 (-2.13 to -1.84)	-2.15 (-2.29 to -2.00)	-1.98 (-2.11 to -1.84)
Angola	-0.84 (-0.91 to -0.77)	-0.82 (-0.89 to -0.75)	-0.80 (-0.88 to -0.72)	-1.36 (-1.47 to -1.26)	-1.42 (-1.52 to -1.33)	-1.14 (-1.27 to -1.01)	-1.34 (-1.43 to -1.25)	-1.34 (-1.42 to -1.26)	-1.22 (-1.34 to -1.10)
Antigua and Barbuda	-0.74 (-0.81 to -0.67)	-0.77 (-0.84 to -0.71)	-0.69 (-0.77 to -0.61)	-1.61 (-1.84 to -1.38)	-1.81 (-2.13 to -1.50)	-1.34 (-1.57 to -1.12)	-1.75 (-1.94 to -1.56)	-2.09 (-2.33 to -1.86)	-1.39 (-1.59 to -1.20)
Argentina	-1.85 (-2.00 to -1.71)	-1.73 (-1.89 to -1.58)	-1.92 (-2.06 to -1.78)	-3.20 (-3.55 to -2.85)	-3.32 (-3.76 to -2.89)	-3.05 (-3.35 to -2.75)	-3.29 (-3.57 to -3.01)	-3.50 (-3.84 to -3.16)	-3.09 (-3.32 to -2.85)
Armenia	-1.30 (-1.44 to -1.17)	-1.00 (-1.11 to -0.89)	-1.60 (-1.75 to -1.44)	-2.03 (-2.53 to -1.53)	-1.62 (-2.10 to -1.13)	-2.49 (-3.01 to -1.98)	-1.86 (-2.28 to -1.44)	-1.40 (-1.85 to -0.95)	-2.42 (-2.82 to -2.02)

Australia	-0.63 (-0.71 to -0.55)	-0.46 (-0.54 to -0.37)	-0.73 (-0.80 to -0.65)	-1.86 (-1.97 to -1.75)	-1.37 (-1.55 to -1.19)	-2.15 (-2.25 to -2.05)	-2.05 (-2.14 to -1.97)	-1.85 (-2.00 to -1.71)	-2.17 (-2.25 to -2.09)
Austria	-0.76 (-0.98 to -0.54)	-0.82 (-1.02 to -0.63)	-0.69 (-0.93 to -0.45)	-1.36 (-1.50 to -1.22)	-0.93 (-1.07 to -0.80)	-1.56 (-1.73 to -1.39)	-1.72 (-1.86 to -1.58)	-1.55 (-1.68 to -1.42)	-1.80 (-1.95 to -1.64)
Azerbaijan	-0.65 (-0.77 to -0.53)	-0.86 (-0.99 to -0.73)	-0.49 (-0.60 to -0.38)	-1.39 (-1.72 to -1.05)	-2.08 (-2.46 to -1.70)	-0.87 (-1.16 to -0.57)	-1.67 (-1.95 to -1.40)	-2.13 (-2.46 to -1.81)	-1.25 (-1.48 to -1.03)
Bahamas	-0.37 (-0.42 to -0.31)	-0.22 (-0.26 to -0.18)	-0.48 (-0.54 to -0.41)	-0.90 (-1.03 to -0.76)	-0.89 (-1.08 to -0.70)	-0.92 (-1.05 to -0.80)	-0.91 (-1.02 to -0.79)	-0.83 (-0.98 to -0.67)	-0.98 (-1.10 to -0.87)
Bahrain	-1.41 (-1.51 to -1.30)	-1.38 (-1.49 to -1.28)	-1.48 (-1.59 to -1.36)	-3.66 (-3.93 to -3.38)	-3.53 (-3.82 to -3.24)	-3.82 (-4.10 to -3.55)	-3.58 (-3.78 to -3.37)	-3.40 (-3.61 to -3.19)	-3.74 (-3.97 to -3.51)
Bangladesh	-0.49 (-0.57 to -0.42)	-0.36 (-0.43 to -0.29)	-0.50 (-0.59 to -0.42)	-1.74 (-2 to -1.47)	-1.96 (-2.27 to -1.65)	-1.14 (-1.33 to -0.96)	-1.81 (-1.97 to -1.65)	-1.94 (-2.14 to -1.75)	-1.30 (-1.41 to -1.19)
Barbados	-0.55 (-0.66 to -0.44)	-0.61 (-0.72 to -0.51)	-0.51 (-0.62 to -0.39)	-0.74 (-0.91 to -0.57)	-1.07 (-1.25 to -0.89)	-0.43 (-0.63 to -0.23)	-0.87 (-1.00 to -0.74)	-1.15 (-1.29 to -1.01)	-0.61 (-0.77 to -0.44)
Belarus	-0.46 (-0.52 to -0.39)	-0.26 (-0.31 to -0.20)	-0.63 (-0.70 to -0.56)	-0.59 (-0.80 to -0.39)	-0.04 (-0.15 to 0.07)	-1.17 (-1.45 to -0.88)	-0.68 (-0.89 to -0.48)	-0.17 (-0.32 to -0.02)	-1.29 (-1.55 to -1.02)
Belgium	-0.67 (-0.91 to -0.43)	-0.53 (-0.77 to -0.29)	-0.74 (-0.98 to -0.51)	-0.40 (-0.79 to -0.01)	0.27 (-0.16 to 0.70)	-0.78 (-1.17 to -0.39)	-1.05 (-1.36 to -0.73)	-0.75 (-1.06 to -0.43)	-1.21 (-1.54 to -0.88)
Belize	-0.60 (-0.66 to -0.53)	-0.49 (-0.55 to -0.42)	-0.73 (-0.80 to -0.65)	-1.38 (-1.60 to -1.15)	-1.67 (-1.92 to -1.42)	-1.13 (-1.40 to -0.86)	-1.48 (-1.66 to -1.30)	-1.67 (-1.88 to -1.47)	-1.33 (-1.55 to -1.10)
Benin	-0.62 (-0.68 to -0.57)	-0.62 (-0.68 to -0.56)	-0.60 (-0.65 to -0.55)	-0.92 (-1.02 to -0.83)	-1.00 (-1.10 to -0.90)	-0.90 (-1.00 to -0.80)	-0.86 (-0.95 to -0.76)	-0.90 (-1.01 to -0.80)	-0.85 (-0.95 to -0.75)

Bermuda	-1.07 (-1.16 to -0.99)	-0.81 (-0.87 to -0.76)	-1.26 (-1.37 to -1.15)	-2.46 (-2.64 to -2.28)	-1.81 (-2 to -1.63)	-3.43 (-3.71 to -3.16)	-2.38 (-2.55 to -2.22)	-1.93 (-2.12 to -1.74)	-3.00 (-3.22 to -2.78)
Bhutan	-1.32 (-1.41 to -1.22)	-1.26 (-1.34 to -1.17)	-1.43 (-1.54 to -1.32)	-1.91 (-1.97 to -1.85)	-1.92 (-1.96 to -1.89)	-1.97 (-2.08 to -1.85)	-1.99 (-2.05 to -1.94)	-1.90 (-1.93 to -1.87)	-2.13 (-2.24 to -2.01)
Bolivia (Plurinational State of Bolivia)	-1.27 (-1.36 to -1.19)	-1.27 (-1.36 to -1.19)	-1.27 (-1.35 to -1.18)	-1.61 (-1.67 to -1.55)	-1.91 (-1.97 to -1.85)	-1.44 (-1.52 to -1.36)	-1.99 (-2.08 to -1.91)	-2.19 (-2.29 to -2.09)	-1.87 (-1.95 to -1.78)
Bosnia and Herzegovina	-1.09 (-1.15 to -1.02)	-1.12 (-1.19 to -1.05)	-1.07 (-1.13 to -1.02)	-3.20 (-3.47 to -2.94)	-3.34 (-3.61 to -3.07)	-3.11 (-3.37 to -2.86)	-3.19 (-3.42 to -2.95)	-3.30 (-3.55 to -3.04)	-3.08 (-3.31 to -2.86)
Botswana	-0.60 (-0.68 to -0.52)	-0.78 (-0.92 to -0.64)	-0.40 (-0.42 to -0.37)	-2.11 (-2.31 to -1.91)	-2.73 (-3.09 to -2.37)	-1.33 (-1.83 to -0.83)	-1.85 (-2.02 to -1.68)	-2.47 (-2.79 to -2.14)	-1.04 (-1.49 to -0.60)
Brazil	-1.38 (-1.53 to -1.24)	-1.27 (-1.39 to -1.16)	-1.47 (-1.64 to -1.30)	-1.07 (-1.28 to -0.86)	-1.03 (-1.19 to -0.87)	-1.10 (-1.34 to -0.86)	-1.66 (-1.84 to -1.48)	-1.71 (-1.84 to -1.58)	-1.64 (-1.85 to -1.43)
Brunei Darussalam	-1.63 (-1.79 to -1.46)	-1.47 (-1.64 to -1.29)	-1.84 (-2 to -1.68)	-2.26 (-2.48 to -2.04)	-1.76 (-2.05 to -1.46)	-2.65 (-2.83 to -2.47)	-2.24 (-2.47 to -2.01)	-1.86 (-2.13 to -1.59)	-2.61 (-2.82 to -2.40)
Bulgaria	0.12 (0.08 to 0.15)	0 (-0.03 to 0.03)	0.26 (0.23 to 0.29)	0.58 (0.40 to 0.76)	0.55 (0.35 to 0.76)	0.64 (0.43 to 0.84)	-0.10 (-0.25 to 0.05)	-0.13 (-0.30 to 0.03)	-0.05 (-0.25 to 0.15)
Burkina Faso	-0.44 (-0.48 to -0.39)	-0.40 (-0.45 to -0.35)	-0.48 (-0.52 to -0.43)	-0.28 (-0.36 to -0.21)	-0.45 (-0.54 to -0.36)	0.02 (-0.11 to 0.15)	-0.33 (-0.40 to -0.26)	-0.38 (-0.48 to -0.29)	-0.26 (-0.33 to -0.20)
Burundi	-0.81 (-0.89 to -0.73)	-0.66 (-0.72 to -0.60)	-1.12 (-1.23 to -1.01)	-2.16 (-2.39 to -1.92)	-2.18 (-2.44 to -1.93)	-2.47 (-2.68 to -2.26)	-2.18 (-2.41 to -1.96)	-2.23 (-2.49 to -1.97)	-2.43 (-2.62 to -2.24)

Côte d'Ivoire	-0.57<br>(-0.63 to -0.52)	-0.67<br>(-0.75 to -0.59)	-0.43<br>(-0.47 to -0.39)	-0.56<br>(-0.74 to -0.37)	-0.99<br>(-1.24 to -0.74)	0.16<br>(-0.04 to 0.37)	-0.54<br>(-0.72 to -0.36)	-0.91<br>(-1.15 to -0.68)	0.14<br>(-0.05 to 0.32)
Cabo Verde	-0.57<br>(-0.64 to -0.51)	-0.69<br>(-0.75 to -0.62)	-0.48<br>(-0.54 to -0.42)	-1.04<br>(-1.17 to -0.90)	-1.42<br>(-1.61 to -1.23)	-0.93<br>(-1.07 to -0.79)	-1.19<br>(-1.25 to -1.13)	-1.46<br>(-1.56 to -1.37)	-1.20<br>(-1.26 to -1.14)
Cambodia	-0.79<br>(-0.84 to -0.73)	-0.88<br>(-0.94 to -0.82)	-0.72<br>(-0.78 to -0.66)	-1.83<br>(-1.94 to -1.71)	-1.87<br>(-1.94 to -1.79)	-1.86<br>(-2.00 to -1.71)	-1.98<br>(-2.08 to -1.87)	-1.93<br>(-2.01 to -1.86)	-2.05<br>(-2.18 to -1.92)
Cameroon	-0.34<br>(-0.41 to -0.27)	-0.22<br>(-0.30 to -0.13)	-0.48<br>(-0.54 to -0.42)	-0.52<br>(-0.76 to -0.28)	-0.64<br>(-0.90 to -0.38)	-0.37<br>(-0.58 to -0.16)	-0.38<br>(-0.65 to -0.12)	-0.46<br>(-0.74 to -0.18)	-0.29<br>(-0.52 to -0.06)
Canada	-1.02<br>(-1.08 to -0.96)	-0.67<br>(-0.70 to -0.63)	-1.21<br>(-1.28 to -1.14)	-1.76<br>(-1.89 to -1.63)	-1.18<br>(-1.35 to -1.01)	-2.12<br>(-2.26 to -1.97)	-1.99<br>(-2.12 to -1.87)	-1.58<br>(-1.70 to -1.45)	-2.27<br>(-2.42 to -2.12)
Central African Republic	-0.15<br>(-0.16 to -0.13)	-0.07<br>(-0.08 to -0.05)	-0.22<br>(-0.24 to -0.19)	-0.42<br>(-0.46 to -0.38)	-0.36<br>(-0.41 to -0.31)	-0.48<br>(-0.55 to -0.40)	-0.48<br>(-0.53 to -0.44)	-0.41<br>(-0.46 to -0.37)	-0.60<br>(-0.67 to -0.52)
Chad	-0.32<br>(-0.38 to -0.25)	-0.36<br>(-0.43 to -0.29)	-0.34<br>(-0.39 to -0.29)	-0.01<br>(-0.21 to 0.19)	-0.27<br>(-0.53 to -0.01)	0.24<br>(0.12 to 0.36)	-0.01<br>(-0.21 to 0.19)	-0.20<br>(-0.45 to 0.06)	0.18<br>(0.06 to 0.30)
Chile	-0.37<br>(-0.49 to -0.26)	-0.24<br>(-0.31 to -0.17)	-0.44<br>(-0.58 to -0.31)	-0.64<br>(-0.98 to -0.30)	-0.21<br>(-0.55 to 0.14)	-0.94<br>(-1.28 to -0.60)	-1.10<br>(-1.37 to -0.82)	-0.81<br>(-1.10 to -0.51)	-1.31<br>(-1.57 to -1.04)
China	-3.70<br>(-4.08 to -3.31)	-3.72<br>(-4.15 to -3.28)	-3.70<br>(-4.04 to -3.37)	-6.66<br>(-7.32 to -6.01)	-6.08<br>(-6.63 to -5.53)	-7.27<br>(-8.02 to -6.51)	-6.29<br>(-6.89 to -5.69)	-5.81<br>(-6.34 to -5.28)	-6.84<br>(-7.52 to -6.16)
Colombia	-0.42<br>(-0.50 to -0.34)	-0.33<br>(-0.41 to -0.24)	-0.53<br>(-0.61 to -0.46)	-0.21<br>(-0.49 to 0.08)	-0.04<br>(-0.33 to 0.26)	-0.39<br>(-0.67 to -0.10)	-0.67<br>(-0.87 to -0.48)	-0.46<br>(-0.67 to -0.25)	-0.88<br>(-1.07 to -0.68)
Comoros	-0.81<br>(-0.88 to -0.74)	-0.76<br>(-0.83 to -0.69)	-0.87<br>(-0.94 to -0.80)	-1.71<br>(-1.97 to -1.46)	-1.73<br>(-2 to -1.46)	-1.67<br>(-1.89 to -1.45)	-1.70<br>(-1.97 to -1.43)	-1.72<br>(-2.02 to -1.42)	-1.66<br>(-1.90 to -1.43)

Congo	-0.90 (-0.98 to -0.82)	-1.12 (-1.21 to -1.02)	-0.68 (-0.75 to -0.62)	-1.45 (-1.59 to -1.32)	-1.98 (-2.14 to -1.82)	-0.96 (-1.14 to -0.79)	-1.43 (-1.58 to -1.29)	-1.95 (-2.11 to -1.78)	-0.92 (-1.10 to -0.74)
Cook Islands	-1.37 (-1.50 to -1.23)	-1.08 (-1.20 to -0.95)	-1.64 (-1.78 to -1.49)	-3.49 (-3.72 to -3.27)	-3.15 (-3.35 to -2.95)	-3.77 (-4.02 to -3.52)	-3.05 (-3.25 to -2.85)	-2.66 (-2.85 to -2.47)	-3.42 (-3.64 to -3.20)
Costa Rica	-0.68 (-0.75 to -0.61)	-0.32 (-0.37 to -0.27)	-0.96 (-1.04 to -0.88)	-0.22 (-0.40 to -0.05)	0.31 (0.16 to 0.47)	-0.68 (-0.90 to -0.46)	-0.37 (-0.53 to -0.22)	0.18 (0.04 to 0.32)	-0.84 (-1.04 to -0.64)
Croatia	-1.51 (-1.63 to -1.39)	-1.45 (-1.58 to -1.32)	-1.57 (-1.68 to -1.46)	-1.94 (-2.15 to -1.72)	-1.73 (-1.88 to -1.58)	-2.14 (-2.42 to -1.86)	-2.39 (-2.61 to -2.17)	-2.14 (-2.30 to -1.99)	-2.60 (-2.88 to -2.31)
Cuba	-1.03 (-1.11 to -0.95)	-0.78 (-0.84 to -0.71)	-1.21 (-1.30 to -1.11)	-1.66 (-1.91 to -1.40)	-1.38 (-1.66 to -1.11)	-1.89 (-2.14 to -1.65)	-1.84 (-2.05 to -1.62)	-1.58 (-1.82 to -1.35)	-2.05 (-2.27 to -1.84)
Cyprus	-1.87 (-2.06 to -1.68)	-1.69 (-1.84 to -1.53)	-2.04 (-2.28 to -1.80)	-4.04 (-4.27 to -3.81)	-3.76 (-3.95 to -3.57)	-4.32 (-4.57 to -4.06)	-3.82 (-3.98 to -3.66)	-3.47 (-3.59 to -3.35)	-4.11 (-4.30 to -3.93)
Czechia	-1.52 (-1.65 to -1.39)	-1.20 (-1.31 to -1.09)	-1.77 (-1.91 to -1.62)	-2.93 (-3.19 to -2.66)	-2.60 (-2.81 to -2.39)	-3.15 (-3.46 to -2.84)	-2.90 (-3.08 to -2.73)	-2.71 (-2.90 to -2.52)	-3.03 (-3.23 to -2.83)
Democratic People's Republic of Korea	-0.99 (-1.07 to -0.91)	-0.80 (-0.87 to -0.73)	-1.12 (-1.21 to -1.03)	-1.61 (-1.68 to -1.55)	-1.45 (-1.51 to -1.38)	-1.75 (-1.82 to -1.68)	-1.42 (-1.49 to -1.35)	-1.28 (-1.34 to -1.22)	-1.57 (-1.64 to -1.51)
Democratic Republic of the Congo	-0.02 (-0.05 to 0.01)	0.05 (0.01 to 0.08)	-0.06 (-0.10 to -0.01)	-0.12 (-0.22 to -0.03)	0.11 (-0.03 to 0.26)	-0.26 (-0.34 to -0.17)	-0.24 (-0.32 to -0.17)	-0.05 (-0.17 to 0.07)	-0.49 (-0.56 to -0.43)
Denmark	-1.32 (-1.39 to -1.25)	-0.87 (-0.93 to -0.80)	-1.63 (-1.71 to -1.54)	-2.06 (-2.29 to -1.83)	-1.51 (-1.78 to -1.24)	-2.43 (-2.65 to -2.20)	-2.65 (-2.86 to -2.44)	-2.23 (-2.46 to -1.99)	-2.95 (-3.15 to -2.74)

Djibouti	-0.51<br>(-0.57 to -0.46)	-0.52<br>(-0.57 to -0.47)	-0.58<br>(-0.64 to -0.52)	-1.13<br>(-1.27 to -0.99)	-1.32<br>(-1.48 to -1.16)	-1.00<br>(-1.09 to -0.90)	-1.05<br>(-1.21 to -0.90)	-1.16<br>(-1.33 to -0.99)	-0.98<br>(-1.09 to -0.87)
Dominica	-0.67<br>(-0.74 to -0.60)	-0.57<br>(-0.64 to -0.50)	-0.74<br>(-0.80 to -0.68)	-0.98<br>(-1.15 to -0.81)	-1.24<br>(-1.43 to -1.05)	-0.91<br>(-1.07 to -0.76)	-0.85<br>(-1.04 to -0.67)	-1.00<br>(-1.20 to -0.81)	-0.87<br>(-1.05 to -0.68)
Dominican Republic	0.42<br>(0.36 to 0.47)	0.30<br>(0.25 to 0.35)	0.52<br>(0.46 to 0.58)	-0.37<br>(-0.57 to -0.17)	-0.56<br>(-0.72 to -0.39)	-0.24<br>(-0.48 to 0)	-0.45<br>(-0.66 to -0.24)	-0.56<br>(-0.77 to -0.36)	-0.35<br>(-0.57 to -0.13)
Ecuador	-0.32<br>(-0.35 to -0.28)	-0.24<br>(-0.30 to -0.18)	-0.39<br>(-0.42 to -0.37)	1.00<br>(0.74 to 1.26)	1.09<br>(0.83 to 1.34)	0.90<br>(0.62 to 1.19)	0.21<br>(-0.06 to 0.48)	0.31<br>(0.02 to 0.60)	0.11<br>(-0.16 to 0.39)
Egypt	-1.20<br>(-1.27 to -1.13)	-1.29<br>(-1.37 to -1.21)	-1.09<br>(-1.15 to -1.02)	-2.92<br>(-3 to -2.84)	-3.57<br>(-3.67 to -3.47)	-1.73<br>(-1.92 to -1.55)	-3.34<br>(-3.44 to -3.24)	-3.75<br>(-3.84 to -3.66)	-2.70<br>(-2.87 to -2.52)
El Salvador	-1.34<br>(-1.55 to -1.13)	-1.61<br>(-1.86 to -1.35)	-1.23<br>(-1.41 to -1.05)	-1.47<br>(-1.89 to -1.05)	-1.84<br>(-2.29 to -1.39)	-1.27<br>(-1.67 to -0.88)	-1.68<br>(-2.11 to -1.25)	-1.94<br>(-2.45 to -1.44)	-1.54<br>(-1.91 to -1.17)
Equatorial Guinea	-1.65<br>(-1.81 to -1.49)	-1.89<br>(-2.07 to -1.71)	-1.48<br>(-1.61 to -1.35)	-4.15<br>(-4.71 to -3.58)	-5.18<br>(-5.93 to -4.42)	-3.10<br>(-3.47 to -2.74)	-3.79<br>(-4.28 to -3.29)	-4.70<br>(-5.37 to -4.02)	-2.86<br>(-3.18 to -2.54)
Eritrea	-0.54<br>(-0.61 to -0.48)	-0.67<br>(-0.73 to -0.61)	-0.46<br>(-0.53 to -0.40)	-1.40<br>(-1.55 to -1.25)	-1.64<br>(-1.76 to -1.53)	-0.98<br>(-1.17 to -0.79)	-1.39<br>(-1.53 to -1.25)	-1.66<br>(-1.77 to -1.54)	-0.97<br>(-1.16 to -0.78)
Estonia	-1.73<br>(-1.88 to -1.58)	-1.59<br>(-1.71 to -1.47)	-1.85<br>(-2.03 to -1.68)	-2.37<br>(-3.03 to -1.71)	-2.25<br>(-2.74 to -1.76)	-2.52<br>(-3.38 to -1.66)	-2.98<br>(-3.50 to -2.45)	-2.92<br>(-3.36 to -2.48)	-3.06<br>(-3.74 to -2.37)
Eswatini	-0.16<br>(-0.27 to -0.05)	0.01<br>(-0.06 to 0.08)	-0.31<br>(-0.45 to -0.17)	0.04<br>(-0.42 to 0.50)	-0.27<br>(-0.65 to 0.10)	0.34<br>(-0.21 to 0.88)	0.19<br>(-0.28 to 0.66)	0.04<br>(-0.38 to 0.45)	0.35<br>(-0.18 to 0.89)
Ethiopia	-1.95<br>(-2.11 to -1.79)	-1.74<br>(-1.88 to -1.60)	-2.29<br>(-2.48 to -2.10)	-2.96<br>(-3.11 to -2.81)	-3.00<br>(-3.12 to -2.87)	-2.88<br>(-3.05 to -2.71)	-2.87<br>(-3.00 to -2.74)	-2.89<br>(-3 to -2.78)	-2.76<br>(-2.92 to -2.60)

Fiji	-0.91<br>(-1 to -0.82)	-1.11<br>(-1.20 to -1.02)	-0.90<br>(-0.99 to -0.82)	-1.04<br>(-1.19 to -0.89)	-1.36<br>(-1.48 to -1.25)	-1.01<br>(-1.16 to -0.85)	-1.07<br>(-1.18 to -0.96)	-1.25<br>(-1.35 to -1.14)	-1.01<br>(-1.12 to -0.90)
Finland	-1.14<br>(-1.41 to -0.87)	-1.16<br>(-1.45 to -0.86)	-1.08<br>(-1.34 to -0.82)	-2.32<br>(-2.45 to -2.18)	-2.22<br>(-2.36 to -2.07)	-2.36<br>(-2.51 to -2.21)	-2.48<br>(-2.62 to -2.34)	-2.49<br>(-2.64 to -2.34)	-2.44<br>(-2.58 to -2.29)
France	0.09<br>(0.04 to 0.13)	-0.20<br>(-0.23 to -0.16)	0.37<br>(0.30 to 0.44)	-0.70<br>(-1.12 to -0.27)	-0.46<br>(-0.96 to 0.04)	-0.79<br>(-1.19 to -0.39)	-0.77<br>(-1.10 to -0.43)	-0.75<br>(-1.11 to -0.39)	-0.73<br>(-1.05 to -0.41)
Gabon	-0.81<br>(-0.88 to -0.73)	-0.79<br>(-0.86 to -0.72)	-0.80<br>(-0.89 to -0.71)	-1.10<br>(-1.23 to -0.97)	-1.28<br>(-1.33 to -1.24)	-0.90<br>(-1.14 to -0.66)	-1.01<br>(-1.14 to -0.89)	-1.14<br>(-1.19 to -1.08)	-0.87<br>(-1.10to -0.65)
Gambia	-0.36<br>(-0.40 to -0.31)	-0.34<br>(-0.39 to -0.28)	-0.34<br>(-0.38 to -0.29)	-0.12<br>(-0.28 to 0.05)	-0.33<br>(-0.50 to -0.16)	0.24<br>(0.06 to 0.41)	-0.21<br>(-0.40 to -0.03)	-0.37<br>(-0.57 to -0.18)	0.12<br>(-0.06 to 0.31)
Georgia	0.83<br>(0.73 − 0.94)	0.88<br>(0.75 − 1.01)	0.66<br>(0.51 − 0.80)	2.74<br>(1.79 − 3.69)	2.57<br>(1.59 − 3.56)	2.87<br>(1.93 − 3.82)	1.82<br>(1.09 − 2.55)	1.87<br>(1.11 − 2.65)	1.65<br>(0.94 − 2.36)
Germany	-1.05<br>(-1.10 to -1.00)	-0.69<br>(-0.75 to -0.63)	-1.28<br>(-1.35 to -1.20)	-2.00<br>(-2.18 to -1.82)	-1.56<br>(-1.74 to -1.37)	-2.14<br>(-2.33 to -1.95)	-2.23<br>(-2.36 to -2.10)	-2.11<br>(-2.24 to -1.99)	-2.23<br>(-2.37 to -2.08)
Ghana	-0.29<br>(-0.36 to -0.22)	0.01<br>(-0.05 to 0.06)	-0.57<br>(-0.66 to -0.48)	-0.36<br>(-0.59 to -0.14)	-0.15<br>(-0.35 to 0.04)	-0.57<br>(-0.85 to -0.29)	-0.51<br>(-0.70 to -0.32)	-0.25<br>(-0.40 to -0.10)	-0.78<br>(-1.01 to -0.54)
Greece	0.20<br>(0.10 to 0.31)	0.32<br>(0.21 to 0.43)	0.13<br>(-0.01 to 0.28)	-0.71<br>(-0.97 to -0.45)	-0.32<br>(-0.60 to -0.05)	-0.97<br>(-1.22 to -0.73)	-0.55<br>(-0.75 to -0.35)	-0.28<br>(-0.51 to -0.06)	-0.78<br>(-0.97 to -0.58)
Greenland	0.14<br>(0.02 to 0.26)	0.53<br>(0.30 to 0.76)	-0.11<br>(-0.25 to 0.04)	-3.27<br>(-3.40 to -3.15)	-2.44<br>(-2.54 to -2.34)	-3.65<br>(-3.83 to -3.47)	-3.39<br>(-3.51 to -3.27)	-2.79<br>(-2.90 to -2.67)	-3.74<br>(-3.92 to -3.55)
Grenada	-0.86<br>(-0.93 to -0.79)	-0.69<br>(-0.74 to -0.63)	-0.71<br>(-0.82 to -0.61)	-1.44<br>(-1.58 to -1.29)	-1.39<br>(-1.86 to -0.92)	-1.16<br>(-1.43 to -0.88)	-1.62<br>(-1.77 to -1.46)	-1.66<br>(-1.92 to -1.39)	-1.46<br>(-1.72 to -1.21)

Guam	-0.79<br>(-0.85 to -0.72)	-0.39<br>(-0.42 to -0.36)	-1.17<br>(-1.27 to -1.06)	-2.21<br>(-2.50 to -1.92)	-1.01<br>(-1.24 to -0.78)	-3.15<br>(-3.50 to -2.79)	-1.08<br>(-1.25 to -0.90)	-0.55<br>(-0.71 to -0.40)	-1.60<br>(-1.82 to -1.37)
Guatemala	-0.04<br>(-0.07 to -0.01)	0.22<br>(0.20 to 0.24)	-0.27<br>(-0.31 to -0.23)	0.57<br>(0.27 to 0.86)	0.82<br>(0.47 to 1.16)	0.32<br>(0.05 to 0.58)	0.38<br>(0.10 to 0.66)	0.71<br>(0.37 to 1.05)	0.07<br>(-0.18 to 0.32)
Guinea	-0.22<br>(-0.25 to -0.19)	-0.06<br>(-0.10 to -0.01)	-0.42<br>(-0.44 to -0.39)	0.05<br>(-0.09 to 0.19)	0.07<br>(-0.11 to 0.26)	0.03<br>(-0.09 to 0.15)	0.02<br>(-0.11 to 0.15)	0.09<br>(-0.08 to 0.26)	-0.05<br>(-0.16 to 0.06)
Guinea-Bissau	-0.56<br>(-0.62 to -0.50)	-0.45<br>(-0.51 to -0.38)	-0.65<br>(-0.71 to -0.60)	-0.43<br>(-0.52 to -0.34)	-0.71<br>(-0.79 to -0.62)	-0.12<br>(-0.21 to -0.02)	-0.53<br>(-0.61 to -0.44)	-0.70<br>(-0.79 to -0.62)	-0.31<br>(-0.40 to -0.21)
Guyana	-1.03<br>(-1.14 to -0.92)	-1.16<br>(-1.29 to -1.04)	-0.91<br>(-1 to -0.82)	-0.79<br>(-0.95 to -0.62)	-0.99<br>(-1.21 to -0.77)	-0.53<br>(-0.72 to -0.34)	-0.79<br>(-0.95 to -0.64)	-1.02<br>(-1.24 to -0.80)	-0.51<br>(-0.70 to -0.32)
Haiti	-0.28<br>(-0.36 to -0.20)	-0.23<br>(-0.34 to -0.11)	-0.35<br>(-0.41 to -0.29)	-0.69<br>(-0.76 to -0.62)	-0.81<br>(-0.94 to -0.67)	-0.66<br>(-0.75 to -0.57)	-0.81<br>(-0.89 to -0.72)	-0.83<br>(-0.99 to -0.68)	-0.81<br>(-0.89 to -0.73)
Honduras	0.02<br>(-0.01 to 0.06)	-0.07<br>(-0.10 to -0.04)	0.06<br>(-0.02 to 0.14)	1.05<br>(0.88 to 1.23)	0.49<br>(0.39 to 0.60)	1.38<br>(1.11 to 1.66)	0.16<br>(0.03 to 0.28)	-0.33<br>(-0.39 to -0.27)	0.44<br>(0.24 to 0.63)
Hungary	-1.06<br>(-1.13 to -0.99)	-0.81<br>(-0.86 to -0.76)	-1.25<br>(-1.33 to -1.16)	-2.01<br>(-2.25 to -1.76)	-1.79<br>(-2.01 to -1.58)	-2.16<br>(-2.43 to -1.88)	-2.38<br>(-2.56 to -2.19)	-2.23<br>(-2.39 to -2.07)	-2.48<br>(-2.70 to -2.25)
Iceland	-1.19<br>(-1.26 to -1.12)	-0.49<br>(-0.52 to -0.45)	-1.74<br>(-1.86 to -1.62)	-1.82<br>(-2.09 to -1.56)	-1.05<br>(-1.33 to -0.78)	-2.35<br>(-2.64 to -2.06)	-2.22<br>(-2.37 to -2.07)	-1.53<br>(-1.67 to -1.39)	-2.75<br>(-2.94 to -2.56)
India	-1.30<br>(-1.43 to -1.17)	-1.18<br>(-1.30 to -1.07)	-1.41<br>(-1.57 to -1.25)	-1.37<br>(-1.44 to -1.29)	-1.46<br>(-1.52 to -1.41)	-1.13<br>(-1.25 to -1.01)	-1.38<br>(-1.44 to -1.32)	-1.45<br>(-1.50 to -1.40)	-1.23<br>(-1.33 to -1.12)
Indonesia	-0.83<br>(-0.88 to -0.77)	-0.68<br>(-0.73 to -0.64)	-0.95<br>(-1.02 to -0.87)	-0.81<br>(-0.93 to -0.70)	-0.76<br>(-0.88 to -0.65)	-0.88<br>(-1.01 to -0.76)	-1.02<br>(-1.12 to -0.91)	-0.86<br>(-0.96 to -0.75)	-1.16<br>(-1.27 to -1.05)

Iran (Islamic Republic of Iran)	-1.43 (-1.54 to -1.32)	-1.49 (-1.61 to -1.38)	-1.39 (-1.50 to -1.27)	-3.23 (-3.35 to -3.10)	-3.42 (-3.53 to -3.32)	-2.99 (-3.15 to -2.82)	-3.04 (-3.14 to -2.95)	-3.06 (-3.12 to -3.00)	-3.02 (-3.17 to -2.87)
Iraq	-2.17 (-2.38 to -1.96)	-1.83 (-2.00 to -1.65)	-2.42 (-2.66 to -2.19)	-2.68 (-2.92 to -2.43)	-2.86 (-3.12 to -2.60)	-2.48 (-2.72 to -2.25)	-2.86 (-3.10 to -2.63)	-2.97 (-3.21 to -2.72)	-2.76 (-2.98 to -2.53)
Ireland	-1.99 (-2.13 to -1.85)	-1.51 (-1.61 to -1.40)	-2.30 (-2.46 to -2.14)	-2.43 (-2.62 to -2.23)	-1.61 (-1.80 to -1.42)	-2.94 (-3.16 to -2.73)	-2.91 (-3.11 to -2.71)	-2.24 (-2.46 to -2.03)	-3.38 (-3.60 to -3.16)
Israel	-0.76 (-0.82 to -0.70)	-0.46 (-0.51 to -0.42)	-0.97 (-1.04 to -0.90)	-0.97 (-1.27 to -0.66)	0.08 (-0.20 to 0.35)	-1.97 (-2.34 to -1.60)	-1.67 (-1.84 to -1.50)	-1.02 (-1.19 to -0.85)	-2.29 (-2.47 to -2.10)
Italy	-1.16 (-1.25 to -1.07)	-1.15 (-1.22 to -1.08)	-1.14 (-1.25 to -1.03)	-0.82 (-1.12 to -0.52)	-0.17 (-0.55 to 0.21)	-1.21 (-1.47 to -0.94)	-1.70 (-1.87 to -1.52)	-1.38 (-1.62 to -1.15)	-1.91 (-2.05 to -1.77)
Jamaica	-0.48 (-0.60 to -0.36)	-0.43 (-0.54 to -0.31)	-0.50 (-0.63 to -0.38)	-0.61 (-1.00 to -0.22)	-0.51 (-1.04 to 0.02)	-0.67 (-1.01 to -0.32)	-1.06 (-1.47 to -0.65)	-1.22 (-1.75 to -0.69)	-0.93 (-1.29 to -0.56)
Japan	-0.21 (-0.31 to -0.11)	-0.01 (-0.12 to 0.10)	-0.26 (-0.37 to -0.15)	-2.36 (-2.52 to -2.21)	-1.85 (-2.03 to -1.67)	-2.74 (-2.88 to -2.59)	-1.91 (-2.02 to -1.80)	-1.58 (-1.72 to -1.43)	-2.18 (-2.28 to -2.08)
Jordan	-1.31 (-1.43 to -1.18)	-1.11 (-1.22 to -1.01)	-1.53 (-1.68 to -1.38)	-2.60 (-2.78 to -2.41)	-2.55 (-2.66 to -2.44)	-2.57 (-2.90 to -2.23)	-2.37 (-2.50 to -2.23)	-2.24 (-2.33 to -2.15)	-2.46 (-2.67 to -.26)
Kazakhstan	0.08 (0.04 to 0.12)	0.32 (0.28 to 0.36)	-0.18 (-0.23 to -0.13)	0.03 (-0.20 to 0.25)	0.23 (-0.05 to 0.52)	-0.22 (-0.46 to 0.02)	-0.47 (-0.76 to -0.18)	-0.15 (-0.48 to 0.17)	-0.83 (-1.13 to -0.54)
Kenya	-0.46 (-0.55 to -0.37)	-0.33 (-0.46 to -0.21)	-0.53 (-0.59 to -0.47)	0.01 (-0.18 to 0.19)	-0.10 (-0.37 to 0.18)	0.47 (0.22 − 0.72)	0.04 (-0.17 to 0.24)	-0.04 (-0.32 to 0.23)	0.24 (0.05 to 0.44)
Kiribati	0.27 (0.19 − 0.35)	0.80 (0.66 − 0.94)	-0.22 (-0.27 to -0.17)	-0.25 (-0.31 to -0.19)	-0.36 (-0.43 to -0.29)	-0.09 (-0.15 to -0.03)	-0.33 (-0.39 to -0.27)	-0.27 (-0.34 to -0.19)	-0.44 (-0.48 to -0.40)

Kuwait	-0.62<br>(-0.69 to -0.55)	-0.66<br>(-0.73 to -0.58)	-0.79<br>(-0.86 to -0.71)	-4.64<br>(-5.44 to -3.83)	-3.17<br>(-3.91 to -2.41)	-7.04<br>(-7.96 to -6.11)	-3.67<br>(-4.25 to -3.09)	-2.70<br>(-3.26 to -2.15)	-5.01<br>(-5.67 to -4.34)
Kyrgyzstan	-0.66<br>(-0.77 to -0.56)	-0.37<br>(-0.48 to -0.27)	-1.01<br>(-1.12 to -0.90)	-0.43<br>(-0.80 to -0.06)	-0.54<br>(-0.84 to -0.24)	-0.46<br>(-0.90 to -0.03)	-0.78<br>(-1.20 to -0.36)	-0.71<br>(-1.10 to -0.31)	-0.93<br>(-1.36 to -0.49)
Lao People's Democratic Republic	-1.19<br>(-1.30 to -1.09)	-1.16<br>(-1.25 to -1.06)	-1.24<br>(-1.36 to -1.13)	-2.85<br>(-2.94 to -2.77)	-2.82<br>(-2.90 to -2.74)	-2.91<br>(-3.00 to -2.82)	-2.81<br>(-2.89 to -2.73)	-2.66<br>(-2.73 to -2.59)	-2.96<br>(-3.05 to -2.87)
Latvia	-1.57<br>(-1.69 to -1.44)	-1.44<br>(-1.57 to -1.32)	-1.68<br>(-1.81 to -1.55)	-1.79<br>(-2.33 to -1.25)	-1.56<br>(-2 to -1.11)	-2.03<br>(-2.67 to -1.39)	-2.09<br>(-2.56 to -1.63)	-1.91<br>(-2.33 to -1.49)	-2.34<br>(-2.89 to -1.79)
Lebanon	-1.71<br>(-1.83 to -1.59)	-1.80<br>(-1.93 to -1.68)	-1.63<br>(-1.75 to -1.51)	-4.88<br>(-5.17 to -4.58)	-4.94<br>(-5.29 to -4.60)	-4.87<br>(-5.13 to -4.61)	-4.57<br>(-4.88 to -4.26)	-4.55<br>(-4.89 to -4.21)	-4.62<br>(-4.91 to -4.32)
Lesotho	0.54<br>(0.44 − 0.64)	0.21<br>(0.15 − 0.28)	0.74<br>(0.62 − 0.86)	1.66<br>(1.25 − 2.07)	0.53<br>(0.27 − 0.80)	2.62<br>(2.03 − 3.23)	1.76<br>(1.37 − 2.16)	0.90<br>(0.60 − 1.19)	2.53<br>(1.98 − 3.08)
Liberia	-0.60<br>(-0.65 to -0.55)	-0.58<br>(-0.63 to -0.53)	-0.64<br>(-0.69 to -0.58)	-0.49<br>(-0.67 to -0.32)	-0.67<br>(-0.90 to -0.44)	-0.32<br>(-0.46 to -0.18)	-0.51<br>(-0.67 to -0.35)	-0.65<br>(-0.84 to -0.46)	-0.37<br>(-0.50 to -0.23)
Libya	-1.17<br>(-1.28 to -1.05)	-1.10<br>(-1.20 to -1.00)	-1.25<br>(-1.38 to -1.12)	-0.41<br>(-0.66 to -0.16)	-1.05<br>(-1.41 to -0.69)	-0.03<br>(-0.22 to 0.16)	-0.58<br>(-0.85 to -0.30)	-0.94<br>(-1.31 to -0.58)	-0.38<br>(-0.61 to -0.16)
Lithuania	-0.72<br>(-0.81 to -0.63)	-0.70<br>(-0.78 to -0.62)	-0.73<br>(-0.84 to -0.62)	-0.57<br>(-1.23 to 0.10)	-0.51<br>(-1.04 to 0.02)	-0.61<br>(-1.41 to 0.20)	-0.92<br>(-1.50 to -0.34)	-0.88<br>(-1.38 to -0.38)	-0.99<br>(-1.68 to -0.29)
Luxembourg	-1.41<br>(-1.67 to -1.15)	-1.29<br>(-1.53 to -1.04)	-1.48<br>(-1.76 to -1.20)	-2.43<br>(-2.73 to -2.14)	-1.82<br>(-2.07 to -1.57)	-2.76<br>(-3.10 to -2.42)	-2.98<br>(-3.20 to -2.75)	-2.70<br>(-2.90 to -2.50)	-3.13<br>(-3.40 to -2.87)

Madagascar	-0.25<br>(-0.29 to -0.21)	-0.11<br>(-0.15 to -0.08)	-0.39<br>(-0.44 to -0.34)	-0.71<br>(-0.76 to -0.66)	-0.82<br>(-0.86 to -0.78)	-0.37<br>(-0.43 to -0.32)	-0.63<br>(-0.68 to -0.59)	-0.72<br>(-0.76 to -0.68)	-0.41<br>(-0.46 to -0.36)
Malawi	-0.44<br>(-0.48 to -0.39)	-0.22<br>(-0.28 to -0.16)	-0.63<br>(-0.69 to -0.57)	-0.49<br>(-0.66 to -0.32)	-0.27<br>(-0.48 to -0.07)	-0.66<br>(-0.79 to -0.53)	-0.42<br>(-0.59 to -0.25)	-0.15<br>(-0.36 to 0.05)	-0.76<br>(-0.88 to -0.64)
Malaysia	-1.48<br>(-1.58 to -1.39)	-1.43<br>(-1.54 to -1.31)	-1.50<br>(-1.59 to -1.41)	-1.56<br>(-1.68 to -1.45)	-2.10<br>(-2.25 to -1.94)	-1.11<br>(-1.24 to -0.99)	-1.55<br>(-1.68 to -1.41)	-1.79<br>(-1.97 to -1.61)	-1.30<br>(-1.43 to -1.18)
Maldives	-2.10<br>(-2.25 to -1.94)	-1.82<br>(-1.96 to -1.68)	-2.45<br>(-2.62 to -2.28)	-4.08<br>(-4.22 to -3.94)	-3.90<br>(-4.00 to -3.81)	-4.47<br>(-4.65 to -4.28)	-4.09<br>(-4.25 to -3.93)	-3.75<br>(-3.86 to -3.65)	-4.60<br>(-4.80 to -4.39)
Mali	-0.64<br>(-0.69 to -0.60)	-0.50<br>(-0.53 to -0.46)	-0.90<br>(-0.97 to -0.83)	-0.78<br>(-0.89 to -0.67)	-0.73<br>(-0.89 to -0.56)	-0.81<br>(-0.88 to -0.74)	-0.75<br>(-0.84 to -0.65)	-0.61<br>(-0.75 to -0.47)	-0.85<br>(-0.91 to -0.79)
Malta	-1.15<br>(-1.28 to -1.01)	-0.95<br>(-1.07 to -0.82)	-1.25<br>(-1.39 to -1.10)	-1.62<br>(-1.89 to -1.34)	-1.10<br>(-1.41 to -0.79)	-1.96<br>(-2.23 to -1.70)	-1.48<br>(-1.64 to -1.32)	-1.20<br>(-1.39 to -1.00)	-1.65<br>(-1.81 to -1.50)
Marshall Islands	-0.16<br>(-0.24 to -0.08)	0.01<br>(-0.06 to 0.09)	-0.21<br>(-0.31 to -0.11)	-1.04<br>(-1.13 to -0.96)	-0.93<br>(-0.99 to -0.87)	-0.89<br>(-1.05 to -0.73)	-0.73<br>(-0.84 to -0.63)	-0.72<br>(-0.78 to -0.65)	-0.63<br>(-0.82 to -0.44)
Mauritania	-0.87<br>(-0.93 to -0.81)	-0.88<br>(-0.94 to -0.82)	-0.89<br>(-0.96 to -0.82)	-1.43<br>(-1.54 to -1.32)	-1.64<br>(-1.76 to -1.53)	-1.18<br>(-1.28 to -1.07)	-1.43<br>(-1.52 to -1.34)	-1.63<br>(-1.72 to -1.53)	-1.19<br>(-1.27 to -1.10)
Mauritius	-0.77<br>(-0.85 to -0.69)	-0.70<br>(-0.75 to -0.64)	-0.83<br>(-0.93 to -0.73)	-1.37<br>(-1.71 to -1.02)	-1.18<br>(-1.52 to -0.84)	-1.52<br>(-1.94 to -1.09)	-1.00<br>(-1.31 to -0.70)	-0.87<br>(-1.21 to -0.54)	-1.13<br>(-1.48 to -0.78)
Mexico	-0.03<br>(-0.07 to 0.02)	0.21<br>(0.15 to 0.26)	-0.21<br>(-0.25 to -0.17)	1.09<br>(0.92 to 1.26)	1.85<br>(1.67 to 2.03)	0.48<br>(0.30 to 0.66)	0.68<br>(0.57 to 0.80)	1.42<br>(1.29 to 1.54)	0.08<br>(-0.04 to 0.20)

Micronesia (Federated States of Micronesia)	-0.28 (-0.34 to -0.22)	-0.15 (-0.22 to -0.08)	-0.44 (-0.50 to -0.38)	-1.29 (-1.40 to -1.18)	-1.28 (-1.37 to -1.19)	-1.36 (-1.48 to -1.24)	-1.19 (-1.28 to -1.11)	-1.15 (-1.22 to -1.07)	-1.29 (-1.39 to -1.19)
Monaco	-1.29 (-1.40 to -1.18)	-1.24 (-1.35 to -1.13)	-1.33 (-1.44 to -1.22)	-2.98 (-3.11 to -2.85)	-3.18 (-3.36 to -3.00)	-2.83 (-2.94 to -2.71)	-2.69 (-2.77 to -2.60)	-2.77 (-2.91 to -2.63)	-2.61 (-2.70 to -2.53)
Mongolia	0.34 (0.19 to 0.49)	0.74 (0.61 to 0.88)	-0.04 (-0.23 to 0.16)	-0.11 (-0.36 to 0.14)	-0.35 (-0.65 to -0.05)	-0.02 (-0.25 to 0.20)	-0.12 (-0.37 to 0.13)	-0.02 (-0.31 to 0.28)	-0.30 (-0.51 to -0.09)
Montenegro	-0.33 (-0.42 to -0.25)	-0.29 (-0.38 to -0.21)	-0.36 (-0.45 to -0.27)	-0.51 (-0.81 to -0.21)	-1.19 (-1.47 to -0.91)	-0.12 (-0.47 to 0.23)	-1.04 (-1.37 to -0.70)	-1.51 (-1.80 to -1.23)	-0.71 (-1.11 to -0.31)
Morocco	-1.09 (-1.17 to -1.02)	-1.02 (-1.08 to -0.95)	-1.14 (-1.23 to -1.06)	-1.20 (-1.26 to -1.13)	-1.37 (-1.46 to -1.29)	-1.06 (-1.12 to -0.99)	-1.73 (-1.77 to -1.68)	-1.90 (-1.94 to -1.85)	-1.60 (-1.65 to -1.55)
Mozambique	0.03 (0.01 − 0.06)	0.24 (0.21 − 0.28)	-0.20 (-0.24 to -0.15)	0.64 (0.46 − 0.81)	0.89 (0.68 − 1.10)	0.34 (0.12 − 0.56)	0.83 (0.64 − 1.02)	1.14 (0.92 − 1.36)	0.26 (0.07 − 0.45)
Myanmar	-1.52 (-1.68 to -1.35)	-1.33 (-1.48 to -1.18)	-1.70 (-1.88 to -1.52)	-2.71 (-2.87 to -2.55)	-2.37 (-2.49 to -2.25)	-3.04 (-3.24 to -2.84)	-2.69 (-2.85 to -2.53)	-2.29 (-2.41 to -2.16)	-3.14 (-3.35 to -2.94)
Namibia	-0.77 (-0.87 to -0.68)	-0.66 (-0.75 to -0.57)	-0.86 (-0.95 to -0.76)	-0.97 (-1.25 to -0.70)	-0.76 (-1.03 to -0.50)	-1.15 (-1.43 to -0.87)	-0.93 (-1.19 to -0.67)	-0.72 (-1 to -0.45)	-1.14 (-1.38 to -0.90)
Nauru	-0.36 (-0.48 to -0.24)	-0.05 (-0.20 to 0.09)	-0.88 (-0.96 to -0.80)	-0.47 (-0.93 to 0)	-0.56 (-1.08 to -0.02)	-0.85 (-1.24 to -0.46)	-0.42 (-0.91 to 0.08)	-0.42 (-0.94 to 0.10)	-0.61 (-1.08 to -0.15)
Nepal	-0.79 (-0.87 to -0.72)	-0.76 (-0.82 to -0.71)	-0.82 (-0.91 to -0.72)	-1.54 (-1.78 to -1.29)	-1.24 (-1.49 to -1.00)	-1.84 (-2.10 to -1.58)	-1.62 (-1.82 to -1.41)	-1.28 (-1.49 to -1.07)	-1.96 (-2.18 to -1.75)

Netherlands	-1.21<br>(-1.28 to -1.14)	-0.86<br>(-0.92 to -0.81)	-1.42<br>(-1.50 to -1.34)	-2.39<br>(-2.66 to -2.12)	-1.71<br>(-2.01 to -1.40)	-2.75<br>(-3.04 to -2.46)	-2.78<br>(-3.03 to -2.53)	-2.29<br>(-2.51 to -2.08)	-3.08<br>(-3.38 to -2.78)
New Zealand	-1.65<br>(-1.82 to -1.49)	-1.33<br>(-1.45 to -1.20)	-1.83<br>(-2.03 to -1.64)	-2.31<br>(-2.53 to -2.10)	-1.87<br>(-2.16 to -1.59)	-2.58<br>(-2.78 to -2.37)	-2.57<br>(-2.75 to -2.38)	-2.15<br>(-2.39 to -1.90)	-2.85<br>(-3.02 to -2.68)
Nicaragua	-0.81<br>(-0.88 to -0.75)	-0.89<br>(-0.97 to -0.82)	-0.80<br>(-0.86 to -0.74)	-0.89<br>(-1.08 to -0.69)	-1.17<br>(-1.35 to -0.99)	-0.80<br>(-1.01 to -0.60)	-1.08<br>(-1.25 to -0.91)	-1.16<br>(-1.33 to -1.00)	-1.07<br>(-1.25 to -0.90)
Niger	-0.44<br>(-0.49 to -0.39)	-0.37<br>(-0.42 to -0.32)	-0.50<br>(-0.55 to -0.45)	-0.54<br>(-0.59 to -0.49)	-0.48<br>(-0.54 to -0.42)	-0.59<br>(-0.65 to -0.54)	-0.71<br>(-0.77 to -0.65)	-0.62<br>(-0.69 to -0.55)	-0.77<br>(-0.84 to -0.70)
Nigeria	-1.30<br>(-1.43 to -1.17)	-1.51<br>(-1.66 to -1.36)	-0.99<br>(-1.09 to -0.89)	-2.74<br>(-2.99 to -2.49)	-2.83<br>(-3.08 to -2.57)	-2.30<br>(-2.51 to -2.08)	-2.33<br>(-2.55 to -2.12)	-2.31<br>(-2.53 to -2.10)	-1.95<br>(-2.14 to -1.77)
Niue	-1.20<br>(-1.28 to -1.11)	-1.26<br>(-1.36 to -1.15)	-1.10<br>(-1.17 to -1.03)	-1.79<br>(-1.99 to -1.60)	-1.86<br>(-2.04 to -1.67)	-1.67<br>(-1.88 to -1.46)	-1.39<br>(-1.66 to -1.12)	-1.51<br>(-1.73 to -1.29)	-1.22<br>(-1.57 to -0.88)
North Macedonia	0.14<br>(0.02 to 0.25)	-0.06<br>(-0.17 to 0.05)	0.22<br>(0.10 − 0.35)	-0.78<br>(-1.15 to -0.40)	-1.25<br>(-1.62 to -0.89)	-0.54<br>(-0.93 to -0.15)	-1.30<br>(-1.60 to -1.00)	-1.58<br>(-1.88 to -1.27)	-1.12<br>(-1.42 to -0.82)
Northern Mariana Islands	-0.57<br>(-0.61 to -0.53)	-0.25<br>(-0.26 to -0.23)	-0.99<br>(-1.06 to -0.93)	-1.33<br>(-1.48 to -1.18)	-0.13<br>(-0.29 to 0.03)	-2.24<br>(-2.44 to -2.03)	-1.23<br>(-1.34 to -1.11)	-0.33<br>(-0.46 to -0.20)	-2.17<br>(-2.35 to -2.00)
Norway	-1.22<br>(-1.36 to -1.07)	-1.06<br>(-1.19 to -0.94)	-1.28<br>(-1.44 to -1.11)	-2.87<br>(-3.17 to -2.57)	-2.09<br>(-2.48 to -1.70)	-3.37<br>(-3.65 to -3.09)	-3.21<br>(-3.43 to -2.99)	-2.71<br>(-2.99 to -2.44)	-3.58<br>(-3.80 to -3.37)
Oman	0.12<br>(0.06 − 0.19)	-0.04<br>(-0.13 to 0.05)	0.11<br>(0.03 − 0.18)	-2.79<br>(-2.97 to -2.61)	-2.83<br>(-3.06 to -2.60)	-2.86<br>(-3.01 to -2.71)	-2.71<br>(-2.83 to -2.60)	-2.77<br>(-2.92 to -2.61)	-2.77<br>(-2.85 to -2.69)
Pakistan	-0.82<br>(-0.95 to -0.69)	-0.79<br>(-0.91 to -0.67)	-0.84<br>(-0.98 to -0.69)	-0.83<br>(-1.08 to -0.58)	-0.97<br>(-1.25 to -0.69)	-0.65<br>(-0.87 to -0.44)	-0.61<br>(-0.84 to -0.38)	-0.68<br>(-0.94 to -0.41)	-0.54<br>(-0.73 to -0.34)

Palau	-0.87<br>(-0.93 to -0.81)	-0.66<br>(-0.71 to -0.61)	-1.03<br>(-1.09 to -0.97)	-0.82<br>(-0.93 to -0.71)	-1.16<br>(-1.28 to -1.05)	-0.38<br>(-0.52 to -0.24)	-0.75<br>(-0.83 to -0.66)	-0.89<br>(-1 to -0.79)	-0.67<br>(-0.77 to -0.57)
Palestine	-0.78<br>(-0.85 to -0.71)	-0.37<br>(-0.45 to -0.30)	-1.02<br>(-1.10 to -0.94)	-2.06<br>(-2.34 to -1.78)	-2.07<br>(-2.49 to -1.65)	-1.99<br>(-2.19 to -1.79)	-1.92<br>(-2.07 to -1.77)	-1.90<br>(-2.15 to -1.66)	-1.92<br>(-2.02 to -1.82)
Panama	-0.46<br>(-0.51 to -0.40)	-0.21<br>(-0.25 to -0.17)	-0.65<br>(-0.72 to -0.59)	0.16<br>(-0.02 to 0.34)	0.89<br>(0.73 to 1.05)	-0.44<br>(-0.67 to -0.22)	0.06<br>(-0.12 to 0.23)	0.64<br>(0.47 to 0.81)	-0.43<br>(-0.64 to -0.22)
Papua New Guinea	-0.65<br>(-0.70 to -0.60)	-0.59<br>(-0.64 to -0.54)	-0.67<br>(-0.73 to -0.61)	-1.25<br>(-1.28 to -1.22)	-1.15<br>(-1.23 to -1.07)	-1.25<br>(-1.30 to -1.20)	-1.14<br>(-1.18 to -1.09)	-1.03<br>(-1.08 to -0.97)	-1.19<br>(-1.28 to -1.09)
Paraguay	-0.46<br>(-0.49 to -0.43)	-0.49<br>(-0.52 to -0.46)	-0.45<br>(-0.50 to -0.41)	-0.48<br>(-0.63 to -0.32)	-0.65<br>(-0.80 to -0.49)	-0.38<br>(-0.55 to -0.21)	-0.79<br>(-0.93 to -0.65)	-0.84<br>(-0.98 to -0.71)	-0.77<br>(-0.92 to -0.62)
Peru	-1.29<br>(-1.39 to -1.19)	-1.15<br>(-1.24 to -1.05)	-1.41<br>(-1.53 to -1.30)	-1.83<br>(-2.15 to -1.50)	-1.98<br>(-2.26 to -1.71)	-1.71<br>(-2.08 to -1.34)	-1.80<br>(-2.08 to -1.52)	-1.85<br>(-2.09 to -1.60)	-1.76<br>(-2.09 to -1.44)
Philippines	1.17<br>(0.96 − 1.37)	1.08<br>(0.89 − 1.27)	1.26<br>(1.04 − 1.47)	0.69<br>(0.53 − 0.86)	0.58<br>(0.40 to 0.77)	0.68<br>(0.52 − 0.84)	0.84<br>(0.66 − 1.02)	0.74<br>(0.54 − 0.95)	0.88<br>(0.73 − 1.04)
Poland	-1.97<br>(-2.14 to -1.80)	-1.69<br>(-1.85 to -1.54)	-2.20<br>(-2.39 to -2.02)	-2.35<br>(-2.68 to -2.01)	-2.16<br>(-2.42 to -1.89)	-2.56<br>(-2.97 to -2.15)	-2.57<br>(-2.86 to -2.28)	-2.37<br>(-2.61 to -2.12)	-2.80<br>(-3.16 to -2.45)
Portugal	-1.51<br>(-1.62 to -1.40)	-1.29<br>(-1.38 to -1.20)	-1.69<br>(-1.82 to -1.56)	-1.44<br>(-1.66 to -1.21)	-1.16<br>(-1.41 to -0.91)	-1.59<br>(-1.80 to -1.39)	-2.01<br>(-2.19 to -1.83)	-1.93<br>(-2.15 to -1.72)	-2.04<br>(-2.21 to -1.87)
Puerto Rico	-0.89<br>(-0.99 to -0.80)	-0.70<br>(-0.77 to -0.62)	-1.00<br>(-1.10 to -0.90)	-2.04<br>(-2.31 to -1.78)	-1.71<br>(-1.99 to -1.44)	-2.29<br>(-2.56 to -2.03)	-2.07<br>(-2.30 to -1.83)	-1.90<br>(-2.15 to -1.64)	-2.21<br>(-2.44 to -1.97)
Qatar	-0.37<br>(-0.41 to -0.33)	-0.47<br>(-0.57 to -0.36)	-0.50<br>(-0.66 to -0.34)	-4.00<br>(-4.54 to -3.46)	-4.26<br>(-4.84 to -3.69)	-3.33<br>(-3.90 to -2.75)	-3.58<br>(-3.98 to -3.18)	-3.84<br>(-4.27 to -3.41)	-3.13<br>(-3.51 to -2.75)

Republic of Korea	-2.12 (-2.50 to -1.74)	-1.79 (-2.10 to -1.48)	-2.24 (-2.67 to -1.80)	-4.78 (-4.97 to -4.58)	-4.36 (-4.65 to -4.08)	-4.94 (-5.11 to -4.76)	-4.41 (-4.63 to -4.19)	-4.11 (-4.36 to -3.86)	-4.54 (-4.76 to -4.32)
Republic of Moldova	-0.88 (-0.97 to -0.79)	-0.72 (-0.80 to -0.63)	-1.07 (-1.17 to -0.97)	-1.50 (-2.12 to -0.88)	-1.47 (-1.96 to -0.99)	-1.71 (-2.50 to -0.92)	-1.35 (-1.94 to -0.76)	-1.26 (-1.75 to -0.77)	-1.58 (-2.31 to -0.85)
Romania	-0.30 (-0.36 to -0.24)	-0.10 (-0.17 to -0.03)	-0.49 (-0.55 to -0.43)	-0.22 (-0.70 to 0.25)	0.13 (-0.31 to 0.58)	-0.57 (-1.07 to -0.07)	-0.59 (-1.01 to -0.17)	-0.21 (-0.62 to 0.21)	-0.98 (-1.41 to -0.55)
Russian Federation	-0.19 (-0.55 to 0.18)	-0.30 (-0.62 to 0.03)	-0.15 (-0.54 to 0.23)	-0.38 (-1.61 to 0.87)	-0.71 (-1.85 to 0.46)	-0.19 (-1.51 to 1.13)	-0.68 (-1.60 to 0.25)	-0.82 (-1.74 to 0.09)	-0.54 (-1.48 to 0.41)
Rwanda	-1.65 (-1.80 to -1.50)	-1.27 (-1.39 to -1.16)	-1.99 (-2.18 to -1.81)	-3.80 (-4.15 to -3.44)	-3.49 (-3.82 to -3.16)	-4.01 (-4.40 to -3.62)	-3.84 (-4.19 to -3.48)	-3.66 (-4.01 to -3.31)	-4.01 (-4.38 to -3.65)
Saint Kitts and Nevis	-1.37 (-1.48 to -1.25)	-0.94 (-1.03 to -0.85)	-1.65 (-1.78 to -1.53)	-2.17 (-2.42 to -1.92)	-1.81 (-2.07 to -1.55)	-2.61 (-2.87 to -2.35)	-2.49 (-2.75 to -2.22)	-1.93 (-2.22 to -1.64)	-3.00 (-3.25 to -2.75)
Saint Lucia	-1.04 (-1.13 to -0.95)	-0.72 (-0.77 to -0.66)	-1.19 (-1.31 to -1.07)	-2.62 (-2.84 to -2.40)	-2.16 (-2.36 to -1.96)	-2.86 (-3.11 to -2.61)	-2.39 (-2.58 to -2.21)	-2.03 (-2.20 to -1.86)	-2.58 (-2.80 to -2.36)
Saint Vincent and the Grenadines	-0.90 (-1.02 to -0.78)	-0.86 (-1.01 to -0.72)	-0.88 (-0.98 to -0.77)	-1.02 (-1.17 to -0.86)	-0.65 (-0.83 to -0.47)	-1.25 (-1.42 to -1.08)	-1.06 (-1.19 to -0.93)	-0.72 (-0.87 to -0.56)	-1.26 (-1.40 to -1.12)
Samoa	-0.46 (-0.50 to -0.42)	-0.56 (-0.61 to -0.51)	-0.33 (-0.37 to -0.30)	-1.17 (-1.39 to -0.95)	-1.72 (-1.99 to -1.45)	-0.70 (-0.87 to -0.52)	-0.93 (-1.13 to -0.74)	-1.35 (-1.60 to -1.11)	-0.49 (-0.64 to -0.35)
San Marino	-0.79 (-0.86 to -0.71)	-0.91 (-1 to -0.83)	-0.71 (-0.77 to -0.64)	-2.59 (-2.89 to -2.29)	-2.56 (-2.91 to -2.21)	-2.57 (-2.83 to -2.30)	-2.04 (-2.23 to -1.84)	-2.18 (-2.40 to -1.95)	-1.89 (-2.06 to -1.71)
Sao Tome and Principe	-0.37 (-0.42 to -0.31)	-0.38 (-0.42 to -0.34)	-0.40 (-0.47 to -0.32)	-0.25 (-0.46 to -0.03)	-0.31 (-0.50 to -0.12)	-0.24 (-0.49 to 0.01)	-0.28 (-0.51 to -0.05)	-0.28 (-0.48 to -0.07)	-0.31 (-0.58 to -0.04)

Saudi Arabia	-0.92 (-0.99 to -0.85)	-1.14 (-1.23 to -1.05)	-0.69 (-0.74 to -0.65)	-1.43 (-1.63 to -1.22)	-1.79 (-1.96 to -1.62)	-1.06 (-1.30 to -0.81)	-1.42 (-1.59 to -1.25)	-1.72 (-1.87 to -1.57)	-1.13 (-1.33 to -0.93)
Senegal	-0.50 (-0.55 to -0.46)	-0.44 (-0.49 to -0.40)	-0.56 (-0.61 to -0.51)	-0.42 (-0.52 to -0.31)	-0.56 (-0.66 to -0.47)	-0.24 (-0.36 to -0.12)	-0.55 (-0.66 to -0.44)	-0.64 (-0.75 to -0.53)	-0.43 (-0.55 to -0.32)
Serbia	-1.04 (-1.18 to -0.91)	-0.97 (-1.09 to -0.85)	-1.18 (-1.34 to -1.02)	-2.72 (-3.03 to -2.41)	-2.47 (-2.74 to -2.20)	-2.83 (-3.16 to -2.50)	-2.87 (-3.17 to -2.57)	-2.69 (-2.98 to -2.41)	-2.97 (-3.29 to -2.66)
Seychelles	-1.20 (-1.29 to -1.10)	-1.45 (-1.57 to -1.33)	-0.96 (-1.04 to -0.88)	-1.96 (-2.17 to -1.74)	-2.57 (-2.80 to -2.33)	-1.50 (-1.72 to -1.27)	-1.80 (-1.98 to -1.63)	-2.27 (-2.45 to -2.08)	-1.36 (-1.55 to -1.18)
Sierra Leone	-0.26 (-0.31 to -0.21)	-0.33 (-0.39 to -0.28)	-0.16 (-0.21 to -0.11)	0 (-0.19 to 0.20)	-0.50 (-0.66 to -0.33)	0.56 (0.32 to 0.80)	-0.01 (-0.20 to 0.17)	-0.44 (-0.60 to -0.29)	0.50 (0.27 to 0.72)
Singapore	-1.63 (-1.75 to -1.52)	-1.22 (-1.31 to -1.13)	-1.83 (-1.95 to -1.70)	-4.19 (-4.39 to -3.98)	-3.91 (-4.07 to -3.75)	-4.29 (-4.52 to -4.06)	-3.51 (-3.62 to -3.41)	-3.37 (-3.45 to -3.30)	-3.54 (-3.66 to -3.41)
Slovakia	-1.96 (-2.10 to -1.81)	-2.00 (-2.18 to -1.81)	-1.89 (-2.00 to -1.78)	-1.96 (-2.04 to -1.87)	-2.06 (-2.15 to -1.97)	-1.88 (-1.98 to -1.77)	-2.02 (-2.11 to -1.92)	-2.17 (-2.29 to -2.05)	-1.87 (-1.97 to -1.77)
Slovenia	-1.50 (-1.63 to -1.37)	-1.30 (-1.42 to -1.18)	-1.64 (-1.77 to -1.50)	-2.93 (-3.23 to -2.62)	-2.82 (-3.07 to -2.57)	-3.00 (-3.35 to -2.64)	-3.26 (-3.51 to -3.00)	-3.25 (-3.48 to -3.02)	-3.24 (-3.53 to -2.94)
Solomon Islands	0.38 (0.26 − 0.50)	0.34 (0.25 − 0.43)	0.26 (0.10 − 0.42)	-0.64 (-0.75 to -0.53)	-0.57 (-0.65 to -0.48)	-0.88 (-1.01 to -0.76)	-0.50 (-0.60 to -0.40)	-0.41 (-0.49 to -0.33)	-0.70 (-0.83 to -0.56)
Somalia	-0.14 (-0.17 to -0.10)	0.03 (-0.02 to 0.07)	-0.24 (-0.28 to -0.20)	-1.05 (-1.12 to -0.99)	-0.89 (-0.95 to -0.84)	-0.84 (-0.93 to -0.75)	-1.01 (-1.08 to -0.94)	-0.92 (-0.97 to -0.86)	-0.88 (-0.98 to -0.79)
South Africa	-0.78 (-0.92 to -0.64)	-0.71 (-0.85 to -0.58)	-0.83 (-0.97 to -0.69)	0.36 (0.04 − 0.69)	0 (-0.36 to 0.37)	0.67 (0.35 − 0.98)	-0.14 (-0.40 to 0.12)	-0.36 (-0.66 to -0.06)	0.05 (-0.22 to 0.32)

South Sudan	-0.46<br>(-0.54 to -0.38)	-0.33<br>(-0.43 to -0.23)	-0.59<br>(-0.66 to -0.52)	-0.95<br>(-1.31 to -0.60)	-0.97<br>(-1.34 to -0.60)	-0.69<br>(-1.07 to -0.31)	-0.88<br>(-1.22 to -0.53)	-0.86<br>(-1.21 to -0.50)	-0.62<br>(-0.98 to -0.26)
Spain	-0.29<br>(-0.40 to -0.18)	-0.21<br>(-0.30 to -0.13)	-0.35<br>(-0.48 to -0.22)	0.04<br>(-0.35 to 0.42)	0.50<br>(0.12 to 0.89)	-0.21<br>(-0.60 to 0.18)	-0.87<br>(-1.11 to -0.63)	-0.80<br>(-1.04 to -0.56)	-0.85<br>(-1.10 to -0.60)
Sri Lanka	-1.28<br>(-1.4 to -1.17)	-1.30<br>(-1.43 to -1.16)	-1.22<br>(-1.32 to -1.12)	-1.49<br>(-1.69 to -1.30)	-1.66<br>(-1.88 to -1.44)	-1.33<br>(-1.54 to -1.11)	-1.53<br>(-1.65 to -1.42)	-1.75<br>(-1.91 to -1.58)	-1.31<br>(-1.42 to -1.20)
Sudan	-1.57<br>(-1.69 to -1.45)	-1.70<br>(-1.83 to -1.57)	-1.46<br>(-1.57 to -1.35)	-2.38<br>(-2.45 to -2.30)	-2.89<br>(-2.99 to -2.80)	-1.83<br>(-1.88 to -1.77)	-2.46<br>(-2.51 to -2.42)	-2.73<br>(-2.79 to -2.67)	-2.19<br>(-2.23 to -2.15)
Suriname	-0.52<br>(-0.57 to -0.47)	-0.41<br>(-0.46 to -0.36)	-0.61<br>(-0.66 to -0.56)	-0.91<br>(-1.07 to -0.75)	-0.76<br>(-0.91 to -0.61)	-1.04<br>(-1.23 to -0.86)	-1.02<br>(-1.17 to -0.87)	-0.97<br>(-1.10 to -0.83)	-1.07<br>(-1.25 to -0.88)
Sweden	-1.34<br>(-1.44 to -1.24)	-1.24<br>(-1.33 to -1.16)	-1.41<br>(-1.53 to -1.30)	-2.67<br>(-2.97 to -2.37)	-2.19<br>(-2.49 to -1.89)	-2.91<br>(-3.23 to -2.59)	-2.92<br>(-3.13 to -2.70)	-2.57<br>(-2.78 to -2.36)	-3.16<br>(-3.41 to -2.90)
Switzerland	-0.93<br>(-1.20 to -0.66)	-0.78<br>(-0.99 to -0.57)	-1.01<br>(-1.32 to -0.69)	-2.04<br>(-2.18 to -1.90)	-1.51<br>(-1.70 to -1.31)	-2.34<br>(-2.49 to -2.20)	-2.61<br>(-2.72 to -2.50)	-2.26<br>(-2.41 to -2.12)	-2.85<br>(-2.97 to -2.74)
Syrian Arab Republic	-1.25<br>(-1.37 to -1.13)	-1.15<br>(-1.28 to -1.03)	-1.33<br>(-1.46 to -1.20)	-2.35<br>(-2.68 to -2.02)	-2.32<br>(-2.64 to -2.00)	-2.33<br>(-2.67 to -1.98)	-2.69<br>(-3.03 to -2.34)	-2.61<br>(-2.95 to -2.28)	-2.73<br>(-3.10 to -2.36)
Taiwan (Province of China)	-1.97<br>(-2.11 to -1.83)	-2.11<br>(-2.26 to -1.96)	-1.86<br>(-1.98 to -1.74)	-3.06<br>(-3.44 to -2.67)	-1.47<br>(-1.98 to -0.95)	-2.69<br>(-3.04 to -2.34)	-3.69<br>(-3.97 to -3.40)	-3.92<br>(-4.24 to -3.60)	-3.43<br>(-3.67 to -3.19)
Tajikistan	-0.40<br>(-0.47 to -0.34)	-0.22<br>(-0.34 to -0.10)	-0.60<br>(-0.66 to -0.54)	-2.27<br>(-2.68 to -1.86)	-1.40<br>(-1.99 to -0.80)	-3.03<br>(-3.36 to -2.70)	-1.72<br>(-2.01 to -1.44)	-1.15<br>(-1.51 to -0.79)	-2.32<br>(-2.55 to -2.10)
Thailand	0.54<br>(0.44 to 0.63)	0.60<br>(0.49 to 0.72)	0.42<br>(0.34 to 0.49)	-1.17<br>(-1.67 to -0.67)	-2.43<br>(-2.59 to -2.27)	-1.09<br>(-1.53 to -0.65)	-1.29<br>(-1.65 to -0.93)	-1.22<br>(-1.65 to -0.78)	-1.42<br>(-1.72 to -1.13)

Timor-Leste	-1.69<br>(-1.83 to -1.54)	-1.51<br>(-1.65 to -1.38)	-1.90<br>(-2.06 to -1.74)	-2.66<br>(-2.88 to -2.44)	-0.56<br>(-0.76 to -0.35)	-2.96<br>(-3.25 to -2.67)	-2.19<br>(-2.39 to -1.98)	-1.83<br>(-2 to -1.66)	-2.69<br>(-2.95 to -2.42)
Togo	-0.51<br>(-0.58 to -0.43)	-0.51<br>(-0.59 to -0.43)	-0.52<br>(-0.59 to -0.44)	-0.72<br>(-0.93 to -0.51)	0.07<br>(-0.15 to 0.29)	-0.90<br>(-1.10 to -0.69)	-0.72<br>(-0.97 to -0.48)	-0.49<br>(-0.73 to -0.26)	-0.97<br>(-1.22 to -0.72)
Tokelau	-0.40<br>(-0.44 to -0.37)	-0.27<br>(-0.33 to -0.20)	-0.50<br>(-0.54 to -0.47)	-0.09<br>(-0.23 to 0.05)	-2.40<br>(-2.53 to -2.27)	-0.27<br>(-0.36 to -0.18)	-0.21<br>(-0.35 to -0.07)	-0.05<br>(-0.27 to 0.17)	-0.42<br>(-0.50 to -0.35)
Tonga	-1.20<br>(-1.29 to -1.12)	-1.01<br>(-1.08 to -0.94)	-1.29<br>(-1.39 to -1.20)	-2.29<br>(-2.43 to -2.15)	-0.89<br>(-0.96 to -0.81)	-2.23<br>(-2.37 to -2.08)	-1.84<br>(-2.09 to -1.60)	-1.94<br>(-2.14 to -1.73)	-1.81<br>(-2.10 to -1.51)
Trinidad and Tobago	-0.76<br>(-0.82 to -0.70)	-0.56<br>(-0.61 to -0.50)	-0.95<br>(-1.03 to -0.87)	-0.77<br>(-0.85 to -0.69)	-0.91<br>(-1.08 to -0.73)	-0.80<br>(-0.92 to -0.68)	-0.75<br>(-0.81 to -0.70)	-0.61<br>(-0.67 to -0.56)	-0.92<br>(-1.00 to -0.84)
Tunisia	-0.80<br>(-0.85 to -0.76)	-0.62<br>(-0.65 to -0.58)	-0.95<br>(-1.01 to -0.89)	-1.24<br>(-1.42 to -1.06)	-2.45<br>(-2.51 to -2.40)	-1.57<br>(-1.77 to -1.38)	-1.19<br>(-1.38 to -0.99)	-0.83<br>(-1.02 to -0.65)	-1.53<br>(-1.76 to -1.31)
Turkey	-1.26<br>(-1.34 to -1.17)	-1.23<br>(-1.32 to -1.14)	-1.26<br>(-1.35 to -1.17)	-2.10<br>(-2.14 to -2.06)	-3.43<br>(-3.80 to -3.06)	-1.77<br>(-1.83 to -1.71)	-2.22<br>(-2.26 to -2.17)	-2.38<br>(-2.43 to -2.32)	-2.09<br>(-2.15 to -2.03)
Turkmenistan	1.04<br>(0.93 − 1.15)	1.28<br>(1.15 − 1.42)	0.73<br>(0.64 − 0.81)	1.43<br>(1.09 − 1.77)	1.93<br>(1.57 − 2.28)	0.75<br>(0.41 − 1.10)	1.26<br>(0.95 − 1.57)	1.78<br>(1.45 − 2.12)	0.49<br>(0.19 − 0.79)
Tuvalu	-0.78<br>(-0.84 to -0.71)	-0.56<br>(-0.63 to -0.50)	-0.91<br>(-0.99 to -0.84)	-1.85<br>(-1.98 to -1.73)	-1.80<br>(-1.93 to -1.67)	-1.88<br>(-2.00 to -1.77)	-1.71<br>(-1.83 to -1.59)	-1.66<br>(-1.78 to -1.54)	-1.86<br>(-1.98 to -1.74)
Uganda	-0.63<br>(-0.70 to -0.56)	-0.60<br>(-0.68 to -0.52)	-0.57<br>(-0.65 to -0.50)	-2.22<br>(-2.41 to -2.02)	-2.14<br>(-2.32 to -1.97)	-1.85<br>(-2.12 to -1.58)	-1.97<br>(-2.17 to -1.77)	-1.97<br>(-2.16 to -1.77)	-1.67<br>(-1.93 to -1.41)
Ukraine	-0.33<br>(-0.40 to -0.26)	0.03<br>(-0.06 to 0.11)	-0.79<br>(-0.86 to -0.72)	-1.39<br>(-1.53 to -1.24)	-0.79<br>(-0.90 to -0.69)	-1.96<br>(-2.17 to -1.76)	-0.80<br>(-0.92 to -0.67)	-0.20<br>(-0.33 to -0.07)	-1.51<br>(-1.68 to -1.34)

United Arab Emirates	-1.20 (-1.31 to -1.09)	-1.10 (-1.19 to -1.01)	-1.25 (-1.35 to -1.15)	-0.22 (-0.72 to 0.28)	-1.51 (-2.04 to -0.98)	1.98 (1.44 to 2.53)	-1.19 (-1.55 to -0.84)	-2.02 (-2.41 to -1.63)	0.29 (-0.08 to 0.66)
United Kingdom	-1.35 (-1.47 to -1.22)	-1.24 (-1.36 to -1.13)	-1.38 (-1.50 to -1.26)	-2.02 (-2.20 to -1.84)	-1.52 (-1.69 to -1.34)	-2.28 (-2.47 to -2.09)	-2.62 (-2.78 to -2.45)	-2.31 (-2.46 to -2.15)	-2.81 (-2.98 to -2.63)
United Republic of Tanzania	-0.68 (-0.73 to -0.62)	-0.83 (-0.90 to -0.75)	-0.48 (-0.51 to -0.44)	-1.71 (-1.79 to -1.63)	-2.26 (-2.39 to -2.14)	-0.70 (-0.78 to -0.62)	-1.64 (-1.74 to -1.53)	-2.07 (-2.21 to -1.93)	-0.90 (-0.96 to -0.83)
United States of America	-0.48 (-0.67 to -0.29)	-0.10 (-0.29 to 0.10)	-0.71 (-0.90 to -0.53)	-0.46 (-0.61 to -0.30)	0.23 (0.04 to 0.43)	-0.85 (-1.01 to -0.70)	-0.91 (-1.01 to -0.82)	-0.42 (-0.52 to -0.32)	-1.22 (-1.34 to -1.11)
United States Virgin Islands	-0.82 (-0.88 to -0.76)	-0.32 (-0.37 to -0.26)	-1.14 (-1.22 to -1.07)	-2.49 (-2.65 to -2.33)	-1.73 (-1.91 to -1.55)	-3.13 (-3.32 to -2.94)	-2.22 (-2.37 to -2.07)	-1.57 (-1.76 to -1.37)	-2.82 (-2.97 to -2.68)
Uruguay	-1.06 (-1.13 to -0.98)	-1.20 (-1.26 to -1.14)	-0.99 (-1.08 to -0.90)	-1.79 (-1.91 to -1.66)	-2.05 (-2.20 to -1.91)	-1.62 (-1.76 to -1.48)	-2.16 (-2.29 to -2.03)	-2.42 (-2.61 to -2.24)	-1.99 (-2.11 to -1.87)
Uzbekistan	0.18 (0.11 − 0.26)	0.18 (0.13 − 0.23)	0.15 (0.05 − 0.24)	1.96 (1.03 − 2.90)	1.93 (1.03 − 2.84)	1.84 (0.83 − 2.85)	1.14 (0.49 − 1.80)	1.38 (0.74 − 2.03)	0.73 (0.04 − 1.43)
Vanuatu	0.01 (-0.02 to 0.05)	0.24 (0.21 − 0.28)	-0.28 (-0.32 to -0.23)	-1.04 (-1.12 to -0.95)	-0.97 (-1.04 to -0.91)	-1.19 (-1.29 to -1.09)	-0.87 (-0.94 to -0.79)	-0.76 (-0.82 to -0.70)	-1.01 (-1.11 to -0.91)
Venezuela (Bolivarian Republic of Venezuela)	-0.47 (-0.56 to -0.38)	-0.36 (-0.41 to -0.30)	-0.54 (-0.66 to -0.43)	-0.04 (-0.18 to 0.10)	0.13 (-0.01 to 0.27)	-0.18 (-0.36 to 0)	-0.34 (-0.50 to -0.18)	-0.17 (-0.31 to -0.03)	-0.47 (-0.68 to -0.26)
Viet Nam	-0.91 (-0.99 to -0.82)	-0.91 (-0.99 to -0.83)	-0.94 (-1.03 to -0.85)	-1.49 (-1.55 to -1.42)	-1.71 (-1.76 to -1.67)	-1.39 (-1.48 to -1.29)	-1.41 (-1.50 to -1.33)	-1.57 (-1.63 to -1.51)	-1.36 (-1.48 to -1.24)

Yemen	-0.86<br>(-0.95 to -0.77)	-0.93<br>(-1.02 to -0.83)	-0.88<br>(-0.96 to -0.80)	-1.50<br>(-1.67 to -1.33)	-2.00<br>(-2.22 to -1.79)	-1.17<br>(-1.32 to -1.02)	-1.73<br>(-1.89 to -1.58)	-2.02<br>(-2.21 to -1.84)	-1.52<br>(-1.65 to -1.39)
Zambia	-0.35<br>(-0.41 to -0.30)	-0.05<br>(-0.10 to 0.01)	-0.67<br>(-0.74 to -0.61)	-1.14<br>(-1.33 to -0.94)	-1.03<br>(-1.30 to -0.77)	-1.03<br>(-1.17 to -0.89)	-1.00<br>(-1.19 to -0.82)	-0.89<br>(-1.16 to -0.61)	-1.08<br>(-1.21 to -0.95)
Zimbabwe	0.98<br>(0.84 − 1.11)	0.81<br>(0.66 − 0.96)	1.15<br>(0.99 − 1.31)	2.18<br>(1.57 − 2.78)	1.64<br>(1.18 − 2.09)	2.85<br>(2.02 − 3.68)	2.38<br>(1.75 − 3.01)	1.98<br>(1.47 − 2.50)	3.00<br>(2.16 − 3.84)

ASIR age-standardized incidence rate, *ASMR* age-standardized mortality rate, *ASDR* age-standardized disability-adjusted life-year rate, *EAPC* estimated annual percentage change, *CI* confidence interval

Table S9 Attributable DALYs and age-standardized DALY rate by subarachnoid hemorrhage (SAH) risk factors in 2021

Risk factor	DALYs/10,000 (95% UI)			ASDR/100,000 persons (95% UI)		
	**Both**	**Male**	**Female**	**Both**	**Male**	**Female**
All risk factor	772.02 (651.89 − 911.45)	410.94 (324.48 − 527.11)	361.08 (300.48 − 425.34)	89.60 (75.68 − 105.80)	99.18 (78.49 − 127.01)	80.19 (66.73 − 94.57)
Environmental or occupational risks						
Particulate matter pollution	265.15 (199.42 − 350.57)	140.89 (96.62 − 206.10)	124.26 (95.64 − 164.79)	30.73 (23.13 − 40.64)	33.91 (23.29 − 49.37)	27.70 (21.33 − 36.71)
High temperature	12.07 (2.26 − 26.55)	6.74 (1.24 − 15.44)	5.33 (1.04 − 12.10)	1.43 (0.27 − 3.14)	1.64 (0.30 − 3.74)	1.23 (0.25 − 2.78)
Low temperature	47.69 (39.27 − 57.66)	24.85 (19.65 − 31.35)	22.84 (19.07 − 27.76)	5.60 (4.62 − 6.77)	6.13 (4.86 − 7.69)	5.08 (4.24 − 6.17)
Lead exposure	67.35 (-8.95 to 149.46)	38.42 (-5.18 to 87.92)	28.93 (-3.77 to 66.13)	7.76 (-1.03 to 17.25)	9.29 (-1.25 to 21.11)	6.37 (-0.83 to 14.58)
Behavioral risks						
Tobacco						
Smoking	156.74 (127.76 − 189.74)	128.68 (102.79 − 160.42)	28.06 (23.72 − 33.44)	18.07 (14.72 − 21.86)	30.41 (24.29 − 37.81)	6.31 (5.34 − 7.53)
Secondhand smoke	50.16 (33.62 − 67.34)	21.52 (13.58 − 30.83)	28.63 (19.24 − 39.43)	5.84 (3.91 − 7.85)	5.16 (3.25 − 7.36)	6.48 (4.35 − 8.93)

Dietary risks						
Diet high in red meat	-75.31 (-310.07 to 105.58)	-39.22 (-161.71 to 54.35)	-36.09 (-148.10 to 49.40)	-8.77 (-36.12 to 12.25)	-9.31 (-38.32 to 12.86)	4.61 (-1.33 to 9.91)
Diet high in sodium	97.60 (22.38 − 224.36)	58.62 (15.38 − 130.80)	38.98 (6.96 − 96.26)	11.19 (2.54 − 25.86)	13.98 (3.62 − 31.27)	1.56 (-0.16 to 3.29)
Diet low in fiber	42.89 (-12.72 to 91.25)	22.80 (-6.79 to 50.06)	20.08 (-5.78 to 43.34)	5.03 (-1.50 to 10.68)	5.43 (-1.62 to 11.90)	8.56 (1.52 to 21.25)
Diet low in fruits	96.97 (-6.90 to 182.02)	52.51 (-3.51 to 103.08)	44.45 (-3.39 to 84.84)	11.29 (-0.80 to 21.15)	12.47 (-0.83 to 24.42)	-8.22 (-33.86 to 11.24)
Diet low in vegetables	15.52 (-1.63 to 32.52)	8.74 (-0.95 to 21.43)	6.78 (-0.70 to 14.30)	1.82 (-0.19 to 3.81)	2.08 (-0.23 to 5.10)	10.12 (-0.78 to 19.26)
Metabolic risks						
High body mass index	52.97 (0.04 − 119.13)	22.72 (0.05 − 51.47)	30.25 (-0.49 − 67.11)	6.14 (0 − 13.85)	5.35 (0.01 − 12.19)	6.88 (-0.11 to 15.34)
High systolic blood pressure	557.89 (404.92 − 706.24)	288.50 (203.41 − 396.09)	269.39 (195.49 − 339.64)	64.58 (46.84 − 81.79)	69.68 (49.26 − 95.18)	59.38 (43.07 − 75.07)
Risks cluster						
Environmental or occupational risks	353.50 (264.48 − 458.64)	188.90 (128.62 − 267.48)	164.59 (125.28 − 209.95)	41.05 (30.76 − 53.24)	45.66 (31.25 − 64.55)	36.67 (27.90 − 46.93)
Behavioral risks	311.33 (207.91 − 432.03)	204.54 (94.01 − 321.00)	107.18 (55.72 − 166.64)	35.96 (23.98 − 49.92)	48.52 (34.40 − 67.23)	24.02 (12.44 − 37.22)

Metabolic risks	568.15 (415.24 − 712.30)	292.73 (205.40 − 399.32)	275.41 (202.87 − 344.89)	65.78 (48.06 − 82.57)	70.69 (49.75 − 95.87)	60.78 (44.72 − 76.31)

DALYs disability-adjusted life-years, *ASDR* age-standardized disability-adjusted life-year rate, *UI* uncertainty interval

Table S10 Attributable deaths and age-standardized deaths rate by subarachnoid hemorrhage (SAH) risk factors in 2021

Risk factor	Deaths/1000 (95% UI)			ASMR/100,000 persons (95% UI)		
	**Both**	**Male**	**Female**	**Both**	**Male**	**Female**
All risk factor	263.31 (222.15 − 312.98)	133.55 (104.18 − 171.73)	129.76 (106.33 − 155.40)	3.10 (2.60 − 3.69)	3.40 (2.67 − 4.35)	2.82 (2.31 − 3.38)
Environmental or occupational risks						
Particulate matter pollution	88.43 (65.26 − 116.47)	45.52 (30.32 − 66.43)	42.91 (32.43 − 57.60)	1.04 (0.76 − 1.36)	1.15 (0.77 − 1.67)	0.94 (0.71 − 1.26)
High temperature	3.89 (0.59 − 8.91)	2.09 (0.30 − 4.93)	1.80 (0.27 − 4.26)	0.05 (0.01 − 0.10)	0.05 (0.01 − 0.12)	0.04 (0.01 − 0.09)
Low temperature	19.66 (16.27 − 23.20)	9.48 (7.51 − 11.75)	10.17 (8.36 − 12.45)	0.23 (0.19 − 0.28)	0.25 (0.20 − 0.31)	0.22 (0.18 − 0.27)
Lead exposure	24.39 (-3.24 to 54.01)	13.61 (-1.82 to 31.11)	10.78 (-1.42 to 24.54)	0.29 (-0.04 to 0.63)	0.35 (-0.05 to 0.79)	0.23 (-0.03 to 0.53)
Behavioral risks						
Tobacco						
Smoking	45.07 (35.70 − 55.90)	37.09 (28.79 − 47.39)	7.98 (6.69 − 9.61)	0.52 (0.41 − 0.64)	0.91 (0.70 − 1.15)	0.18 (0.15 − 0.21)
Secondhand smoke	14.71 (9.78 − 19.92)	6.21 (3.83 − 8.83)	8.50 (5.61 − 11.85)	0.17 (0.11 − 0.23)	0.16 (0.10 − 0.22)	0.19 (0.12 − 0.26)

Dietary risks						
Diet high in red meat	-20.20<br>(-81.67 to 28.75)	-10.36<br>(-41.79 to 14.54)	-9.83<br>(-39.11 to 14.23)	-0.24<br>(-0.95 to 0.34)	-0.25<br>(-1.01 to 0.35)	-0.22<br>(-0.87 to 0.31)
Diet high in sodium	33.46<br>(7.10 − 77.88)	19.64<br>(4.86 − 43.44)	13.82<br>(2.27 − 34.87)	0.39<br>(0.08 − 0.91)	0.49<br>(0.12 − 1.09)	0.30<br>(0.05 − 0.76)
Diet low in fiber	11.25<br>(-3.17 to 24.64)	5.75<br>(-1.62 to 12.74)	5.50<br>(-1.54 to 12.13)	0.13<br>(-0.04 to 0.29)	0.14<br>(-0.04 to 0.31)	0.12<br>(-0.03 to 0.27)
Diet low in fruits	26.51<br>(-1.76 to 51.15)	13.99<br>(-0.87 to 27.65)	12.52<br>(-0.89 to 24.09)	0.31<br>(-0.02 to 0.60)	0.34<br>(-0.02 to 0.67)	0.28<br>(-0.02 to 0.53)
Diet low in vegetables	3.99 (-<br>0.38 to 8.69)	2.18 (-<br>0.23 to 5.70)	12.52<br>(-0.89 to 24.09)	0.05<br>(0 − 0.10)	0.05<br>(-0.01 to 0.14)	0.04<br>(0 − 0.09)
Metabolic risks						
High body mass index	13.84<br>(0.01 − 30.82)	5.76<br>(0.01 − 13.29)	8.08<br>(-0.12 − 17.84)	0.16<br>(0 − 0.36)	0.14<br>(0 − 0.32)	0.18<br>(0 − 0.40)
High systolic blood pressure	196.71<br>(143.36 − 249.92)	96.24<br>(68.20 − 130.83)	100.47<br>(73.05 − 127.70)	2.32<br>(1.69 − 2.94)	2.46<br>(1.75 − 3.32)	2.18<br>(1.58 − 2.76)
Risks cluster						
Environmental or occupational risks	122.02<br>(90.20 − 157.47)	62.83<br>(41.30 − 89.20)	59.19<br>(44.31 − 75.36)	1.43<br>(1.06 − 1.85)	1.60<br>(1.06 − 2.27)	1.29<br>(0.97 − 1.65)
Behavioral risks	94.52<br>(60.01 − 136.87)	61.02<br>(41.72 − 85.66)	33.50<br>(17.21 − 55.33)	1.10<br>(0.69 − 1.59)	1.51<br>(1.02 − 2.13)	0.73<br>(0.38 − 1.21)

Metabolic risks	199.17 (146.29 − 252.06)	97.25 (69.01 − 131.59)	101.92 (74.81 − 129.03)	2.34 (1.72 − 2.97)	2.48 (1.77 − 3.35)	2.21 (1.62 − 2.80)

ASMR age-standardized mortality rate, *UI* uncertainty interval

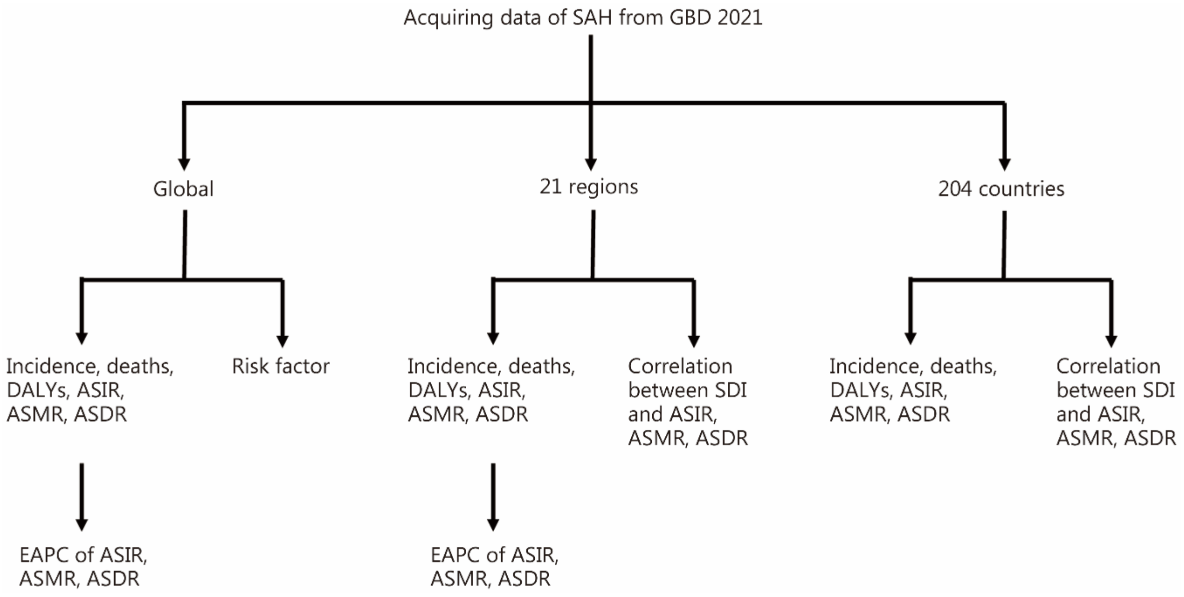

Fig. S1 Flowcharting of the analysis process. GBD Global Burden of Disease Study, SAH subarachnoid hemorrhage, DALYs disability-adjusted life-years, ASIR age-standardized incidence rate, ASMR age-standardized mortality rate, ASDR age-standardized disability-adjusted life-year rate, EAPC estimated annual percentage change, SDI sociodemographic index

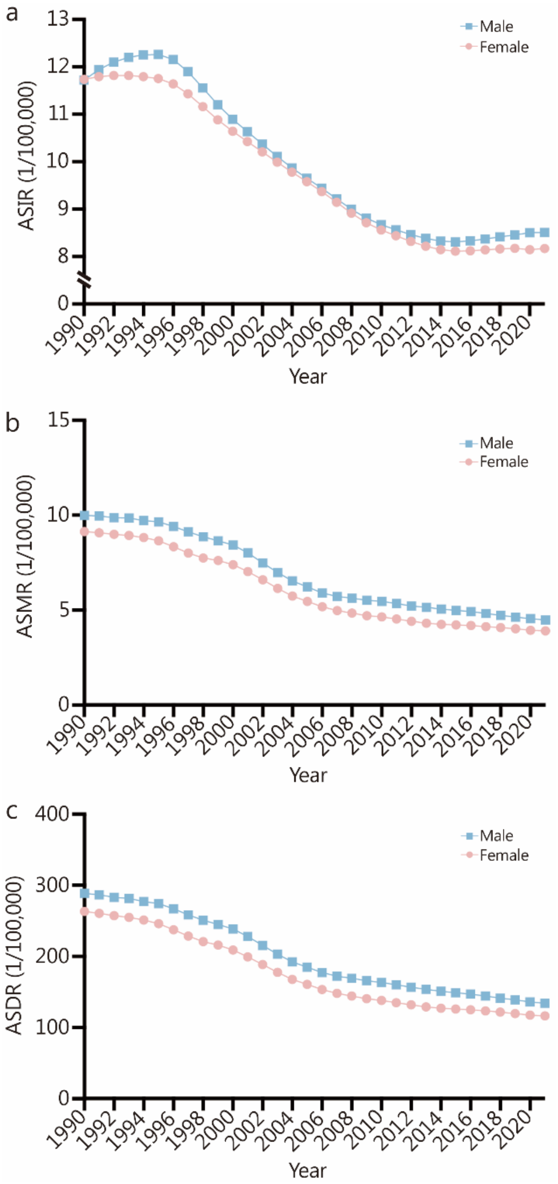

Fig. S2 The age-standardized rates of SAH during 1990 − 2021 by sex. **a** The global ASIR of SAH during 1990 − 2021 by sex. **b** The global ASMR of SAH during 1990 − 2021 by sex. **c** The global ASDR of SAH during 1990 − 2021 by sex. ASIR age-standardized incidence rate, ASMR age-standardized deaths rate, ASDR age-standardized disability-adjusted life-year rate, SAH subarachnoid hemorrhage

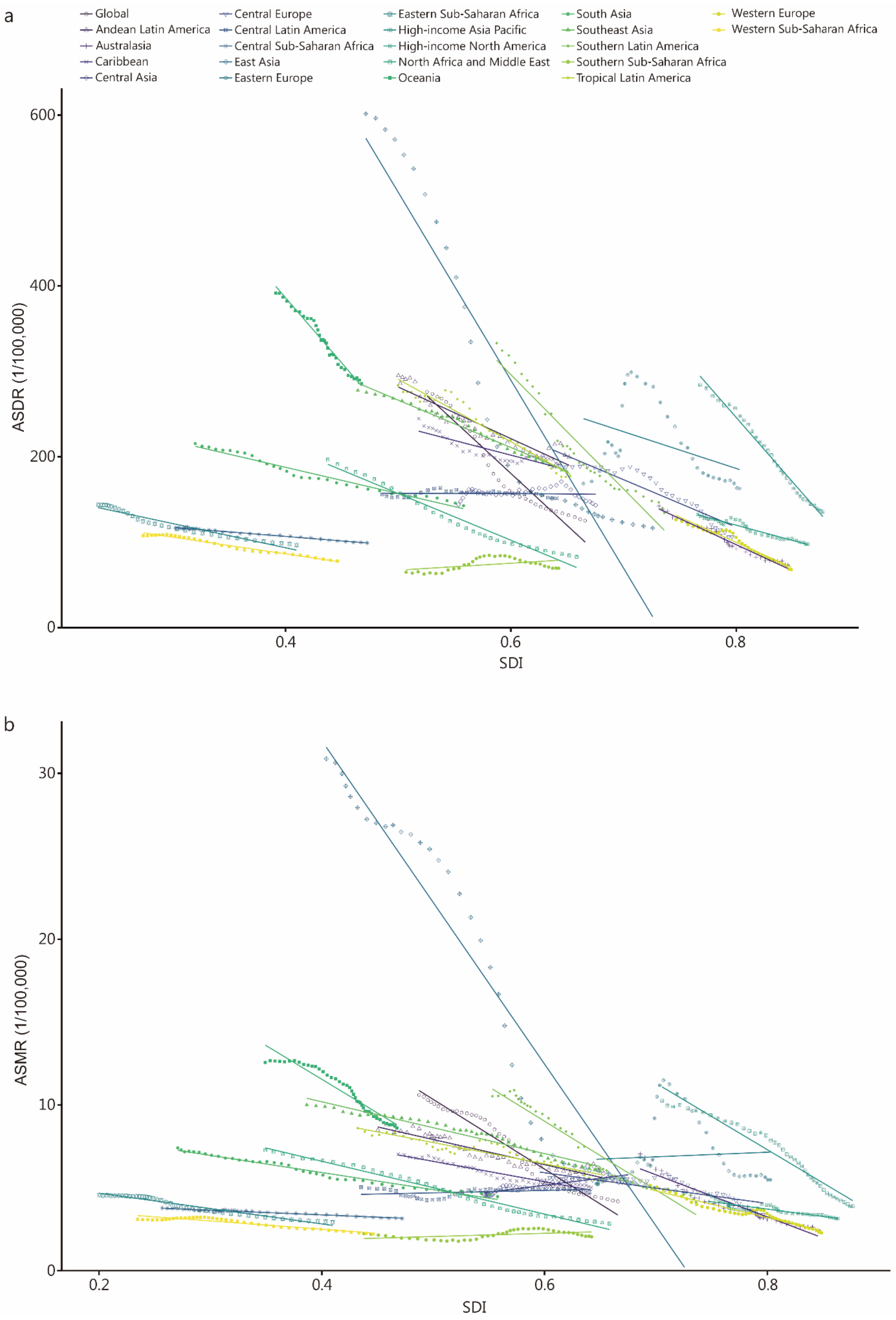

Fig. S3 ASMR and ASDR of SAH for 21 regions and 204 countries and territories by SDI. **a** ASMR for 21 regions by SDI from 1990 to 2021. **b** ASDR for 21 regions by SDI from 1990 to 2021. ASMR age-standardized death rate, ASDR age-standardized disability-adjusted life-year rate, SDI sociodemographic index, SAH subarachnoid hemorrhage

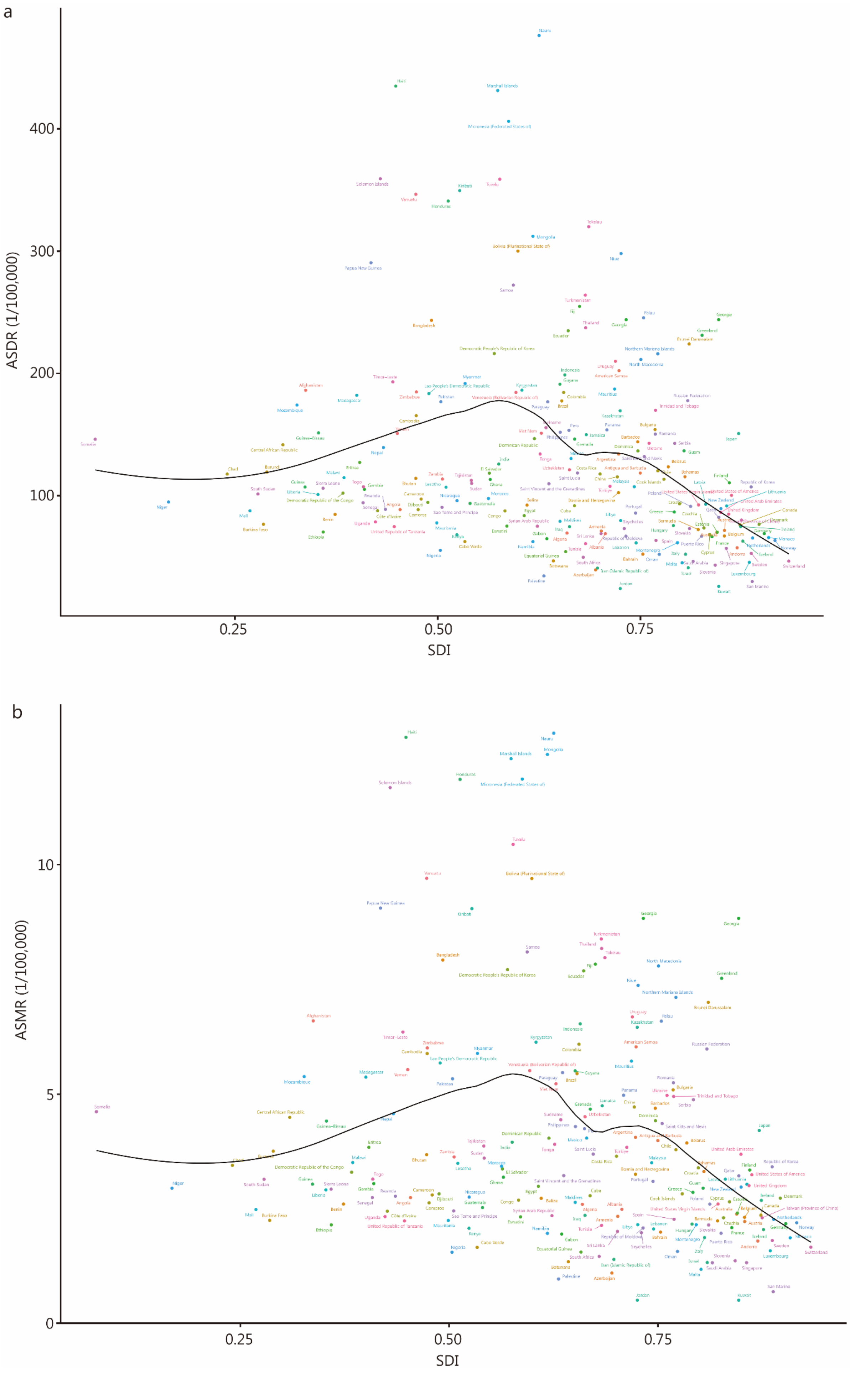
a
ASDR (1/100,000)
400
300
200
100
0.25
0.50
0.75
SDI
b
ASMR (1/100,000)
10
5
0
0.25
0.50
0.75
SDI

Fig. S4 ASMR and ASDR of SAH for 204 countries and territories by SDI in 2021. **a** ASMR for 204 countries and territories by SDI in 2021. **b** ASDR for 204 countries and territories by SDI in 2021. ASMR age-standardized death rate, ASDR age-standardized disability-adjusted life-year rate, SDI sociodemographic index, SAH subarachnoid hemorrhage

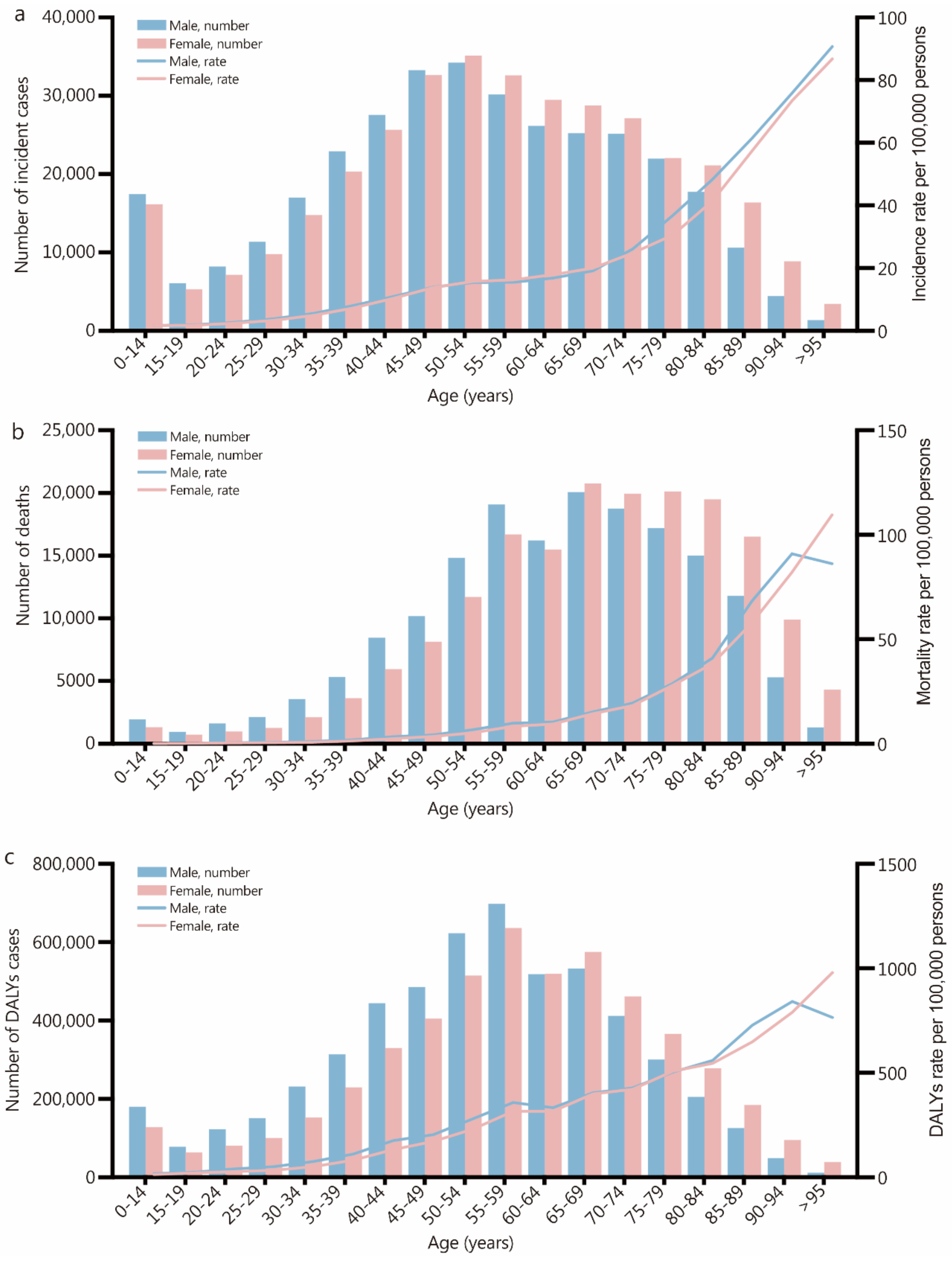

Fig. S5 The global incidence, number of deaths, and DALYs due to SAH by age and sex. **a** The number and rate of incidence of SAH in 2021 by sex and age. **b** The number and rate of deaths of SAH in 2021 by sex and age. **c** The number and rate of DALYs of SAH in 2021 by sex and age. DALYs disability-adjusted life-years, SAH subarachnoid hemorrhage

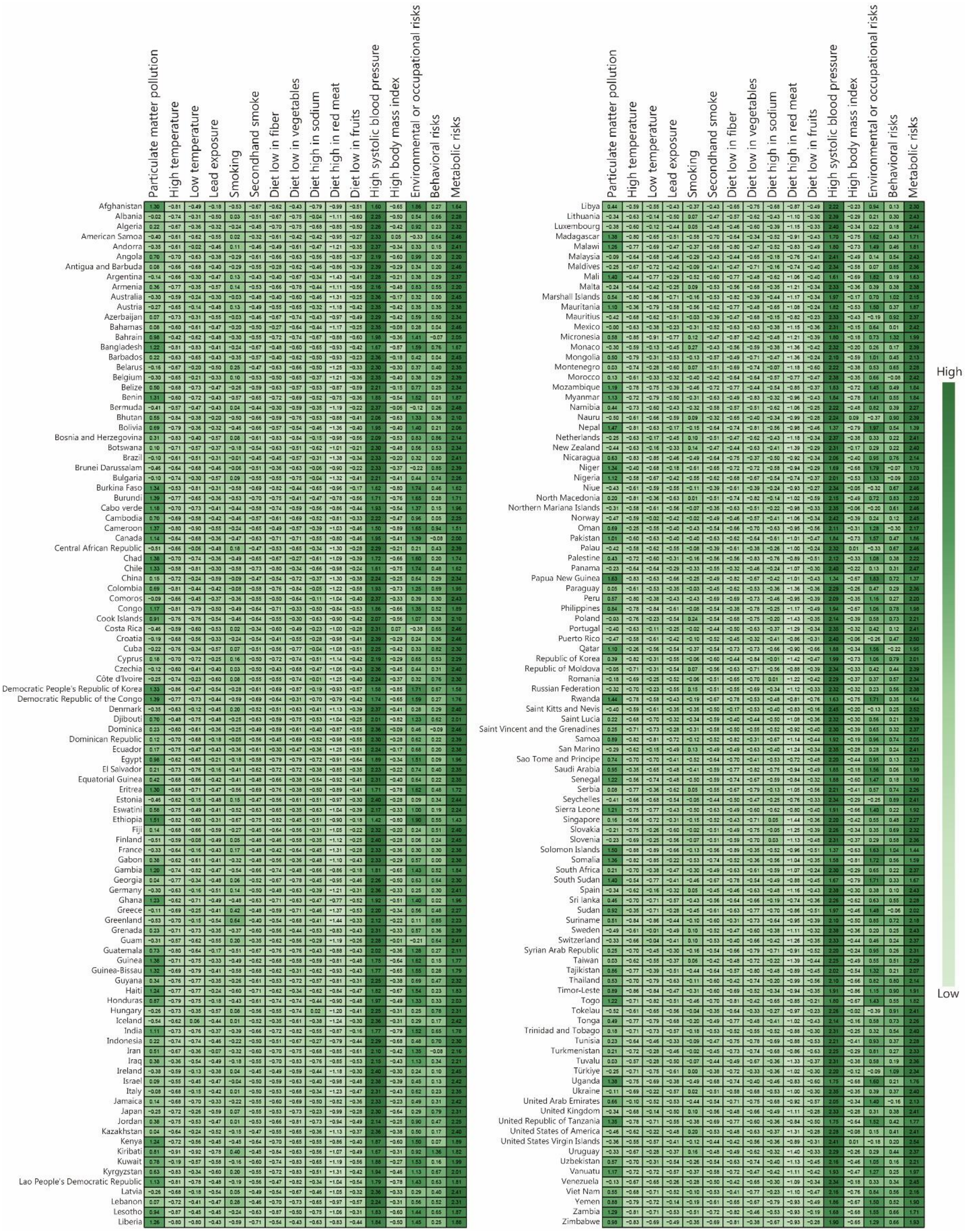

Fig. S6 Attributable age-standardized death rate by SAH risk factors in 204 regions or countries in 2021. The darker the green color means the higher the value. SAH subarachnoid hemorrhage
